# Dysfunction of the mTOR pathway is a risk factor for Alzheimer’s disease

**DOI:** 10.1186/2051-5960-1-3

**Published:** 2013-05-08

**Authors:** Sharon C Yates, Amen Zafar, Paul Hubbard, Sheila Nagy, Sarah Durant, Roy Bicknell, Gordon Wilcock, Sharon Christie, Margaret M Esiri, A David Smith, Zsuzsanna Nagy

**Affiliations:** 1 Neuropharmacology and Neurobiology, College of Medical and Dental Sciences, School of Clinical and Experimental Medicine, University of Birmingham, Birmingham B15 2TT, UK; 2 Institute of Biomedical Research, College of Medical and Dental Sciences, University of Birmingham, Birmingham B15 2TT, UK; 3 OPTIMA, University of Oxford, Level 4, John Radcliffe Hospital, Oxford OX3 9DU, UK; 4 Department of Neuropathology, University of Oxford, Level 1, John Radcliffe Hospital, Oxford OX3 9DU, UK; 5 Department of Pharmacology, University of Oxford, Mansfield Road, Oxford OX1 3QT, UK

**Keywords:** mTOR, cell cycle, Alzheimer’s disease, risk factor, ApoE

## Abstract

**Background:**

The development of disease-modifying therapies for Alzheimer’s disease is hampered by our lack of understanding of the early pathogenic mechanisms and the lack of early biomarkers and risk factors.

We have documented the expression pattern of mTOR regulated genes in the frontal cortex of Alzheimer’s disease patients. We have also examined the functional integrity of mTOR signaling in peripheral lymphocytes in Alzheimer’s disease patients relative to healthy controls.

**Results:**

In the brain mTOR is seen to control molecular functions related to cell cycle regulation, cell death and several metabolic pathways. These downstream elements of the mTOR signaling cascade are deregulated in the brain of Alzheimer’s disease patients well before the development of pathology. This dysregulation of the mTOR downstream signaling cascade is not restricted to the brain but appears to be systemic and can be detected in peripheral lymphocytes as a reduced Rapamycin response.

**Conclusions:**

The dysfunction of the signaling pathways downstream of mTOR may represent a risk factor for Alzheimer’s disease and is independent of the ApoE status of the patients.

We have also identified the molecular substrates of the beneficial effects of Rapamycin on the nervous system. We believe that these results can further inform the development of clinical predictive tests for the risk of Alzheimer’s disease in patients with mild cognitive impairment.

## Background

Late-onset Alzheimer’s disease (AD) has become the silent epidemic of the 21^st^ century and the effort to understand the disease process and develop early diagnostic and therapeutic interventions has been enormous. The failure to find effective drugs based on the amyloid cascade hypothesis has prompted the recent re-evaluation of the leading theory for the pathogenesis of AD [1,2].

A more recent alternative hypothesis for the pathogenesis of AD involves the cell cycle. The hypothesis postulates that AD is the consequence of aberrant re-entry of neurones into the cell division cycle and subsequent regulatory failure [3,4]. Whilst neurons were historically thought to be terminally differentiated, there is now a considerable body of evidence to indicate that neurons in fact are able to re-enter the cell cycle (reviewed in [3]). Whether cell cycle re-entry is part of the normal life-cycle of a healthy neuron, or is restricted to diseased neuronal states, remains unclear [3,5,6]. Nevertheless, healthy neurons are not thought to pass the G1/S checkpoint and do not replicate their DNA. The qualitative jump from healthy ageing to Alzheimer’s disease appears to be the neuronal commitment to DNA replication and subsequent entry into the G2 phase (reviewed in [7]). Although the involvement of the cell cycle in the pathogenesis of AD explains many features of the disease unsolved by previous hypotheses (reviewed in [3,6,7] it also raises many questions. Most importantly, what are the triggers and regulators of the cell division cycle that drive the unsuccessful proliferation-attempt in neurons and how do these relate to ApoE, the strongest genetic risk factor for AD?

**Figure 1:**
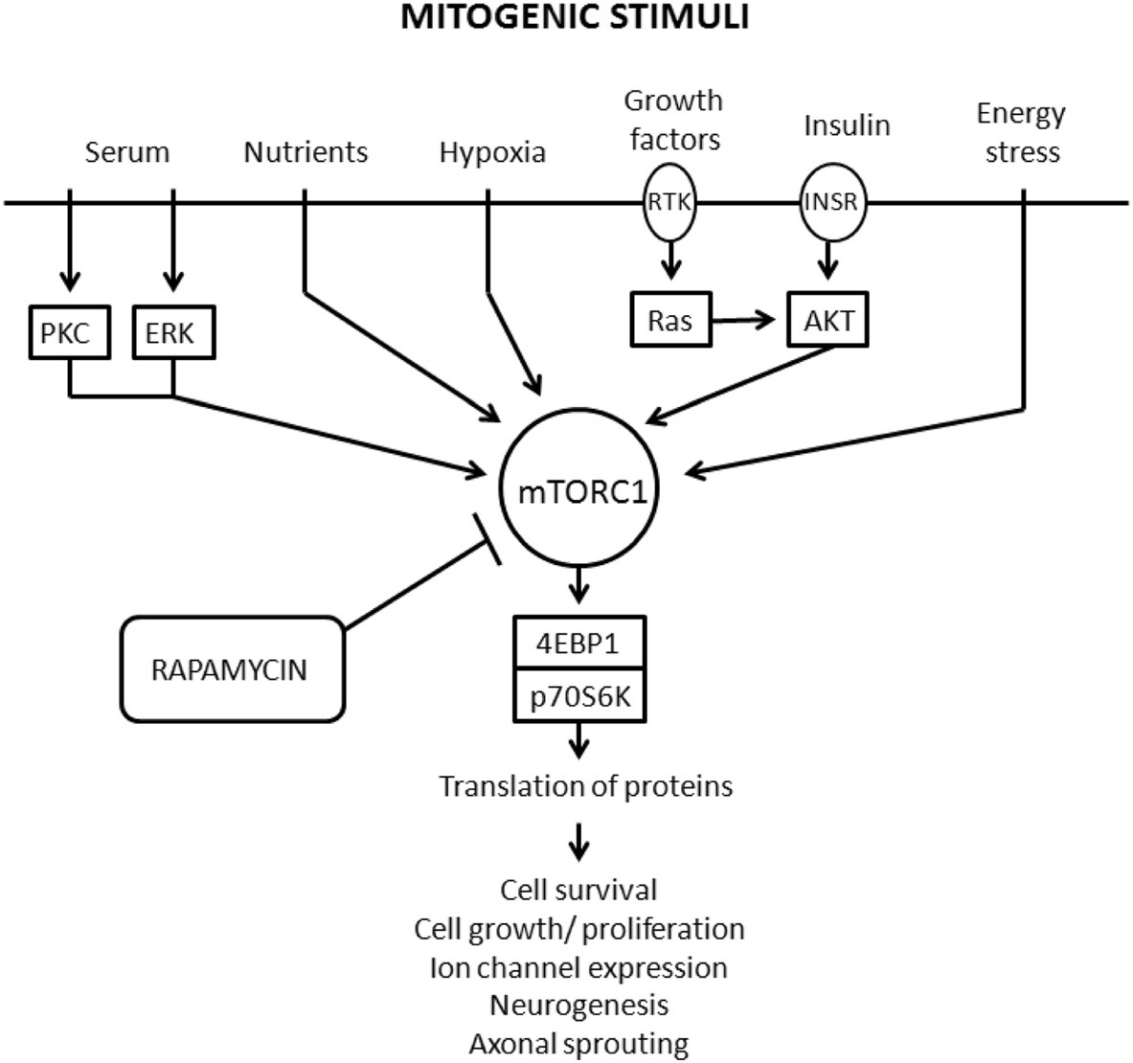
**A simplified version of the mTOR signalling pathway.** MTORC1 receives and integrates the signals from various upstream pathways, including pathways triggered by nutrients, growth factors, hypoxia and insulin that promote cell growth, division and differentiation. Rapamycin is a powerful inhibitor of mTORC1 activity. Abbreviations: 4E-binding protein (4EBP1); eukaryotic translation initiation factor 4E (eIF4E); Receptor Tyrosine Kinase (RTK); Insulin receptor (INSR); Protein Kinase C (PKC); Extracellular Signal Regulated Kinase (ERK); (Ras); Protein kinase B (AKT).

The mammalian target of rapamycin (mTOR) is a highly conserved serine-threonine kinase that is essential for the co-ordination of intra and extra-cellular signals concerning cell growth, division and differentiation [8]. It is found in two complexes within the cell: mTORC1 and mTORC2, which act upstream of the complex mTOR pathway. MTORC1, responsible for co-ordinating growth factor dependent growth and proliferation, is turned on in response to various upstream signals such as growth factors and nutrients (summarised in Figure 1) and is inhibited by the naturally occurring macrolide rapamycin [9].

In the nervous system the mTOR pathway is known to play a key role in regulating synaptic remodelling and long term potentiation (LTP) [10-12]. The overactivation of the mTOR signalling pathway on the other hand has been implicated in the formation of erroneous connections between neurons in epilepsy [13-15] and after trauma [16,17]. Furthermore, the mTOR pathway plays a key role in regulating autophagy in neurons: one of the mechanisms by which cells turn over proteins and organelles [12,18]. In recent years our understanding of the role of mTOR in physiology and pathology of the nervous system has dramatically increased. However, we do not have a full inventory of the downstream effectors of mTOR in the CNS and do not understand how modifications in mTOR activity contribute to neurodegeneration [19].

The first evidence to indicate that mTOR signalling is altered in AD was our finding of a reduced Rapamycin response in AD patients relative to healthy controls [20]. Later, the alteration in mTOR signalling in lymphocytes were found to be associated with cognitive decline in AD [21] and the reduced responsiveness to Rapamycin was attributed to conformational changes in p53 [22].

A number of studies have since shown an up-regulation of various components of the mTOR pathway, downstream of mTORC1, in the brain of AD patients [23-25]. There is an association between the presence of these activated components and the accumulation of phospho-tau (p-tau) and neurofibrillary tangles (NFT) in neurons [23-25]. It was concluded that the mTOR pathway mediates tau phosphorylation [26,27], the first step in the production of the intracellular NFT associated with AD. There is also evidence of an association between mTOR signalling and beta-amyloid, although the nature of the association is uncertain [18,28,29]. Rapamycin induced inhibition of mTORC1 has also been shown to decrease beta-amyloid expression by increasing the rate of autophagy [18,29]. As MTORC1 is a negative regulator of autophagy, the increased mTOR signalling associated with AD may reduce the rate of autophagy, potentially leading to an accumulation of the hallmarks of AD, secondary to reduced clearing. More recently in animal models Rapamycin has been found to be a good candidate for the treatment of AD [29].

The aims of this study were to identify the Rapamycin-regulated genes that are differentially expressed in Alzheimer’s disease brain and to evaluate the usefulness of the Rapamycin test in lymphocytes as a risk factor for Alzheimer’s disease.

## Methods

Peripheral blood from healthy controls, used to standardise and determine the analytical performance of the Rapamycin assay, was provided by the NHS blood and transfusion centre (ethical approval no: 07/Q2707/97). Blood from this source was also used for the determination of rapamycin-regulated genes in lymphocytes by gene expression microarray.

Peripheral blood from elderly subjects was provided by the Oxford Project to Investigate Memory and Ageing (OPTIMA) (ethical approval no: 09/H0107/9 and 18/07/2007). The samples provided were from Alzheimer patients (n=51, age range=57-92, mean age=79) who fulfilled the NINCDS-ARDRA criteria for probable AD [30] or patients with mild cognitive impairment (MCI, n=27, age=69-95, mean age=83) fulfilling the Petersen criteria [31]. Additional samples were collected from healthy age matched controls (n=68, age range=60-94, mean age=79.5). Appropriate consent and ethical approval was sought prior to the start of the study.

Frozen post-mortem brain tissue (frontal lobe) of 41 AD sufferers (Braak stage III-IV: n=19; Braak stage V-VI: n=22) and 5 elderly controls was provided by the Thomas Willis brain bank (ethical approval no: 07/Q2707/98). The included patients had no other pathology than AD that was thought to contribute to the severity of dementia, and had plasma homocysteine levels below 35 μM prior to death. The severity of AD was classified on the basis of the Braak staging [32]. For the sake of simplicity we use the terminology of ‘limbic stage AD’ for patients in Braak stages III-IV and ‘neocortical stage AD’ for patients in Braak stage V-VI, as proposed by Braak [32]. Patient selection was guided by quantitative assessment of the AT8-positive phospho tau, DC11-positive tau and beta-amyloid (performed previously) (Additional file 1: Figure S1, Figure S2 and Figure S3) to ensure that the groups selected were homogenous in terms of pathology and that limbic stage patients did not have significant amyloid deposition in the brain region studied relative to controls. The number of cases included in the study was based on availability, not statistical power calculations. Additional details on patient data can be found in Additional file 2: Table S1.

### Lymphocyte separation and cell culture

Peripheral blood was collected in Heparin vacutainers and shipped at room temperature. The separation of lymphocytes was carried out within 24 hours of blood collection using established protocols (Lymphoprep, Axis-Shield, NYC-1114545). The separated and washed lymphocytes were re-suspended in 9:1 FCS: DMSO and stored at −70°C (at least 24 hours) before placing into liquid nitrogen.

Frozen lymphocytes were thawed in batches and washed in RPMI-1640 to remove freezing medium. The cells were then cultured (1million/ml density) in RPMI-1640 medium supplemented with L-glutamine (2%), Penicillin/Streptomicin (1%), PHA (2.5% of PAA PHA) and FCS (15%) for 48 hours. From each patient two sets of identical cultures were set up in 96 well plates (6 wells in each set). Following the initial 48 hours culture half of the cultures from each set were treated with Rapamycin (100 ng/ml) while half were treated with DMSO alone (solvent for Rapamycin) for a further 24 hours. At the end of the treatment period (total 72 hours in culture) one set of cultures was collected for LDH assay by freezing the samples at -20°C. The second set of cultures was fixed with ice-cold 85% ethanol and stored at 4°C for flow cytometry.

### Assessment of cell numbers and cell cycle kinetics in cultured lymphocytes

Overall cell numbers were determined using a commercially available kit (CytoTox 96 non-radioactive Cytotoxicity Assay, Promega) that quantitatively measures lactate dehydrogenase (LDH) release upon cell lysis [33]. LDH positive control was used to generate a calibration curve. Lymphocytes were lysed by freezing at −20°C for at least overnight. Samples were thawed and kept at 4°C before use. 30 μl of culture was distributed into 96 well plates; each sample was represented in triplicates. 50 μl of ice-cold reconstituted substrate buffer was added to each well. Plates were incubated at room temperature and protected from light. Reaction was stopped with 50 μl of stop solution and absorbance read at 490 nm. Cell numbers are directly proportional to the absorbance values which represent LDH activity. The quality standards for the LDH assay are included in the supplementary methods (Additional file 3).

From each culture the LDH assay was carried out in triplicate (while each culture condition was repeated in three technical replicates as well). The coefficient of variance (CV) of the LDH carried out from the same culture sample was consistently <10%, while the CV from the triplicate cultures was also <10%. The assay generated three variables for each patient: cell numbers at the time of culture seeding and the cell numbers after three days in culture with and without Rapamycin.

Cell cycle analysis was carried out to analyse the effect of cell cycle inhibitor drugs on the cell cycle kinetics of lymphocytes. The assessment relies on the measurement of Propidium iodide (PI) incorporation into DNA. The fixed lymphocytes were washed with ice cold PBS and stained with propidium iodide (PI 20 μg/ml; RNAs A 100 20 μg/ml and 0.1% Triton X in PBS) for minimum 30 minutes at room temperature. Cytometry was carried out with a BD FacsCalibur cytometer fitted with a 96 well High Throughput Sampler. Quality standards are included in the supplementary methods (Additional file 3). The cytometry allowed the recording of cell fractions in different phases of the cell cycle [34], apoptotic fraction and non-dividing fraction of the cell populations both in control and Rapamycin treated cultures from each patient.

Using the variables recorded from the first 47 control subjects and 27 AD patients we performed logistic regression to identify the variables that best predicted patient diagnosis. The identified variables were then used to calculate the probability of AD in any given patient (probability of AD based on Ly test). This probability value then was analysed using ROC curve analysis to determine the specificity and sensitivity of the assay as well as the positive likelihood ratio at any given test results. The same calculation was then applied to the subsequent 21 controls and 24 AD patients to check assay performance in an independent patient group.

The probability of a patient having AD just based on age was regarded as the pre-test probability of a patient having AD. Published likelihood ratios were used to calculate the post-test probability after an ApoE genotype determination. The likelihood ratio obtained for any given lymphocyte-test result (from the ROC curve) was used to calculate the post-test probability of AD based on the lymphocyte test. The comparison of pre-test and post test probabilities in our patients was used as a measure of improvement in the diagnostic prediction achieved by the use of the lymphocyte test.

### RNA extraction for microarray

To identify the genes regulated by the mTOR pathway lymphocytes from young healthy donors were cultured with and without rapamycin. The expected rapamycin response was a reduction in cell proliferation and accumulation of cells in the G1 phase relative to non-treated cultures from the same individual. Lymphocytes were cultured as described above. Total RNA was extracted with TRI-reagent (Sigma) according to the standard protocol and treated with DNase (Qiagen). Total RNA was also extracted from the post mortem brain tissue as described above.

The quality of the RNA was assessed on the Agilent 2100 Bioanalyser (see Additional file 3). Agilent microarrays were used for expression analysis in order to minimise the effect of RNA quality on the results [35-38] (see Additional file 3).

### Microarray

Two-colour microarray based gene expression analysis (Agilent Technologies) was used to identify genes that were differentially expressed in rapamycin-treated lymphocytes compared to untreated lymphocytes following the standard protocol (Agilent manual: G4140-90050). Briefly, 200 ng of RNA was amplified by conversion to cDNA, and subsequently to labelled cRNA, with the Low-Input Quick Amplification Labelling kit (Agilent Technologies). Samples from rapamycin-treated lymphocytes and untreated lymphocytes were labelled with Cyanine-3 and Cyanine-5 respectively. Labelled cRNA was purified with the RNeasy mini column (Qiagen) and quantified with a nanodrop Spectrophotometer. The two samples were mixed (825 ng each), fragmented, and hybridised to the 4x44K Agilent Whole Human Genome Microarray at 65°C for 17 hours. The microarrays were washed and scanned with the Agilent C Scanner on the AgilentHD_GX_2color setting.

8×15 K Custom microarrays (Agilent Technologies) were designed to include the identified 1127 rapamycin-regulated genes in lymphocytes and 38 known rapamycin-regulated genes based on an IPA Ingenuity search (http://www.ingenuity.com) (total 1165 genes). Various housekeeping genes (719 probes) and internal controls (3141 probes) were also included. One-colour microarray based gene expression analysis (Agilent Technologies) was used to identify rapamycin-regulated genes that were differentially expressed in brain affected by AD compared to control. The one-colour microarray protocol is described in manual: G4140-90040. Briefly, 200 ng of RNA per subject was converted to Cyanine-3 labelled cRNA with the Low Input Quick Amplification Labelling kit (Agilent Technologies). After purification and quantification as described previously, 600ng of cRNA per subject was fragmented and hybridised to an 8×15 K Custom microarray. The microarrays were scanned on programme AgilentHD_GX_1color.

### Microarray data analysis

The feature extraction programme was used to collate the Microarray layouts with the output of the scanner. The output from the 4×44 K Whole Human Genome microarrays were processed to identify rapamycin-regulated genes in lymphocytes; whereas the output of the 8×15 K custom microarrays were processed to determine the expression level of the rapamycin-regulated genes in the frontal lobe of each subject. The custom microarray outputs were grouped according to AD severity [32]. Statistical analysis of gene expression was carried out only for the 1165 rapamycin-regulated genes. We used the ‘Statistical analysis of microarray’ software (SAM) to carry out unpaired, two sample T-tests for each gene in a group-of-interest compared to control. Genes with an estimated false discovery rate (FDR) <10% were considered differentially expressed. SAM was carried out with 1000 permutations and the output processed to remove duplicates. The functional analysis of the genes differentially regulated was carried out with IPA Ingenuity. Cluster and TreeView programs were used for the analysis and visualization of the microarray results [39].

The validation of the Microarray results was carried out by Q-PCR for six genes (see Additional file 3 and Additional file 2: Table S2.).

## Results

### Rapamycin-regulated genes in normal lymphocytes associated with G1 inhibition

Two-colour Agilent gene expression array analysis identified 1127 genes whose expression was regulated by rapamycin in lymphocytes (FDR<10%). Of the 1127 genes, 1033 were up-regulated and 94 genes down-regulated in response to rapamycin.

**Figure 2:**
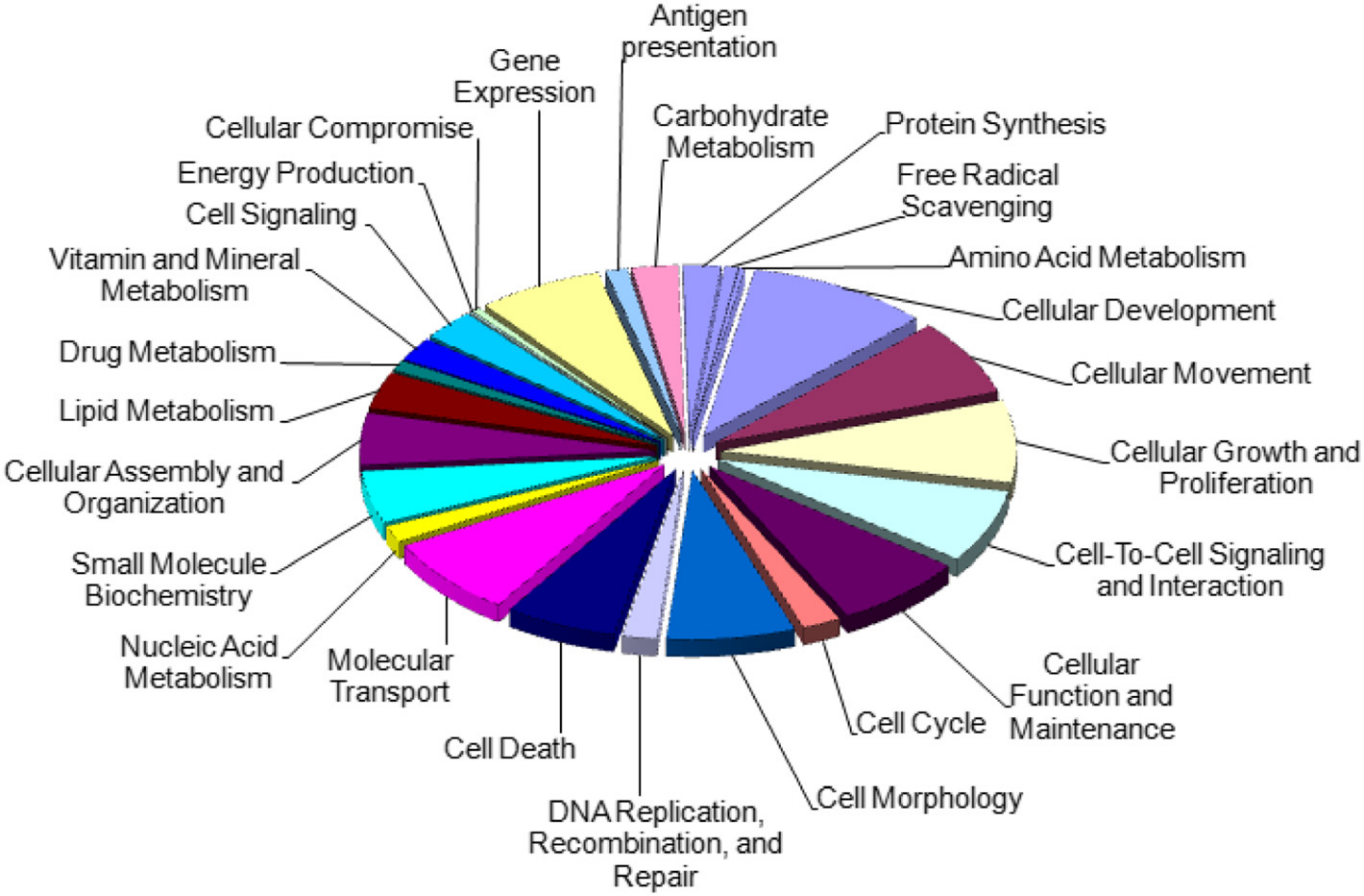
**Molecular and cellular functions associated with genes up-regulated in lymphocytes in response to rapamycin treatment.** The chart shows the number of genes, expressed as a percentage of the total number, involved in each of the molecular and cellular functions deemed significant by IPA Ingenuity.

**Figure 3:**
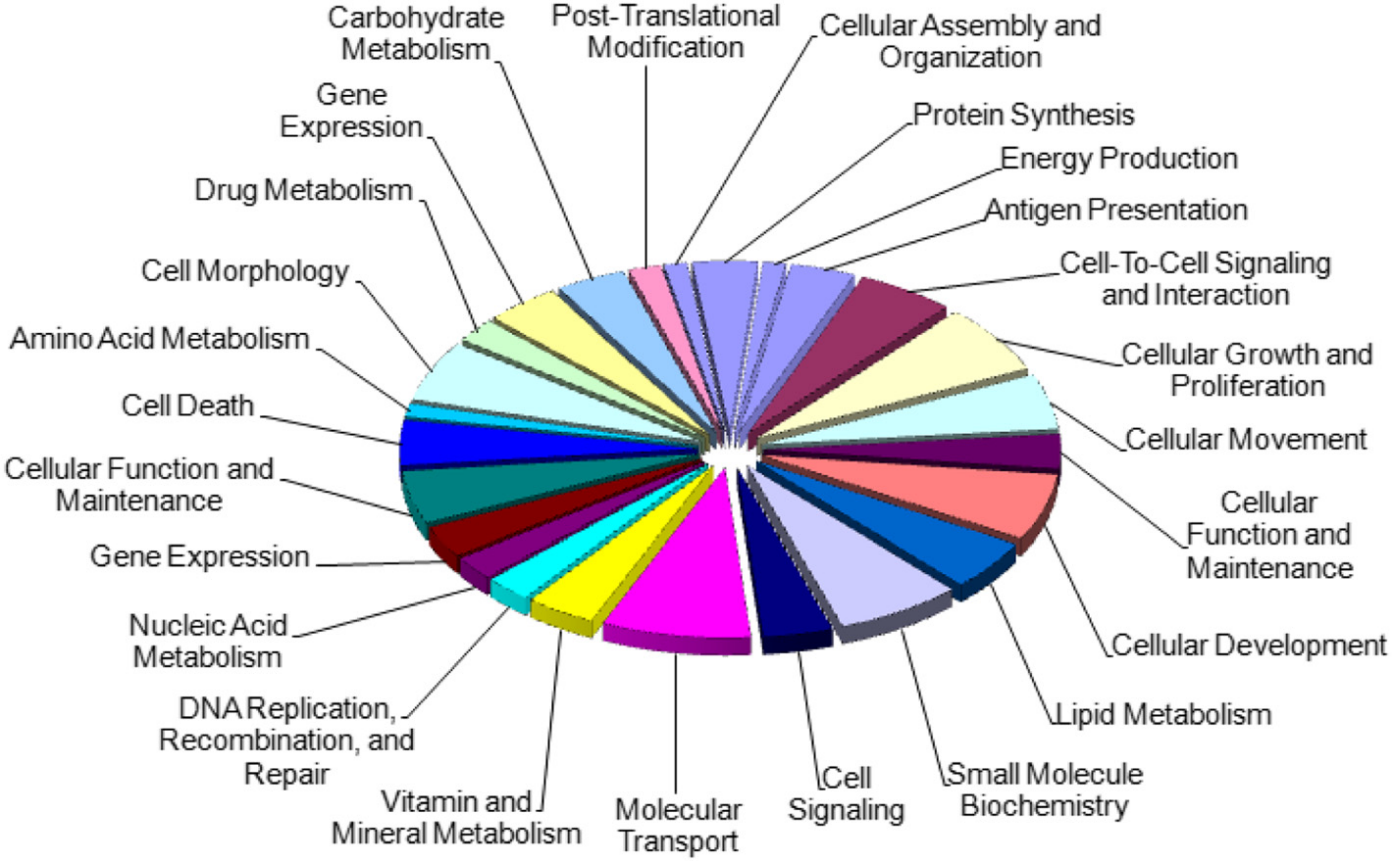
**Molecular and cellular functions associated with genes down-regulated in lymphocytes in response to rapamycin treatment.** The chart shows the number of genes, expressed as a percentage of the total number, involved in each of the molecular and cellular functions deemed significant by IPA Ingenuity.

The up- and down- regulated mTOR genes were shown to be significantly enriched for 25 molecular and cellular functions (Figure 2 and Figure 3), which included cellular growth and proliferation, cell cycle, cell morphology, free radical scavenging, and major metabolic pathways (metabolism of nucleic acids, drugs, vitamins and minerals, lipids, amino acids and carbohydrates – gene lists in Additional file 2: Tables S3 and S4). See the supplementary material for associated diseases, physiological systems and networks (Additional file 2: Tables S5-S10).

In order to identify the Rapamycin-sensitive genes whose regulation may be affected by the ApoE genotype we have compared the list of 1165 mTOR regulated genes (1127 identified by us and 38 identified from the literature) with the list of 154 genes regulated in an ApoE allele-specific manner (Additional file 2: Table S4 of reference [40]). We found only 6 overlapping genes (A2M, B2M, GSN, LRP1, DDIT4 and MAL) in the two datasets. Of these, only one gene (MAL) was found to the differentially regulated in the hippocampus of AD patient relative to controls in the original analysis [40].

### Altered rapamycin-response elements in AD brain

The expression of Rapamycin regulated genes in the brain was assessed using Agilent Custom arrays followed by Significance Analysis of Microarrays (SAM) analysis of the 1165 identified rapamycin-regulated genes only.

The comparison of gene expression in ApoE4 carriers to non-carriers within the same Braak stages (separate two-class unpaired analysis for limbic and neocortical stage AD patients) confirmed that the ApoE genotype had no effect on the expression of the rapamycin-regulated genes in AD patients (data not shown). Therefore all further analysis of limbic stage and neocortical stage patients were carried out on all patients in these groups irrespective of their ApoE genotype.

### Altered rapamycin response elements in mild AD (limbic stage) compared to control

**Figure 4:**
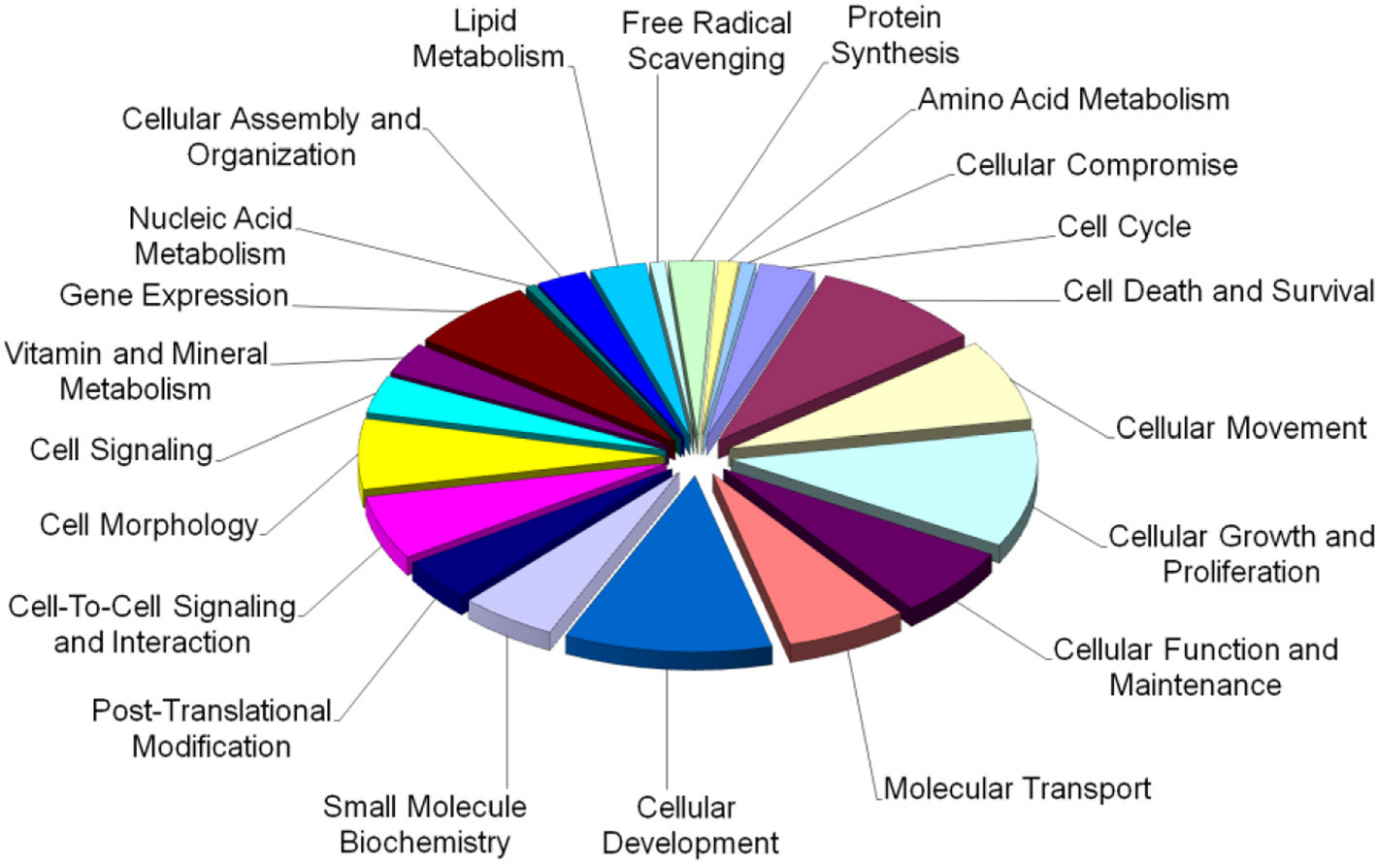
**Molecular and cellular functions associated with mTOR-regulated genes differentially expressed in the frontal lobe of mild AD patients.** Molecular and cellular functions associated with genes differentially expressed in the frontal lobe of mild AD patients (limbic stage) compared to controls (112 genes). The chart shows the number of genes, expressed as a percentage of the total number, involved in each of the molecular and cellular functions deemed significant by IPA Ingenuity.

Of the 1165 known rapamycin-regulated genes, SAM identified 112 (9.6%) that were differentially expressed in brain affected by limbic stage AD compared to control (all up-regulated). These were found to be significantly associated with 20 molecular and cellular functions, such as cell cycle, cell death, cellular movement, molecular transport, small molecule biochemistry, vitamin and mineral metabolism and cellular compromise (Figure 4, gene list in Additional file 2: Table S11). Associated diseases, physiological systems, networks and pathways are outlined in the supplementary material (Additional file 2: Tables S12-S15).

### Altered rapamycin response elements in advanced AD (neocortical stage) compared to control

**Figure 5:**
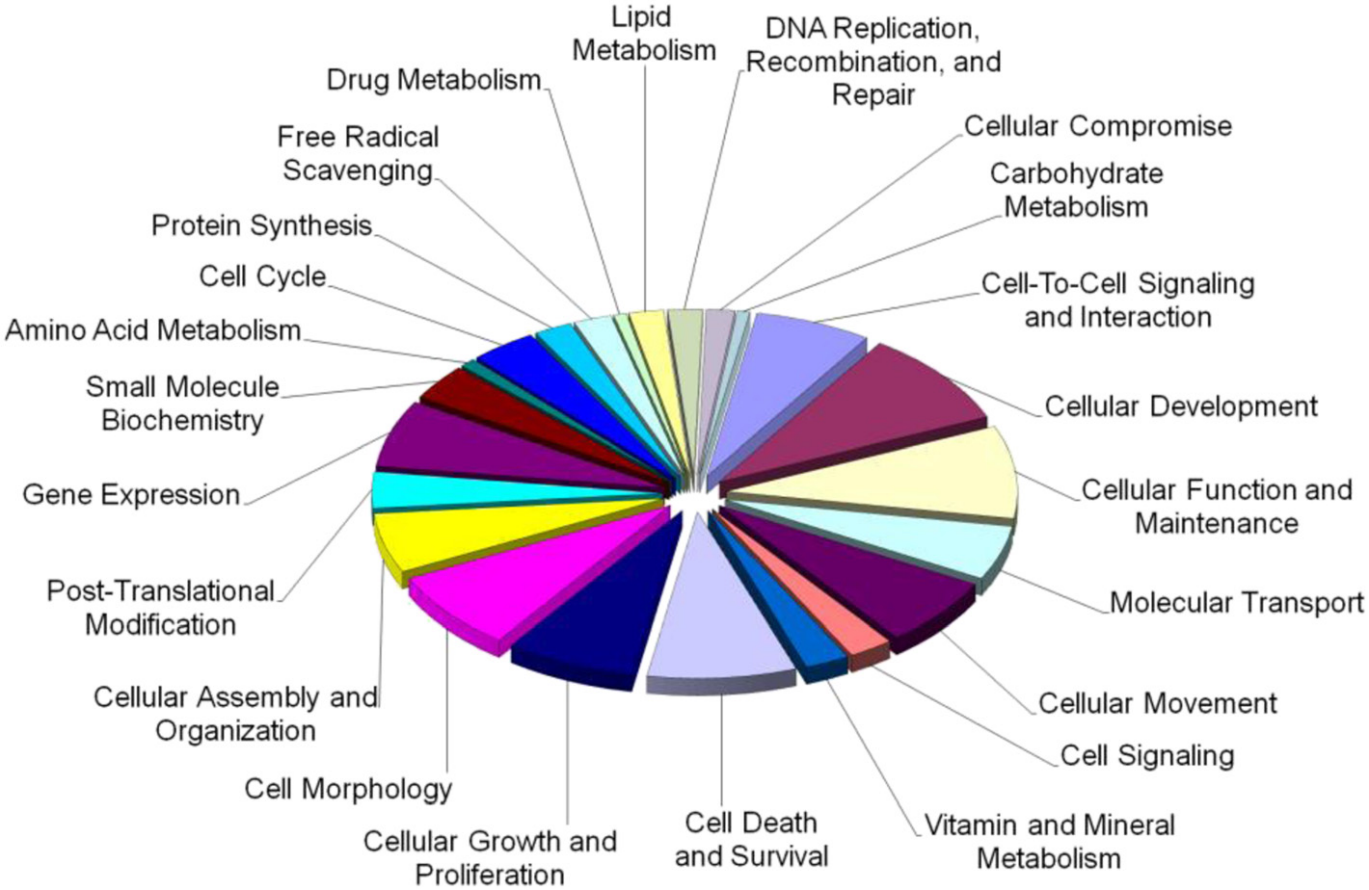
**Molecular and cellular functions associated with mTOR-regulated genes differentially expressed in the frontal lobe of advanced AD patients.** Molecular and cellular functions associated with genes differentially expressed in the frontal lobe of advanced AD patients (neocortical stage) compared to controls (176 genes). The chart shows the number of genes, expressed as a percentage of the total number, involved in each of the molecular and cellular functions deemed significant by IPA Ingenuity.

SAM identified 176 (15.1%) of the 1165 known rapamycin-regulated genes as differentially expressed in brain affected by advanced AD compared to control. The genes were significantly enriched for involvement in 23 molecular and cellular functions, which included cellular function and maintenance, cellular movement, cellular growth and proliferation, cell cycle, cell morphology, and various metabolic pathways (Figure 5 and gene list in Additional file 2: Table S16). Associated diseases and disorders, physiological systems, networks and pathways can be found in the supplementary material (Additional file 2: Tables S17-S20).

Of the 176 genes identified 8 were included in the analysis based on their known association with the mTOR pathway (IPA), while 168 of these genes were identified by us as downstream effectors of mTOR. The differential expression of the majority of these genes (127/168, 76%) was consistent with mTOR inhibition (identical to the direction of change elicited by Rapamycin in lymphocytes, data not shown).

### Altered rapamycin response elements in AD compared to control (multiclass analysis)

**Figure 6:**
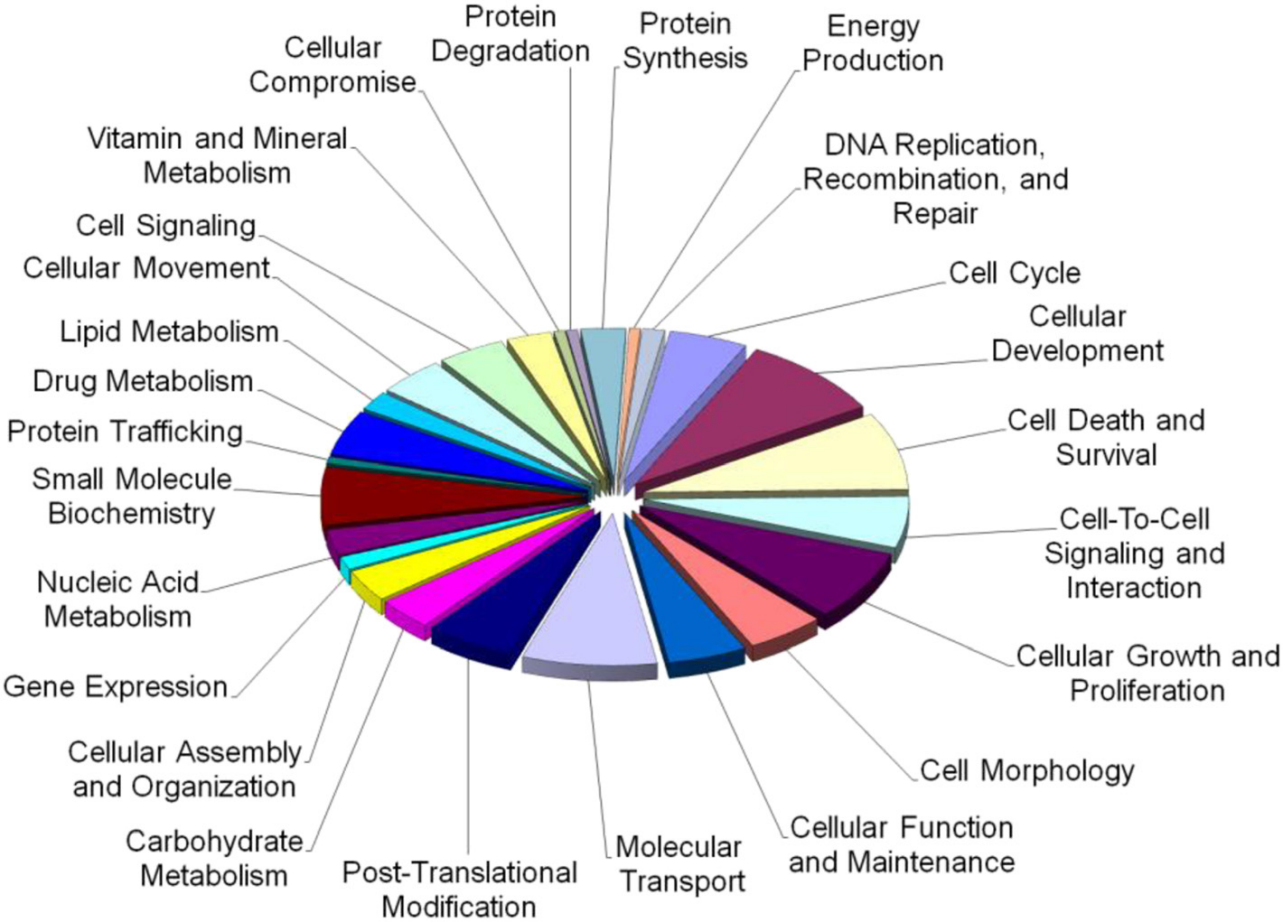
**Molecular and cellular functions associated with genes differentially expressed in AD brain irrespective of severity (55 genes).** The chart shows the number of genes, expressed as a percentage of the total number, involved in each of the molecular and cellular functions deemed significant by IPA Ingenuity.

Of the 1165 rapamycin-regulated genes, multiclass analysis with SAM identified 55 genes that were differentially expressed as a result of AD, irrespective of severity. The differentially regulated genes were significantly enriched for involvement in 24 molecular and cellular functions, which included cellular development, cell death, cell cycle, cell morphology, cellular growth and proliferation, and metabolic pathways (Figure 6 and gene list in Additional file 2: Table S21). Associated diseases, physiological systems, networks and pathways can be found in the supplementary material (Additional file 2: Tables S22-S25).

**Figure 7:**
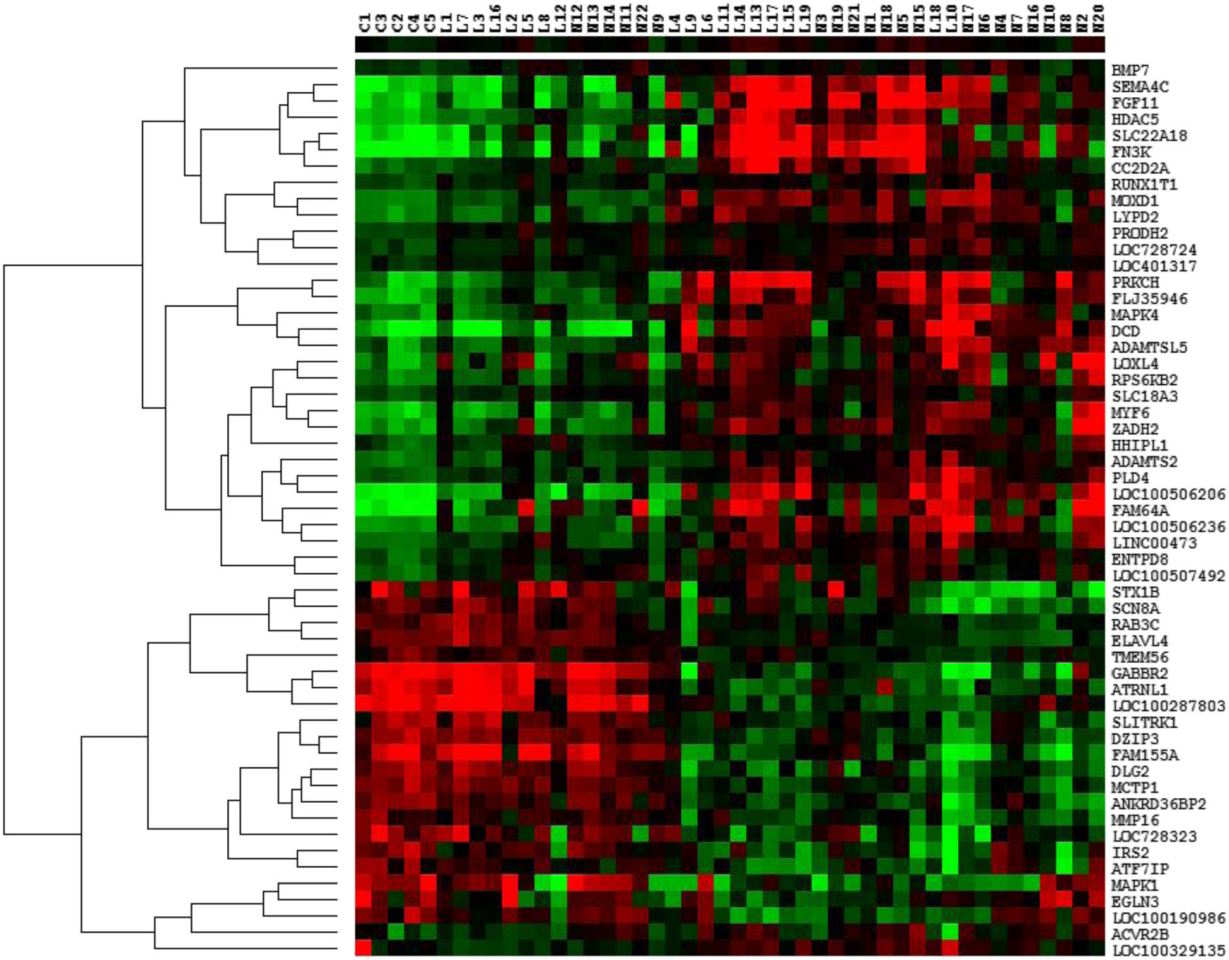
**Differentially expressed mTOR genes in the frontal lobe of AD patients.** Heat map of differentially expressed mTOR genes in the frontal lobe of AD patients compared to control. Differential expression was determined by multiclass analysis in SAM, with the subjects split into three groups according to Braak staging: control, mild AD (limbic stage) and advanced AD (neocortical stage). The cut-off point for differential expression was <10% FDR. The heat map was generated with Cluster and Treeview, with complete hierarchical clustering by gene and array. The expression level of each gene relative to the median expression of the gene across all samples is represented on a red-green colour scale. The colours represent the log ratio of gene expression in an individual patient. Downregulated genes are represented in green (saturated green: log ratios −3.0 and below) while upregulated genes are represented in red (saturated red: log ratios 3.0 and above). Cells with log ratios of 0 (gene expression unchanged) are black. The arrays are identified by the patient codes that are described in Additional file 2: Table S1 (C: Control, L: Limbic stage AD, N: Neocortical stage AD) while the genes are identified by the gene name.

The expression level of the 55 mTOR genes in the frontal lobe were clustered by array and gene, with complete hierarchical clustering, and visualised in a heat map (Figure 7). Thirty-three of these genes were significantly up-regulated in both early and advanced AD compared to control (all these genes were also identified by the earlier two class unpaired analysis). However, this analysis identified, relative to the two-class unpaired analysis, an additional 22 genes that were significantly down-regulated in mild AD compared to control and remained down-regulated in advanced AD. For the majority of subjects, the expression of this set of 55 rapamycin-regulated genes is altered early in the disease process and remains altered in later stages. The arrays (patients) are clustered in a distinctive pattern: with the five control subjects clustered to the left together with 9 limbic stage and 6 neocortical stage patients and the rest of the limbic (10) and neocortical (16) stage patients clustered to the right.

**Figure 8:**
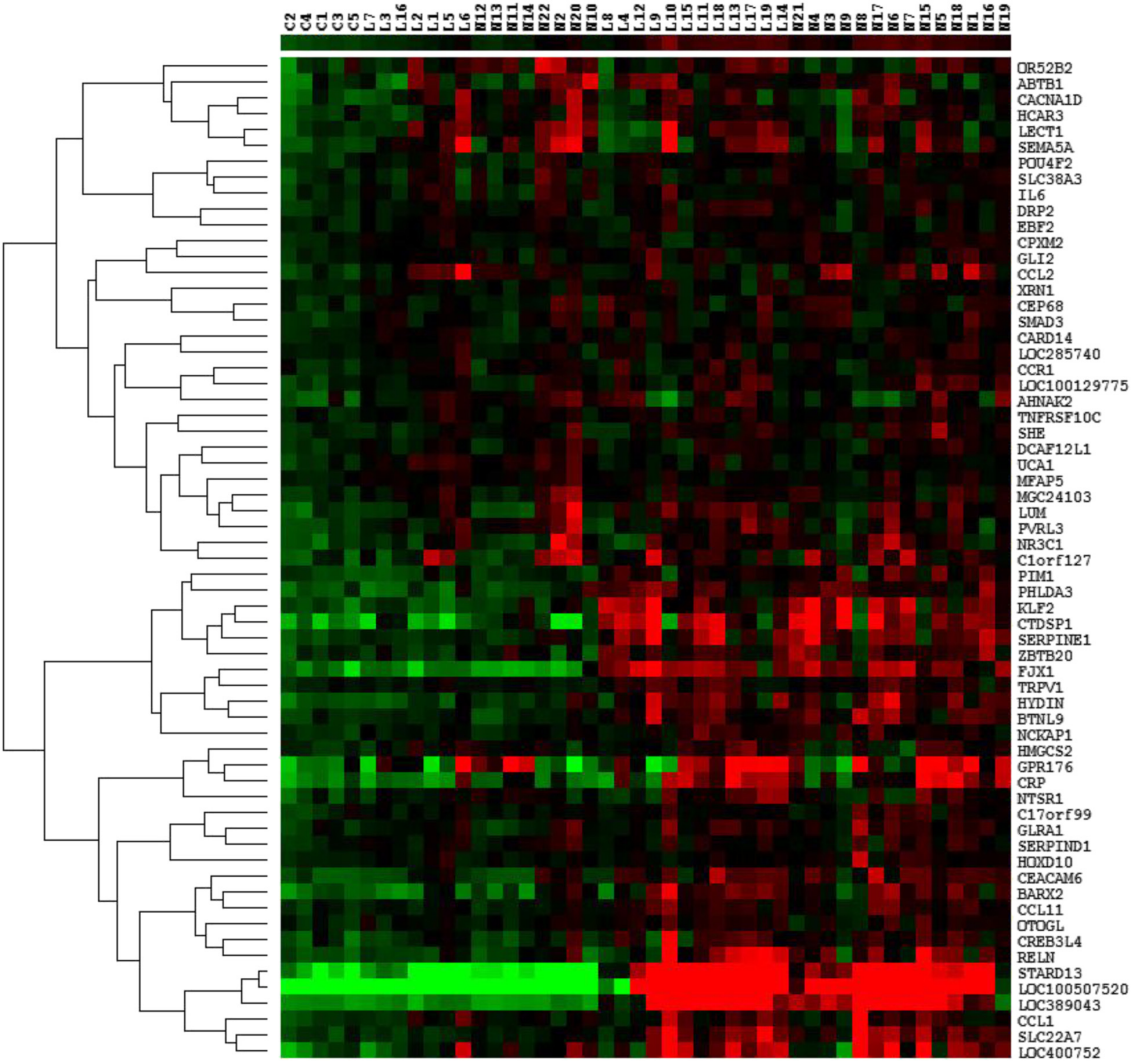
**Rapamycin**-**regulated genes differentially expressed in both mild and advanced AD.** Heat map of the 65 rapamycin-regulated genes that were shown to be differentially expressed in both mild and advanced AD compared to control, in separate two-class unpaired SAM analyses. The cut-off point for differential expression was <10% FDR. The heat map was generated with Cluster and Treeview, with complete hierarchical clustering by gene and array. The expression level of each gene relative to the median expression of the gene across all samples is represented on a red-green colour scale. The colours represent the log ratio of gene expression in an individual patient. Downregulated genes are represented in green (saturated green: log ratios −3.0 and below) while upregulated genes are represented in red (saturated red: log ratios 3.0 and above). Cells with log ratios of 0 (gene expression unchanged) are black. The arrays are identified by the patient codes that are described in Additional file 2: Table S1 (C: Control, L: Limbic stage AD, N: Neocortical stage AD) while the genes are identified by the gene name.

Further comparison of the genes differentially expressed in mild (limbic) and late (neocortical) AD (as determined by separate two-class unpaired analyses in SAM) indicates that a further 63 of the 1165 rapamycin-regulated genes were shown to be differentially regulated in both mild and advanced AD compared to controls. However, the expression of these genes showed a relatively wide variation from patient to patient (see cluster analysis visualised in Figure 8).

**Figure 9:**
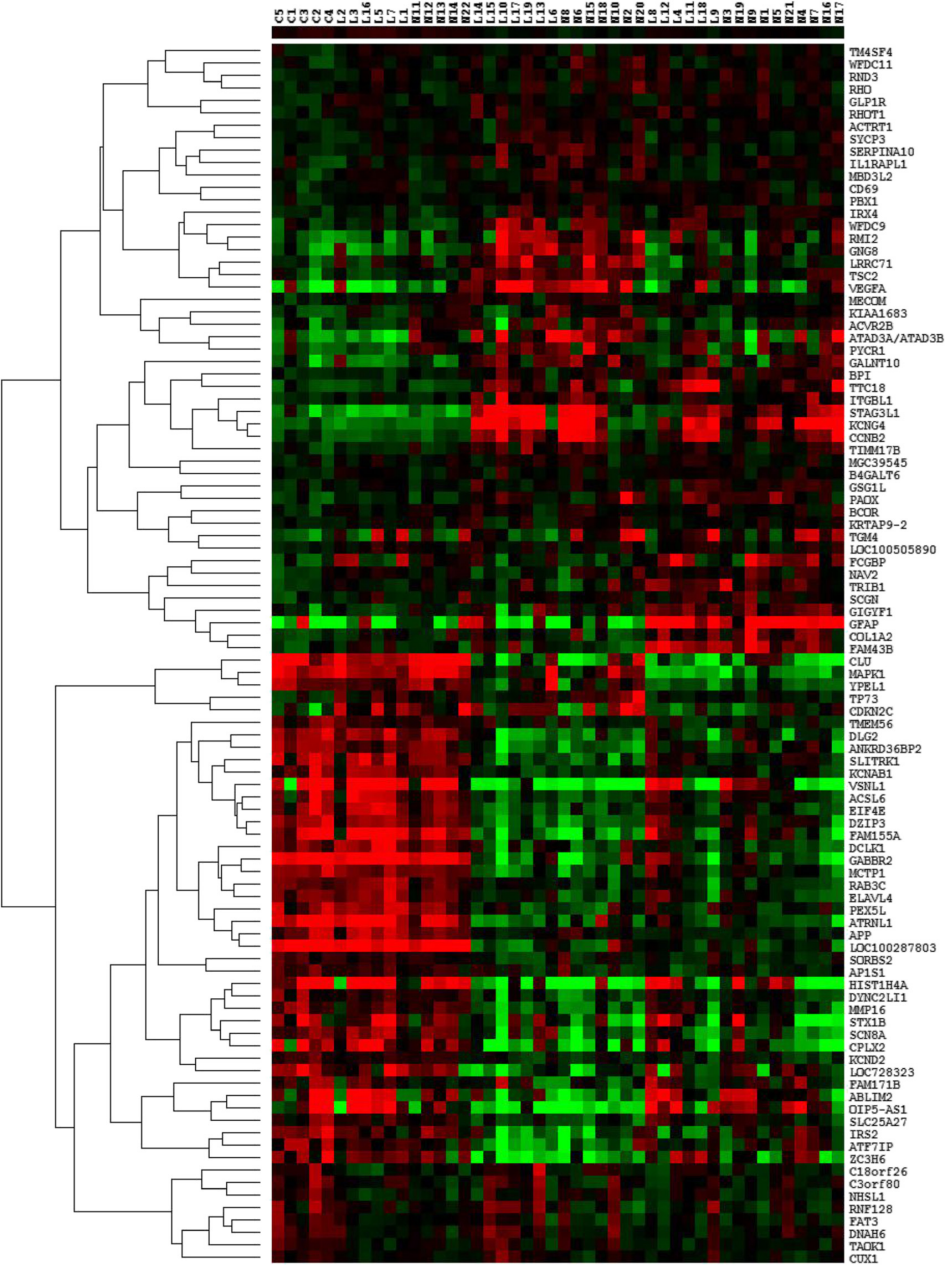
**Differentially expressed mTOR-related genes in either mild or advanced AD.** Heat map visualising the expression level of the 98 genes that were either differentially expressed in mild AD (17 genes) or in advanced AD (81 genes) only. The cut-off point for differential expression was <10% FDR. The heat map was generated with Cluster and Treeview, with complete hierarchical clustering by gene and array. The expression level of each gene relative to the median expression of the gene across all samples is represented on a red-green colour scale. The colours represent the log ratio of gene expression in an individual patient. Downregulated genes are represented in green (saturated green: log ratios −3.0 and below) while upregulated genes are represented in red (saturated red: log ratios 3.0 and above). Cells with log ratios of 0 (gene expression unchanged) are black. The arrays are identified by the patient codes that are described in Additional file 2: Table S1 (C: Control, L: Limbic stage AD, N: Neocortical stage AD) while the genes are identified by the gene name.

We also found that 98 Rapamycin-regulated genes were differentially expressed in either mild AD (17 genes) or advanced AD (81 genes) only (Figure 9) identifying three distinct patient clusters.

### Comparison of differentially expressed transcripts with known AD-related genes

The IPA Ingenuity knowledge database contains information about established gene associations with disease that have been derived from peer-reviewed journals and other high quality sources. Only verified gene and disease associations are included in the database (associations based on genetics and expression).

IPA Ingenuity identifies 519 genes that are known to be involved in AD. This gene list was compared to the list of genes we found to be regulated by rapamycin (1165 genes, ~ 5.82% of the whole genome). Of the 519 AD-related genes we found that 44 (8.48%) were also identified as downstream molecules of mTOR (Additional file 2: Table S26). Additionally while so far 519 genes of the whole genome were found to be associated with AD (2.59% of the whole genome), we have found that 176 of the 1165 mTOR regulated genes (15.1%) have altered expression in AD indicating that the mTOR pathway has a significant involvement in AD (Chi-square=402.354, p < 0.0001).

### Rapamycin response in lymphocytes from Alzheimer’s disease patients

The proliferation rate and population doubling time of lymphocyte cultures with and without rapamycin treatment was calculated from the cell number estimates derived from the LDH assay. We found that the baseline proliferation characteristics (population doubling time, PDT, without Rapamycin treatment) were independent of the age of the patients in our cohort (p=0.162, R2=1.9892%; Additional file 1: Figure S5).

To assess the relative response to Rapamycin in lymphocytes we have calculated the PDT as well as the length of the G1 time in lymphocyte cultures with and without rapamycin for each patient. The relative rapamycin response in lymphocytes (lengthening of the proliferation and G1 time) showed that AD patients have a significantly poorer response to Rapamycin than healthy elderly control subjects (Additional file 1: Figure S6 and Fig. S7). We have also found that the rapamycin response in lymphocytes was not ApoE dependent (Additional file 1: Figure S8 and Figure S9).

**Figure 10:**
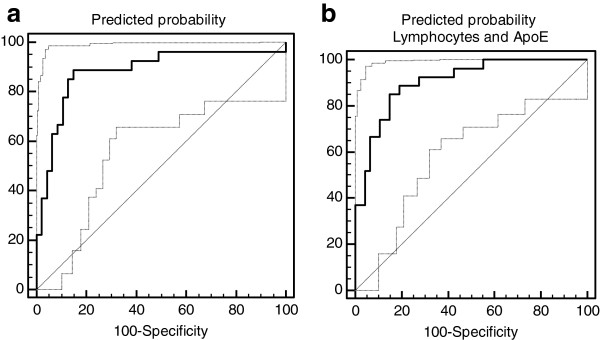
**ROC curve analysis of the performance of the lymphocyte test. a**). Receiver operating characteristic (ROC) curve of the lymphocyte test for differentiation between Alzheimer’s disease patients (probable AD, NINCDS-ARDRA criteria) and healthy control individual. **b**). Receiver operating characteristic (ROC) curve of the combination of the lymphocyte test and ApoE4 status for differentiation between Alzheimer’s disease patients (probable AD, NINCDS-ARDRA criteria) and healthy control individual.

Logistic regression analysis indicates that the combination of the baseline proliferation characteristics and the relative change induced by Rapamycin in the lymphocytes was strongly predictive of the clinical diagnosis in the first 74 patients (27 probable AD and 47 Controls) (P < 0.0001, OR=3.96, and predicted area under the ROC curve: AUC=86.1%, 95% CI: 0.760 to 0.930). ROC analysis of the predicted probability (calculated from the logistic regression) was capable of distinguishing control subjects from AD patients with 88.3% accuracy (AUC=0.88, 95% CI: 0.788 to 0.946; p<0.0001). The specificity and sensitivity of the prediction (at the >0.5 cut-off) is 81.48% and 87.23% respectively (Figure 10a).

The inclusion of the ApoE4 status of the patient in the predictive algorithm increased the overall accuracy of the test (AU ROC: 91.3%, 95% CI: 0.824-0.966, p<0.0001). The sensitivity of the combined test increases to 85% at the expense of the specificity which decreases to 82.9% (Figure 10b).

In the second independent patient group (21 control and 24 AD patients) the application of the test and probability calculations indicates that at the same cut-off value the lymphocyte response to Rapamycin alone identifies 78.6% of the patients accurately with a 87.5% specificity and 76.2% sensitivity. Using ApoE status as a co-predictor the specificity of the prediction increases to 95.83% at the expense of the sensitivity which decreases to 66.67%.

**Figure 11:**
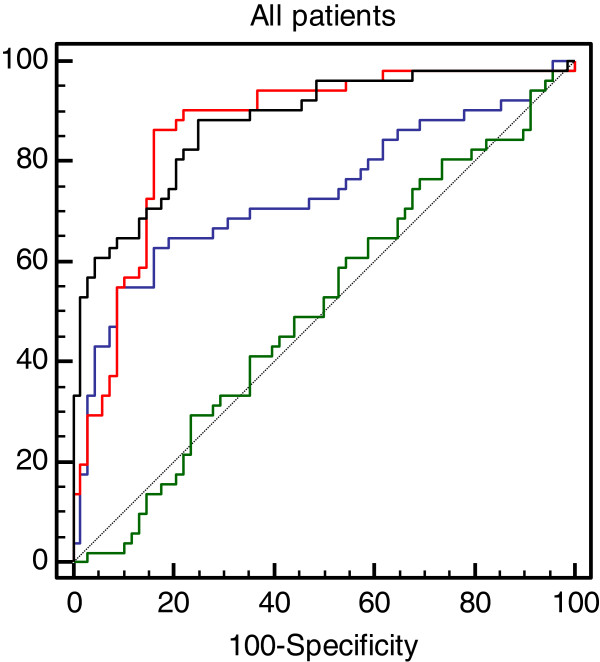
**Comparison of ROC curves.** ROC curve comparison. Green curve: probability of AD due to age; Blue curve: probability of AD based on ApoE4 status; Red line: probability of AD predicted by the Rapamycin response in lymphocytes; Black line: probability of AD as predicted by the combination of the Rapamycin response and ApoE4 status.

ROC analysis indicates that the ApoE4 status, similar to previous studies, is predictive of the patient’s diagnosis (AUC: 0.735; 95% CI: 0.646-0.812). The comparison of ROC curves, however, indicates that the lymphocyte Rapamycin-response provides a significantly better prediction of AD risk (Figure 11).

Overall the lymphocyte test is negative in 85.6% of controls and positive in 86.2% of AD patients (sensitivity and specificity 86% and 82% respectively), indicating that a positive test result is associated with a significantly increased risk of developing AD (OR: 28.80, 95% CI: 10.46 to 79.33; p< 0.0001).

As the age of the subjects included varied between 57 to 95 years, based on published demographic data, the age-dependent probability of AD (pre-test probability) varied from 2.3% to 40.5%. Using only the ApoE4 status as a risk factor the post-test probability of AD has risen to above 80% in 13% (9/67) control subjects, 22.7% (5/27) MCI and 54.9% (28/51) AD patients. Using the lymphocyte test alone the post-test probability has risen to above 80% in 17.9% (12/67) controls, 11% (3/27) MCI and 86.3% (44/51) AD patients. When the ApoE4 status and lymphocyte test were used together the post-test probability of AD has risen above 80% in 28% (19/67) of controls, 29.6% (8/27) MCI and 92.2% (47/51) AD patients.

## Discussion

The functional significance of the mTOR pathway has been revealed by functional studies using Rapamycin as an mTOR inhibitor. However, the downstream effectors of the pathway have not been fully mapped [19]. Our expression analysis in lymphocytes has now identified genes that are downstream effectors of mTOR. The downregulation of genes involved in antigen presentation, cell-to-cell signalling (mainly involved in the immune response), and cellular growth and development are consistent with previous studies into the mechanism of action of Rapamycin [41]. The significantly larger number of genes upregulated by Rapamycin (91%, 1033 of 1127 all Rapamycin-sensitive genes) was somewhat unexpected. The analysis of functional groups indicates that Rapamycin has a significant effect on genes involved in neuronal development and function. Most of the neuronal functions associated with these genes were already known to be affected by Rapamycin [10,19], providing further validation of our results. We have now identified the downstream effectors of the mTOR signalling pathway that are most likely the molecular substrates of the mTOR effect on neuronal plasticity and LTP [10-12]. The strong effect of Rapamycin on these cellular functions also identifies mechanisms responsible for the beneficial effect of the drug in the transgenic model of AD [29].

We have chosen the frontal lobe for differential gene expression studies because of the relatively late involvement of this brain region in the disease process [32]. The samples from limbic stage patients included in the study had minimal tau and amyloid pathology similar to that seen in healthy controls (Additional file 1: Figure S1, Figure S2 and Figure S3). In contrast samples from neocortical stage patients had significantly increased pathology. Thus gene expression changes observed in limbic stage patients (relative to controls) represent the gene expression alterations that precede the development of the AD-type pathology, while changes in the neocortical stage samples represent those that are associated with fully developed AD. While ApoE genotype has a significant effect on gene expression in the brain [40,42], it does not affect the expression of the Rapamycin regulated genes. It is important to note that the gene expression alterations in the brain are not simply a consequence of mTOR activation [24,25]. Due to RNA degradation the arrays from post-mortem samples are likely to give false negative results affecting primarily short transcripts, but with the Agilent microarrays the possibility of false positive results is not greater than for any other array technologies.

The differential expression of a large number of mTOR-dependent genes in both the limbic (112/1165, 9.6%) and neocortical stage AD (176/1165, 15.1 %) indicates that the disruption of these molecular pathways is not merely a consequence of the pathology and implies that the dysregulation of the downstream pathways of mTOR may play an important and early role in the development of AD. It is also interesting that the differentially regulated genes in limbic stage AD (112 genes) and neocortical stage AD (176 genes) were involved in very similar functional categories. For the majority of the functional groups, the number of mTOR genes affected in each category was greater in neocortical stage than in limbic stage AD. Thus it appears that the disruption of these pathways is present before AD pathology develops and the accumulating AD pathology only worsens the dysfunction of the same pathways. The molecular pathways and functions we have found affected in AD are largely the same as those identified earlier in brain regions severely affected by the disease [40,42-45] or those found to be altered in parallel with increased beta amyloid load in the brain [46], with the significant difference that we have now provided evidence that these pathways are affected well before the pathology develops.

**Figure 12:**
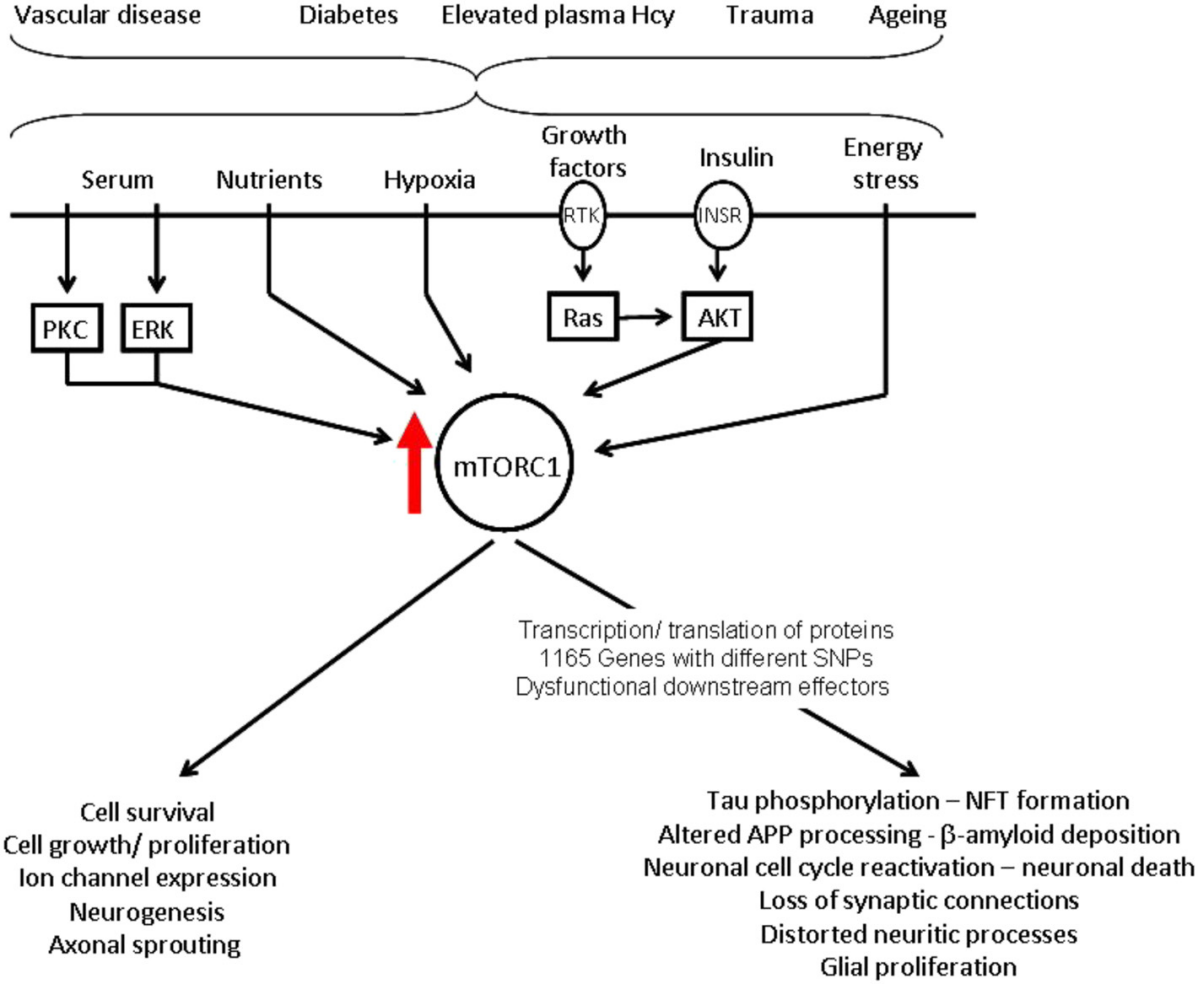
**Causes and consequences of mTOR activation in the brain.** Vascular disease, diabetes, elevated plasma homocysteine levels, trauma and ageing (all risk factor for AD) can elicit the various extracellular signals that can activate mTORC1. Normally, the activation of downstream signals would ensure cell survival and damage repair. However, the dysfunction of the downstream effector pathways, du to genetic variation, will lead to aberrant responses to mTOR activation in the brain, leading to AD-type pathology, neuronal death and glial activation. Abbreviations: Homocysteine (Hcy); Receptor Tyrosine Kinase (RTK); Insulin receptor (INSR); Protein Kinase C (PKC); Extracellular Signal Regulated Kinase (ERK); (Ras); Protein kinase B (AKT); Neurofibrillary tangle (NFT; Amyloid Precursor Protein (APP).

Since Rapamycin significantly affects the expression of several genes associated with cell cycle control, proliferation and cell death in lymphocytes, it is not unexpected that a simple cell proliferation assay combined with flow cytometry is sufficient to assess the Rapamycin-response from these cells in individual patients [20]. The strong association between the outcome of the Rapamycin test in lymphocytes and patient diagnosis indicates that the d1ysfunction of the mTOR downstream signalling pathway is affected systemically in AD patients, rather than just in the brain. Thus, the dysfunction of the mTOR pathway is most likely to be a constitutional characteristic of an individual. As the genes involved in the downstream signalling of mTOR are highly polymorphic in humans, it is likely that this ‘constitutional’ dysfunction of the mTOR pathway in different individuals depends on different SNPs. Depending on the combinations of polymorphisms present in individual patients, differential activation of pathways downstream of mTOR may produce a variety of functional outcomes in the brain in response to mTOR activation. This increases the vulnerability of the brain to the effects of ageing, hypoxia, trauma, elevated homocysteine and diabetes, all known risk factors for AD (summarised in Figure 12). Furthermore, the dysfunction of this pathway could affect the efficacy of neuroprotective therapies in these individuals.

A significant outcome of this study is that, although the pathways affected in early and late AD are identical, the expression of the individual genes that make up these pathways is highly variable in the brain of different patients. This suggests that it is the disruption of specific functional pathways that is associated with AD rather than alterations in a specific gene. This could explain the lack of finding of a single genetic risk factor (other than ApoE) for late onset Alzheimer’s disease [47].

We hypothesise that the assessment of the functional integrity of the downstream signalling cascade of mTOR from lymphocytes allows assessment of the susceptibility of that individual to develop AD rather than provide an assessment of disease state. As such the dysfunction of the mTOR pathway, as measured from lymphocytes, may represent a risk factor for the development of AD that could be exploited diagnostically.

Both the alterations of the mTOR signalling cascade in AD brain and the outcome of the lymphocyte test were independent of the ApoE status of the patients. These findings support the hypothesis that the dysfunction of the mTOR downstream signalling pathway, as a risk factor, is independent of the ApoE genotype.

Further longitudinal studies in MCI patients are necessary to demonstrate the clinical utility of the lymphocyte test in the risk prediction of individuals with memory problems.

## Competing interests

SC Yates, P Hubbard, S Nagy, A Zafar, S Durant, G Wilcock, S Christie have no conflicts of interest. AD Smith was a scientific consultant to CytOx Ltd at the time the study commenced. R Bicknell, MM Esiri and Z Nagy are shareholders of CytOx Ltd (<1.5% each) and were consultants to the company at the time the study commenced. Z Nagy is the named inventor on the patent applications owned by the University of Oxford and University of Birmingham.

## Supplementary Material

Additional file 1: Figure S1 AT8-positive phospho tau in the frontal lobe of the patient included in the study. Click here for file

Additional file 2: Table S1 Demographic data on patients included in the microarray analysis. Click here for file

Additional file 3 Quality standards for the LDH assay. Click here for file
