# Supplementary material for: Dysfunction of the mTOR pathway is a risk factor for Alzheimer’s disease
Source: Acta Neuropathol Commun. 2013 May 8;1:3. doi: 10.1186/2051-5960-1-3 (PMC3776211; doi:10.1186/2051-5960-1-3)
Supplement: Additional file 1: Figure S1 — AT8-positive phospho tau in the frontal lobe of the patient included in the study. [file 2051-5960-1-3-S1.doc]

**Dysfunction of the mTOR pathway is a risk factor for Alzheimer’s disease**

**Sharon C Yates**^1^**, Amen Zafar**^1^**, Paul Hubbard**^1^**, Sheila Nagy**^1^**, Sarah Durant**^2^**, Roy Bicknell**^2^**, Gordon Wilcock**^3^**, Sharon Christie**^3^**, Margaret M Esiri^4^, A David Smith**^5^**, Zsuzsanna Nagy**^1*^

^1^ Neuropharmacology and Neurobiology, College of Medical and Dental Sciences, School of Clinical and Experimental Medicine, University of Birmingham, Birmingham, B15 2TT, UK.

^2^ Institute of Biomedical Research, College of Medical and Dental Sciences, University of Birmingham, Birmingham, B15 2TT, UK.

^3^ OPTIMA, University of Oxford, Level 4, John Radcliffe Hospital, Oxford, OX3 9DU, UK.

^4^ Department of Neuropathology, University of Oxford, Level 1, John Radcliffe Hospital, Oxford, OX3 9DU, UK.

^5^ Department of Pharmacology, University of Oxford, Mansfield Road, Oxford OX1 3QT, UK.

*** Corresponding Author:** Dr. Zsuzsanna Nagy; email: [z.nagy@bham.ac.uk](mailto:z.nagy@bham.ac.uk)

**Online Resource 1. Supplementary Figures.**

### Supplementary Fig.1 AT8-positive phospho tau in the frontal lobe of the patient included in the study

### The x-axis represents the severity of AD as defined by Braak staging: E = entorhinal stage, L = limbic stage, N = neocortical stage. The y-axis represents the amount of AT8-positive phospho tau in the frontal lobe of the patients included in the study as determined by ELISA (arbitrary units). The top of the bars represent the mean and the error bars the standard error of the mean.

### Supplementary Fig.2 DC11-positive tau in the frontal lobe of the patient included in the study

### The x-axis represents the severity of AD as defined by Braak staging: E = entorhinal stage, L = limbic stage, N = neocortical stage. The y-axis represents the amount of DC11-positive phospho tau in the frontal lobe of the patients included in the study as determined by ELISA (arbitrary units). The top of the bars represent the mean and the error bars the standard error of the mean.

### Supplementary Fig.3 Beta-amyloid in the frontal lobe of the patient included in the study

### The x-axis represents the severity of AD as defined by Braak staging: E = entorhinal stage, L = limbic stage, N = neocortical stage. The y-axis represents the amount of beta-amyloid in the frontal lobe of the patients included in the study as determined by ELISA (arbitrary units). The top of the bars represent the mean and the error bars the standard error of the mean.

**Supplementary Fig.4** Validation of microarray result by Q-PCR for six differentially expressed genes

The y-axis represents the relative expression of the gene-of-interest. The top of the bars represent the mean and the error bars the standard error of the mean. The blue and red bars represent the results obtained for the control and advanced AD subjects respectively.

| Fig.2A. Comparison of the expression of six genes, as determined by microarray, in the brain of control and advanced AD patients | Fig.2B. Comparison of the expression of the same six genes, as determined by Q-PCR, in the brain of the same control and advanced AD patients |
| --- | --- |
| 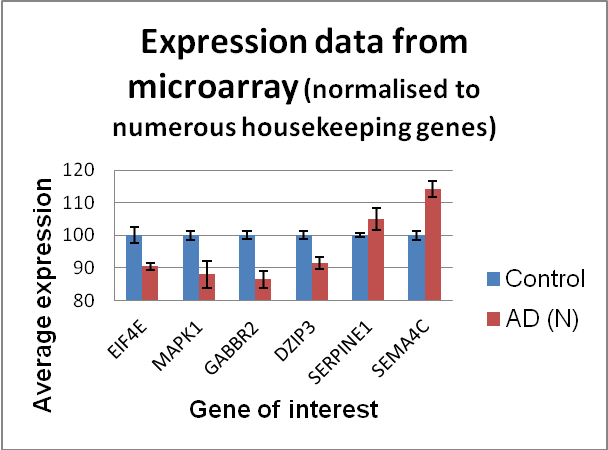 | **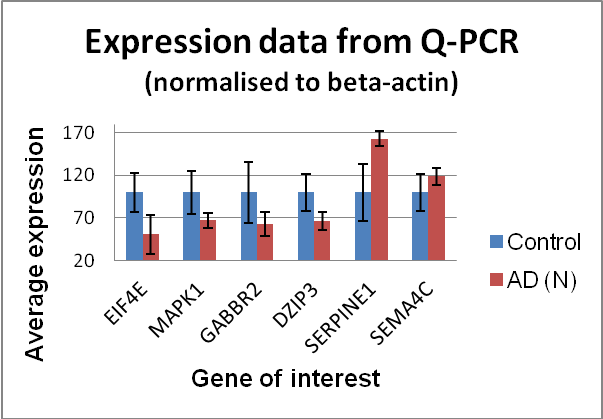** |

### Supplementary Fig.5 Relationship between age of the patient and baseline proliferation characteristics of lymphocytes

Regression analysis, relationship between age of the patient and baseline population doubling time (PDT) of lymphocytes. The x-axis represents the age of the patients (in years). The y-axis represents the PDT of the lymphocyte cultures (in hours). Continous line: regression line, dotted lines 95% confidence intervals.

### Supplementary Fig.6 The effect of Rapamycin on cell division in lymphocytes. Comparison of patient groups

The x-axis represents the different patient groups included in the study: Control = Healthy elderly subjects, MCI = patients with mild cognitive impairment, Prob. AD = AD patients who fulfill the criteria fro probable AD by the NINCDS-ARDRA criteria. The y-axis represents the ratio of cell divisions in Rapamycin treated lymphocyte cultures (n’) and the number of cell divisions in the lymphocyte cultures without Rapamycin (n). The top of the bars represent the mean and the error bars the standard error of the mean.

### Supplementary Fig.7 The effect of Rapamycin on the length of the G1 time in lymphocytes. Comparison of patient groups

The x-axis represents the different patient groups included in the study: Control = Healthy elderly subjects, MCI = patients with mild cognitive impairment, Prob. AD = AD patients who fulfill the criteria fro probable AD by the NINCDS-ARDRA criteria. The y-axis represents the ratio of the G1 time in Rapamycin treaten lymphocyte cultures (TG1_Rapa) and the G1 time in the lymphocyte cultures without Rapamycin (TG1_Control). The top of the bars represent the mean and the error bars the standard error of the mean.

### Supplementary Fig.8 The effect of Rapamycin on cell division in lymphocytes. Comparison of patient with different ApoE genotypes

The x-axis represents the groups defined by the ApoE4 status of the patients. 0 = Subjects with no ApoE ε4 allele, 1 = patients with one ApoE ε4 allele, 2 = patients with 2 ApoE ε4 alleles. The y-axis represents the ratio of cell divisions in Rapamycin treated lymphocyte cultures (n’) and the number of cell divisions in the lymphocyte cultures without Rapamycin (n). The top of the bars represent the mean and the error bars the standard error of the mean.

### Supplementary Fig.9 The effect of Rapamycin on the length of the G1 time in lymphocytes. Comparison of patient with different ApoE genotypes

The x-axis represents the groups defined by the ApoE4 status of the patients. 0 = Subjects with no ApoE ε4 allele, 1 = patients with one ApoE ε4 allele, 2 = patients with 2 ApoE ε4 alleles. The y-axis represents the ratio of the G1 time in Rapamycin treated lymphocyte cultures (TG1_Rapa) and the G1 time in the lymphocyte cultures without Rapamycin (TG1_Control). The top of the bars represent the mean and the error bars the the standard error of the mean.
