# Supplementary material for: Dysfunction of the mTOR pathway is a risk factor for Alzheimer’s disease
Source: Acta Neuropathol Commun. 2013 May 8;1:3. doi: 10.1186/2051-5960-1-3 (PMC3776211; doi:10.1186/2051-5960-1-3)
Supplement: Additional file 2: Table S1 — Demographic data on patients included in the microarray analysis. [file 2051-5960-1-3-S2.doc]

**Dysfunction of the mTOR pathway is a risk factor for Alzheimer’s disease**

**Sharon C Yates**^1^**, Amen Zafar**^1^**, Paul Hubbard**^1^**, Sheila Nagy**^1^**, Sarah Durant**^2^**, Roy Bicknell**^2^**, Gordon Wilcock**^3^**, Sharon Christie**^3^**, Margaret M Esiri^4^, A David Smith**^5^**, Zsuzsanna Nagy**^1*^

^1^ Neuropharmacology and Neurobiology, College of Medical and Dental Sciences, School of Clinical and Experimental Medicine, University of Birmingham, Birmingham, B15 2TT, UK.

^2^ Institute of Biomedical Research, College of Medical and Dental Sciences, University of Birmingham, Birmingham, B15 2TT, UK.

^3^ OPTIMA, University of Oxford, Level 4, John Radcliffe Hospital, Oxford, OX3 9DU, UK.

^4^ Department of Neuropathology, University of Oxford, Level 1, John Radcliffe Hospital, Oxford, OX3 9DU, UK.

^5^ Department of Pharmacology, University of Oxford, Mansfield Road, Oxford OX1 3QT, UK.

*** Corresponding Author:** Dr. Zsuzsanna Nagy; email: [z.nagy@bham.ac.uk](mailto:z.nagy@bham.ac.uk)

# Online Resource 3. Supplementary tables.

### Supplementary Table 1. Demographic data on patients included in the microarray analysis.

| ***Patient code*** | ***Gender*** | ***Age at death*** | ***Diagnosis*** | ***Braak Stage*** | ***ApoE genotype*** |
| --- | --- | --- | --- | --- | --- |
| C1 | f | 88 | Control | O | E3E3 |
| C2 | m | 87 | Control | I | E3E3 |
| C3 | f | 90 | Control | I | E3E3 |
| C4 | f | 82 | Control | I | E3E3 |
| C5 | m | 82 | Control | I | E3E3 |
| L1 | m | 100 | Limbic Stage AD | III-IV | E3E3 |
| L2 | f | 91 | Limbic Stage AD | III-IV | E3E3 |
| L3 | m | 87 | Limbic Stage AD | III-IV | E3E3 |
| L4 | m | 89 | Limbic Stage AD | III-IV | E3E3 |
| L5 | m | 83 | Limbic Stage AD | III-IV | E3E3 |
| L6 | f | 86 | Limbic Stage AD | III-IV | E3E3 |
| L7 | f | 78 | Limbic Stage AD | III-IV | E3E3 |
| L8 | f | 100 | Limbic Stage AD | III-IV | E3E3 |
| L9 | f | 86 | Limbic Stage AD | III-IV | E3E3 |
| L10 | f | 85 | Limbic Stage AD | III-IV | E3E3 |
| L11 | m | 85 | Limbic Stage AD | III-IV | E3E3 |
| L12 | m | 83 | Limbic Stage AD | III-IV | E3E3 |
| L13 | m | 92 | Limbic Stage AD | III-IV | E3E3 |
| L14 | m | 84 | Limbic Stage AD | III-IV | E3E3 |
| L15 | f | 85 | Limbic Stage AD | III-IV | E3E4 |
| L16 | f | 87 | Limbic Stage AD | III-IV | E3E4 |
| L17 | f | 80 | Limbic Stage AD | III-IV | E3E4 |
| L18 | m | 85 | Limbic Stage AD | III-IV | E3E4 |
| L19 | m | 80 | Limbic Stage AD | III-IV | E3E4 |
| N1 | f | 58 | Neocortical Stage AD | V-VI | E3E3 |
| N2 | f | 81 | Neocortical Stage AD | V-VI | E3E3 |
| N3 | m | 77 | Neocortical Stage AD | V-VI | E3E3 |
| N4 | m | 85 | Neocortical Stage AD | V-VI | E3E3 |
| N5 | m | 71 | Neocortical Stage AD | V-VI | E3E4 |
| N6 | m | 70 | Neocortical Stage AD | V-VI | E3E4 |
| N7 | m | 79 | Neocortical Stage AD | V-VI | E3E4 |
| N8 | f | 78 | Neocortical Stage AD | V-VI | E3E4 |
| N9 | m | 66 | Neocortical Stage AD | V-VI | E3E4 |
| N10 | f | 77 | Neocortical Stage AD | V-VI | E3E4 |
| N11 | m | 88 | Neocortical Stage AD | V-VI | E3E4 |
| N12 | m | 75 | Neocortical Stage AD | V-VI | E3E4 |
| N13 | f | 90 | Neocortical Stage AD | V-VI | E3E4 |
| N14 | f | 75 | Neocortical Stage AD | V-VI | E3E4 |
| N15 | m | 82 | Neocortical Stage AD | V-VI | E3E4 |
| N16 | f | 70 | Neocortical Stage AD | V-VI | E3E4 |
| N17 | m | 77 | Neocortical Stage AD | V-VI | E3E4 |
| N18 | F | 65 | Neocortical Stage AD | V-VI | E3E4 |
| N19 | m | 62 | Neocortical Stage AD | V-VI | E3E4 |
| N20 | f | 81 | Neocortical Stage AD | V-VI | E3E4 |
| N21 | m | 62 | Neocortical Stage AD | V-VI | E3E4 |
| **N22** | f | 68 | Neocortical Stage AD | V-VI | E3E4 |

### Supplementary Table 2. Q-PCR: Probe, primer sequences, and annealing temperature corresponding to each gene-of-interest

| **Gene** | **Roche**  **Probe** | **Forward primer** | **Backward primer** | **Optimal annealing temp (ºC)** |
| --- | --- | --- | --- | --- |
| Beta actin | **24** | 5’-TCAGCTGTGG  GGTCCTGT-3’ | 5’-GAAGGGGACA  GGCAGTGAG-3’ | 62 |
| EIF4E  (Variant 1/ 2) | **35** | 5’-gatggcgac  tgtcgaacc-3’ | 5’-tgggttagcaa  cctcctgat-3’ | 60 |
| EIF4E  (Variant 3) | **35** | 5’-gtgtagcgca  cactttctgg-3’ | 5’-tgggttagca  acctcctgat-3’ | 60 |
| MAPK1  (Variant 1/ 2) | **62** | 5’-ccgtgacct  caagccttc-3’ | 5’-gccaggcca  aagtcacag-3’ | 58 |
| GABBR2 | **3** | 5’-gcgaaggac  agtggagaagt-3’ | 5’-gagagggcg  gatggagata-3’ | 62 |
| SEMA4C | **14** | 5’-ttgtgccgc  gtaagacagt-3’ | 5’-cagcgtca  gtgtcaggaagt-3’ | 60 |
| DZIP3 | **39** | 5’-tgcccaagat  ctgatacaagg-3’ | 5’-ctccaacac  accaccgtaca-3’ | 60 |
| SERPINE1 | **80** | 5’-ctcctggttc  tgcccaagt-3’ | 5’-caggttct  ctaggggcttcc-3’ | 58 |

### Supplementary Table 3. Molecular and cellular functions significantly associated with the genes that were upregulated in lymphocytes in response to rapamycin treatment.

| **Category** | **Functional subgroups** | **p-value** | **No. of molecules** | **Molecules** |
| --- | --- | --- | --- | --- |
| Cellular Development |  | 3.86E-10-7.04E-04 | 208 |  |
|  | differentiation of neurons | 3.86E-10 | 48 | ASPM, BCL2, BMP7, CDKN1B, CDKN2C, CDON, CORIN, CTDSP1, DLX2, EBF2, EBF3, EGF, ELAVL3, ELAVL4, EPO, ERBB3, EYA1, FGFR1, FN1, GCM2, GFRA1, GLI2, GSN, HGF, HOXA2, HOXC8, IGF2, IL1RAPL1, JUN, LEP, LIFR, MAPT, NEUROD4, NR4A2, OLIG2, POU4F2, PTPRZ1, REST, RET, RIT2, SALL3, SOCS2, SOX11, TCF12, TGFB3, THRA, TLX1, TP73 |
|  | development of neurons | 1.46E-04 | 18 | CDKN1C, EBF2, EBF3, ERBB3, ERBB4, FGFR1, GABRA5, GFRA1, GFRA2, GLI2, GSN, IL1RAPL1, NEUROD4, NR4A2, OLIG2, PLAT, TLX1, TP73 |
|  | proliferation of neuronal cells | 1.55E-04 | 19 | ANKRD6, B2M, CDKN1B, CDKN2C, CDON, EGF, EPO, F2, HGF, HHIP, LAMA1, LEP, MAPT, MXI1, NEUROD4, OTX1, PAX3, RAG1, SEMA4C |
|  | neuritogenesis | 5.82E-04 | 36 | BAIAP2, BCL2, BMP7, BSN, CD44, DCC, DCLK1, DLX2, EPO, ERBB4, FGFR1, FNBP1, GDA, GFRA2, HGF, IFNA16, IL1B, JUN, LAMA1, LEP, LIFR, LOX, MAPT, MBP, OPRM1, PLAT, POU4F2, PRICKLE2, PTPRZ1, RGNEF, SLC18A3, SLITRK1, SLITRK5, STMN1, STMN3, ULK2 |
| Cellular Movement |  | 1.03E-09-7.6E-04 | 152 |  |
|  | migration of neurons | 1.83E-06 | 27 | ANGPT2, ASPM, CDKN1B, CDKN2C, DCC, DCLK1, DLX2, EBF2, EBF3, EGF, ERBB3, FGFR1, FN1, GFRA1, GSN, HGF, LAMA1, MARK1, NEUROD4, NR2F2, NR4A2, PLAT, PTPRZ1, RET, SEMA4C, SEMA6A, STMN1 |
| Cellular Growth and Proliferation |  | 1.78E-07-7.04E-04 | 165 |  |
| Cell-To-Cell Signaling and Interaction |  | 2.73E-07-7.04E-04 | 146 |  |
|  | action potential of neurons | 5.68E-04 | 13 | CPLX2, DLG2, GRIA2, GRIA3, GRIK2, IL1B, KCNAB1, KCNC2, OPRM1, PDC, SCN8A, SHANK3, TRPV1 |
| Cellular Function and Maintenance |  | 1.35E-06-7.59E-04 | 150 |  |
|  | hyperpolarization of neurons | 2.79E-04 | 5 | ADIPOQ, IL1B, LEP, OPRM1, PMCH |
|  | microtubule dynamics | 1.08E-04 | 73 | A2M, ADIPOQ, ANGPT2, ARHGEF4, BAIAP2, BCL2, BMP7, BSN, CD44, CDH1, CDH13, CDKN1B, CSF1R, CYR61, DCC, DCLK1, DLX2, DST, EGF , EPO, ERBB4, ESR1, EYA1, F2, FGF7, FGFR1, FGR, FN1, FNBP1, GAB1, GDA, GFRA2, GSN, HGF, HYDIN, IFNA16, IL1B, ITGB3, JUN, KLF2, LAMA1, LEP, LIFR, LOX, LRP1 , MAPT, MARK1, MBP, MMP12, NFIB, OPRM1, PARVA, PKHD1, PKP1, PLAT, POU4F2, PRICKLE2, PTPRZ1, RET, RGNEF, RIT2, SALL3, SERPINE1, SLC18A3, SLITRK1, SLITRK5, STMN1, STMN3, TAOK1, TGFB3, TLR4, ULK2, WASF2 |
|  | neuritogenesis | 5.82E-04 | 36 | BAIAP2, BCL2, BMP7, BSN, CD44 , DCC, DCLK1, DLX2, EPO, ERBB4, FGFR1, FNBP1, GDA, GFRA2, HGF, IFNA16, IL1B, JUN, LAMA1, LEP, LIFR, LOX, MAPT, MBP, OPRM1, PLAT, POU4F2, PRICKLE2, PTPRZ1, RGNEF, SLC18A3, SLITRK1, SLITRK5, STMN1, STMN3, ULK2 |
| Cell Cycle |  | 2.37E-06-7.17E-04 | 44 |  |
| Cell Morphology |  | 2.8E-06-7.04E-04 | 146 |  |
|  | morphology of neuroglia | 4.46E-06 | 17 | ABCD2, AQP4, CDKN1B, CDKN1C, CRB1, ERBB3, ESR1, GFAP, IL18 , IL1B, LEP, MBP, NEUROD4, NFIB, PTPRC, PTPRZ1, SPP1 |
|  | outgrowth of neurites | 5.68E-04 | 31 | A2M, BAIAP2, BCL2, DCC, EGF , ELAVL4, ERBB4, F2, FGF7, FGFR1, FN1, GAB1, GFAP, GFRA1, GFRA2, HGF, ITGB3, JUN, LAMA1, LIFR, MAPT, NPTX1, PLAT, POU4F2, PTPRZ1, RET, RIT2, SEMA5A, SOCS2, TNFRSF19, TP73 |
|  | neuritogenesis | 5.82E-04 | 36 | BAIAP2, BCL2, BMP7, BSN, CD44 , DCC, DCLK1, DLX2, EPO, ERBB4, FGFR1, FNBP1, GDA, GFRA2, HGF, IFNA16, IL1B, JUN, LAMA1, LEP, LIFR, LOX, MAPT, MBP, OPRM1, PLAT, POU4F2, PRICKLE2, PTPRZ1, RGNEF, SLC18A3, SLITRK1, SLITRK5, STMN1, STMN3, ULK2 |
| DNA Replication, Recombination, and Repair |  | 3.47E-06-3.47E-06 | 40 |  |
| Cell Death |  | 1.13E-05-7.04E-04 | 127 |  |
| Molecular Transport |  | 1.53E-05-7.59E-04 | 157 |  |
| Nucleic Acid Metabolism |  | 1.53E-05-3.96E-04 | 33 |  |
| Small Molecule Biochemistry |  | 1.53E-05-7.17E-04 | 105 |  |
| Cellular Assembly and Organization |  | 2.12E-05-7.05E-04 | 104 |  |
|  | outgrowth of neurites | 5.68E-04 | 31 | A2M, BAIAP2, BCL2, DCC, EGF , ELAVL4, ERBB4, F2, FGF7, FGFR1, FN1, GAB1, GFAP, GFRA1, GFRA2, HGF, ITGB3, JUN, LAMA1, LIFR, MAPT, NPTX1, PLAT, POU4F2, PTPRZ1, RET, RIT2, SEMA5A, SOCS2, TNFRSF19, TP73 |
|  | neuritogenesis | 5.82E-04 | 36 | BAIAP2, BCL2, BMP7, BSN, CD44 , DCC, DCLK1, DLX2, EPO, ERBB4, FGFR1, FNBP1, GDA, GFRA2, HGF, IFNA16, IL1B, JUN, LAMA1, LEP, LIFR, LOX, MAPT, MBP, OPRM1, PLAT, POU4F2, PRICKLE2, PTPRZ1, RGNEF, SLC18A3, SLITRK1, SLITRK5, STMN1, STMN3, ULK2 |
| Lipid Metabolism |  | 2.72E-05-7.17E-04 | 74 |  |
| Drug Metabolism |  | 2.82E-05-7.17E-04 | 21 |  |
| Vitamin and Mineral Metabolism |  | 2.82E-05-7.59E-04 | 47 |  |
| Cell Signaling |  | 6E-05-7.59E-04 | 61 |  |
| Energy Production |  | 7.51E-05-7.51E-05 | 3 |  |
| Cellular Compromise |  | 8.83E-05-2.92E-04 | 10 |  |
| Gene Expression |  | 9.46E-05-7.17E-04 | 143 |  |
| Antigen Presentation |  | 1.11E-04-6.1E-04 | 25 |  |
| Carbohydrate Metabolism |  | 1.31E-04-3.33E-04 | 53 |  |
| Protein Synthesis |  | 2.12E-04-2.12E-04 | 43 |  |
| Free Radical Scavenging |  | 3.86E-04-3.86E-04 | 16 |  |
| Amino Acid Metabolism |  | 7.04E-04-7.04E-04 | 6 |  |
| Cellular Development |  | 3.86E-10-7.04E-04 | 208 |  |
| Cellular Movement |  | 1.03E-09-7.6E-04 | 152 |  |
| Cellular Growth and Proliferation |  | 1.78E-07-7.04E-04 | 165 |  |
| Cell-To-Cell Signaling and Interaction |  | 2.73E-07-7.04E-04 | 146 |  |
| Cellular Function and Maintenance |  | 1.35E-06-7.59E-04 | 150 |  |
| Cell Cycle |  | 2.37E-06-7.17E-04 | 44 |  |
| Cell Morphology |  | 2.8E-06-7.04E-04 | 146 |  |
| DNA Replication, Recombination, and Repair |  | 3.47E-06-3.47E-06 | 40 |  |
| Cell Death |  | 1.13E-05-7.04E-04 | 127 |  |
| Molecular Transport |  | 1.53E-05-7.59E-04 | 157 |  |
| Nucleic Acid Metabolism |  | 1.53E-05-3.96E-04 | 33 |  |
| Small Molecule Biochemistry |  | 1.53E-05-7.17E-04 | 105 |  |
| Cellular Assembly and Organization |  | 2.12E-05-7.05E-04 | 104 |  |
| Lipid Metabolism |  | 2.72E-05-7.17E-04 | 74 |  |
| Drug Metabolism |  | 2.82E-05-7.17E-04 | 21 |  |
| Vitamin and Mineral Metabolism |  | 2.82E-05-7.59E-04 | 47 |  |
| Cell Signaling |  | 6E-05-7.59E-04 | 61 |  |
| Energy Production |  | 7.51E-05-7.51E-05 | 3 |  |
| Cellular Compromise |  | 8.83E-05-2.92E-04 | 10 |  |
| Gene Expression |  | 9.46E-05-7.17E-04 | 143 |  |
| Antigen Presentation |  | 1.11E-04-6.1E-04 | 25 |  |
| Carbohydrate Metabolism |  | 1.31E-04-3.33E-04 | 53 |  |
| Protein Synthesis |  | 2.12E-04-2.12E-04 | 43 |  |
| Free Radical Scavenging |  | 3.86E-04-3.86E-04 | 16 |  |
| Amino Acid Metabolism |  | 7.04E-04-7.04E-04 | 6 |  |

### Supplementary Table 4. Molecular and cellular functions significantly associated with the genes that were down-regulated in lymphocytes in response to rapamycin treatment.

| **Category** | **p-value** | **No. of genes** | **Molecules** |
| --- | --- | --- | --- |
| Antigen Presentation | 2.35E-09-6.08E-04 | 14 | IL3, THBS1, IL10, FGF2, TAC1, IL6, AQP9, PRKG1, CCL2, IL2, HCK, CCL8, PTGS2, AGT |
| Cell-To-Cell Signaling and Interaction | 2.35E-09-6.08E-04 | 21 | IL3, SLC4A2, RRAD, IL10, THBS1, FGF2, GRIA1, TAC1, IL6, GABBR2, PRKG1, AR, CCL2, IL2, FGF18, HCK, EGLN3, CCL8, PTGS2, TIMP2, AGT |
| Cellular Growth and Proliferation | 3.76E-09-6.08E-04 | 26 | SLC4A2, MYCL1, FGF2, GRIA1, TAC1, IL6, IL31, AR, CCL2, TFAP2A, IL2, FGF18, HCK, BCCIP, AGT, TIMP2, IL3, CACNA1D, RRAD, THBS1, IL10, TRPM6, PRKG1, HABP2, PTGS2, TXNRD2 |
| Cellular Movement | 3.32E-08-6.08E-04 | 21 | IL3, FGF2, THBS1, IL10, TAC1, GPR34, IL6, AQP9, PRKG1, AR, HABP2, TFAP2A, CCL2, IL2, NAV1, HCK, EGLN3, CCL8, PTGS2, AGT, TIMP2 |
| Free Radical Scavenging | 2.13E-07-1.97E-04 | 13 | IL3, IL10, FGF2, UCP1, TAC1, IL6, CCL2, KL, IL2, HCK, PTGS2, TXNRD2, AGT |
| Cellular Development | 1.05E-06-6.08E-04 | 25 | FGF2, GRIA1, TAC1, IL6, IL31, AR, CCL2, TFAP2A, FGF18, IL2, HCK, BCCIP, TIMP2, AGT, IL3, CACNA1D, THBS1, IL10, RRAD, MLF1, TRPM6, PRKG1, HABP2, PTGS2, TXNRD2 |
| Lipid Metabolism | 1.18E-06-5.44E-04 | 19 | CROT, IL3, FGF2, RRAD, IL10, INPP5E, ACSL6, UCP1, TAC1, IL6, GPAM, AQP9, AR, CCL2, KL, IL2, PDE8B, PTGS2, AGT |
| Small Molecule Biochemistry | 1.18E-06-5.44E-04 | 27 | CROT, FGF2, GRIA1, UCP1, TAC1, IL6, AR, CCL2, KL, IL2, EGLN3, AGT, IL3, CACNA1D, IL10, THBS1, RRAD, INPP5E, ACSL6, GPAM, KCNA3, GABBR2, AQP9, PRKG1, PDE8B, PTGS2, TXNRD2 |
| Cell Signaling | 1.35E-06-2.37E-04 | 15 | IL3, CACNA1D, FGF2, THBS1, TAC1, IL6, GABBR2, PRKG1, CCL2, IL2, KL, HCK, CCL8, PTGS2, AGT |
| Molecular Transport | 1.35E-06-5.02E-04 | 32 | CROT, SLC4A2, FGF2, GRIA1, UCP1, TAC1, IL6, AR, CCL2, KL, IL2, HCK, EGLN3, CCL8, GABRA1, AGT, IL3, CACNA1D, IL10, THBS1, RRAD, INPP5E, ACSL6, AP1S1, TRPM6, GABBR2, KCNA3, GPAM, AQP9, PRKG1, BSND, PTGS2 |
| Vitamin and Mineral Metabolism | 1.35E-06-2.37E-04 | 15 | IL3, CACNA1D, FGF2, TAC1, IL6, AR, PRKG1, CCL2, KL, IL2, HCK, PDE8B, CCL8, PTGS2, AGT |
| DNA Replication, Recombination, and Repair | 1.94E-06-3.72E-05 | 10 | AR, FGF2, IL2, IL10, RRAD, TAC1, IL6, PTGS2, TIMP2, AGT |
| Nucleic Acid Metabolism | 1.94E-06-1.52E-04 | 9 | GABBR2, AR, IL2, FGF2, THBS1, TAC1, IL6, PTGS2, AGT |
| Gene Expression | 1.95E-06-5.02E-04 | 12 | IL3, AR, KRT6B, TFAP2A, FGF2, IL2, IL10, UCP1, HCK, TAC1, IL6, AGT |
| Cellular Function and Maintenance | 2.68E-06-6.08E-04 | 20 | IL3, CACNA1D, FGF2, IL10, UCP1, TAC1, IL6, GABBR2, KCNA3, AR, PRKG1, CCL2, KL, IL2, BSND, HCK, CCL8, PTGS2, GABRA1, AGT |
| Cell Death | 4.38E-06-6.42E-04 | 17 | IL3, IL10, FGF2, THBS1, TAC1, IL6, AR, CCL2, TFAP2A, KL, IL2, FGF18, HCK, EGLN3, PTGS2, AGT, TIMP2 |
| Amino Acid Metabolism | 9.25E-06-9.25E-06 | 5 | GABBR2, FGF2, IL10, TAC1, IL6 |
| Cell Morphology | 1.02E-05-4.61E-04 | 23 | IL3, CACNA1D, THBS1, FGF2, IL10, GRIA1, MYOZ1, TAC1, IL6, KCNA3, AQP9, AR, PRKG1, CCL2, TFAP2A, KL, IL2, HCK, EGLN3, CCL8, PTGS2, AGT, TIMP2 |
| Drug Metabolism | 1.19E-05-2.1E-04 | 9 | IL3, IL2, IL10, GRIA1, TAC1, EGLN3, PTGS2, IL6, AGT |
| Carbohydrate Metabolism | 1.32E-05-5.59E-04 | 16 | THBS1, RRAD, IL10, FGF2, INPP5E, UCP1, TAC1, IL6, GPAM, KCNA3, AQP9, CCL2, IL2, FUT9, PTGS2, AGT |
| Cell Cycle | 1.43E-05-6.08E-04 | 15 | IL3, IL10, THBS1, FGF2, RRAD, TAC1, MLF1, IL6, PES1, PRKG1, AR, IL2, PTGS2, TIMP2, AGT |
| Post-Translational Modification | 3.51E-05-3.51E-05 | 7 | IL3, FGF2, THBS1, IL2, HCK, TAC1, AGT |
| Cellular Assembly and Organization | 6.62E-05-3.78E-04 | 5 | AR, PRKG1, FGF18, FGF2, AGT |
| Protein Synthesis | 6.62E-05-4.38E-04 | 14 | IL3, CACNA1D, THBS1, FGF2, IL10, IL6, GPAM, KCNA3, AR, IL2, HCK, EGLN3, PTGS2, AGT |
| Energy Production | 1.21E-04-1.21E-04 | 5 | AR, UCP1, TAC1, IL6, AGT |

### Supplementary Table 5. Diseases and disorders significantly associated with the genes that were upregulated in lymphocytes in response to rapamycin treatment.

| **Category** | **p-value** | **No. of molecules** |
| --- | --- | --- |
| Cardiovascular Disease | 8.68E-10-6.76E-04 | 128 |
| Cancer | 1.03E-09-7.49E-04 | 278 |
| Organismal Injury and Abnormalities | 9.13E-09-4.73E-04 | 110 |
| Reproductive System Disease | 4.79E-08-7.47E-04 | 177 |
| Nutritional Disease | 5.57E-08-7.6E-04 | 55 |
| Neurological Disease | 2.55E-06-7.6E-04 | 140 |
| Inflammatory Response | 6.56E-06-6.1E-04 | 74 |
| Gastrointestinal Disease | 6.67E-06-7.49E-04 | 144 |
| Hepatic System Disease | 6.67E-06-7.13E-04 | 24 |
| Inflammatory Disease | 6.67E-06-7.13E-04 | 77 |
| Developmental Disorder | 1.07E-05-7.47E-04 | 81 |
| Connective Tissue Disorders | 1.25E-05-7.04E-04 | 52 |
| Skeletal and Muscular Disorders | 1.25E-05-7.04E-04 | 49 |
| Hereditary Disorder | 1.37E-05-5.47E-04 | 125 |
| Respiratory Disease | 1.94E-05-4.57E-04 | 76 |
| Endocrine System Disorders | 2.46E-05-7.49E-04 | 115 |
| Hematological Disease | 2.72E-05-7.2E-04 | 77 |
| Metabolic Disease | 9.05E-05-1.55E-04 | 72 |
| Immunological Disease | 1.32E-04-7.2E-04 | 67 |
| Psychological Disorders | 1.76E-04-7.6E-04 | 51 |
| Renal and Urological Disease | 1.86E-04-7.47E-04 | 17 |
| Dermatological Diseases and Conditions | 2.91E-04-6.36E-04 | 34 |
| Infectious Disease | 4.61E-04-4.61E-04 | 7 |
| Ophthalmic Disease | 4.73E-04-4.73E-04 | 13 |

### Supplementary Table 6. Physiological systems significantly associated with the genes that were upregulated in lymphocytes in response to rapamycin treatment.

| **Category** | **p-value** | **No. of molecules** |
| --- | --- | --- |
| Nervous System Development and Function | 3.86E-10-7.41E-04 | 183 |
| Tissue Development | 1.22E-09-7.17E-04 | 214 |
| Cardiovascular System Development and Function | 1.77E-09-5.81E-04 | 124 |
| Skeletal and Muscular System Development and Function | 5.34E-09-6.34E-04 | 114 |
| Connective Tissue Development and Function | 1.51E-08-7.04E-04 | 78 |
| Embryonic Development | 1.51E-08-7.17E-04 | 150 |
| Organ Development | 1.51E-08-6.34E-04 | 142 |
| Organ Morphology | 1.51E-08-7.04E-04 | 121 |
| Organismal Development | 1.51E-08-7.17E-04 | 207 |
| Tissue Morphology | 6.66E-08-7.41E-04 | 164 |
| Organismal Functions | 7.14E-08-9.93E-05 | 37 |
| Organismal Survival | 7.48E-07-4.92E-05 | 121 |
| Visual System Development and Function | 5.55E-06-5.12E-04 | 48 |
| Reproductive System Development and Function | 6.19E-06-7.04E-04 | 36 |
| Hematological System Development and Function | 7.74E-06-6.23E-04 | 92 |
| Renal and Urological System Development and Function | 7.83E-06-6.11E-04 | 46 |
| Behavior | 1.03E-05-4.64E-04 | 69 |
| Hepatic System Development and Function | 2.2E-05-3.35E-04 | 20 |
| Endocrine System Development and Function | 2.82E-05-7.17E-04 | 44 |
| Immune Cell Trafficking | 2.99E-05-6.23E-04 | 52 |
| Lymphoid Tissue Structure and Development | 4.93E-05-8.91E-05 | 28 |
| Tumor Morphology | 5.7E-05-5.56E-04 | 37 |
| Hair and Skin Development and Function | 6.03E-05-7.51E-05 | 20 |
| Digestive System Development and Function | 9.65E-05-5.37E-04 | 63 |
| Hematopoiesis | 1.03E-04-5.37E-04 | 33 |
| Respiratory System Development and Function | 1.4E-04-3.89E-04 | 33 |

### Supplementary Table 7. Networks significantly associated with the genes that were upregulated in lymphocytes in response to rapamycin treatment.

| **Network** | **Score** | **Focus Molecules** | **Top Functions** |
| --- | --- | --- | --- |
| 1 | 36 | 27 | Cellular Development, Nervous System Development and Function, Visual System Development and Function |
| 2 | 35 | 27 | Cancer, Endocrine System Disorders, Cell Cycle |
| 3 | 34 | 26 | Cellular Growth and Proliferation, Hematological System Development and Function, Hematopoiesis |
| 4 | 29 | 24 | Increased Levels of ALT, Cell-To-Cell Signaling and Interaction, Tissue Development |
| 5 | 29 | 27 | Embryonic Development, Organ Development, Organismal Development |
| 6 | 28 | 23 | Nervous System Development and Function, Cell-To-Cell Signaling and Interaction, Connective Tissue Disorders |
| 7 | 26 | 22 | Behavior, Carbohydrate Metabolism, Small Molecule Biochemistry |
| 8 | 26 | 22 | Cellular Movement, Carbohydrate Metabolism, Cellular Development |
| 9 | 25 | 21 | Tissue Morphology, Connective Tissue Development and Function, Embryonic Development |
| 10 | 23 | 20 | Endocrine System Development and Function, Small Molecule Biochemistry, Cellular Assembly and Organization |
| 11 | 23 | 20 | Cell Signaling, Molecular Transport, Nucleic Acid Metabolism |
| 12 | 22 | 22 | Cell Morphology, Nervous System Development and Function, Hereditary Disorder |
| 13 | 21 | 19 | Cardiovascular System Development and Function, Cell-To-Cell Signaling and Interaction, Cellular Development |
| 14 | 21 | 20 | Developmental Disorder, Hematological Disease, Hereditary Disorder |
| 15 | 20 | 18 | Cancer, Dermatological Diseases and Conditions, Lipid Metabolism |
| 16 | 20 | 18 | Cancer, Cell Cycle, Cellular Growth and Proliferation |
| 17 | 18 | 17 | Reproductive System Disease, Lipid Metabolism, Post-Translational Modification |
| 18 | 18 | 17 | Reproductive System Disease, Cellular Development, Hematological System Development and Function |
| 19 | 18 | 17 | Cancer, Reproductive System Disease, Cell Morphology |
| 20 | 18 | 17 | Developmental Disorder, Hereditary Disorder, Ophthalmic Disease |
| 21 | 18 | 17 | Lipid Metabolism, Small Molecule Biochemistry, Developmental Disorder |
| 22 | 18 | 17 | Infectious Disease, Cell Morphology, Embryonic Development |
| 23 | 18 | 18 | Cancer, Infectious Disease, Cardiovascular Disease |
| 24 | 16 | 16 | Drug Metabolism, Nucleic Acid Metabolism, Small Molecule Biochemistry |
| 25 | 16 | 16 | Developmental Disorder, Hereditary Disorder, Cancer |

### Supplementary Table 8. Diseases and disorders significantly associated with the genes that were downregulated in lymphocytes in response to rapamycin treatment.

| **Category** | **p-value** | **No. of molecules** |
| --- | --- | --- |
| Inflammatory Response | 2.35E-09-6.08E-04 | 17 |
| Organismal Injury and Abnormalities | 1.15E-08-6.42E-04 | 19 |
| Renal and Urological Disease | 1.15E-08-1.62E-04 | 10 |
| Respiratory Disease | 1.76E-08-4.58E-04 | 8 |
| Cardiovascular Disease | 6.76E-08-5.57E-04 | 21 |
| Cancer | 1.54E-06-5.32E-04 | 21 |
| Connective Tissue Disorders | 6.76E-06-5.02E-04 | 20 |
| Immunological Disease | 6.76E-06-7.18E-05 | 18 |
| Inflammatory Disease | 6.76E-06-5.32E-04 | 20 |
| Skeletal and Muscular Disorders | 6.76E-06-5.02E-04 | 26 |
| Hypersensitivity Response | 8.7E-06-3.28E-04 | 6 |
| Hematological Disease | 1.71E-05-5.85E-04 | 10 |
| Infectious Disease | 1.71E-05-6.08E-04 | 7 |
| Gastrointestinal Disease | 2.18E-05-5.32E-04 | 14 |
| Dermatological Diseases and Conditions | 3.35E-05-3.35E-05 | 5 |
| Developmental Disorder | 4.56E-05-1.84E-04 | 8 |
| Neurological Disease | 6.29E-05-3.35E-04 | 19 |
| Nutritional Disease | 7.13E-05-7.13E-05 | 8 |
| Reproductive System Disease | 8.48E-05-8.48E-05 | 10 |
| Metabolic Disease | 1.62E-04-1.62E-04 | 4 |
| Hereditary Disorder | 1.92E-04-1.92E-04 | 3 |
| Hepatic System Disease | 3.78E-04-5.32E-04 | 6 |

### Supplementary Table 9. Physiological systems significantly associated with the genes that were downregulated in lymphocytes in response to rapamycin treatment.

| **Category** | **p-value** | **No. of molecules** |
| --- | --- | --- |
| Hematological System Development and Function | 2.35E-09-6.08E-04 | 17 |
| Immune Cell Trafficking | 2.35E-09-6.08E-04 | 17 |
| Connective Tissue Development and Function | 3.76E-09-6.08E-04 | 18 |
| Skeletal and Muscular System Development and Function | 3.76E-09-4.58E-04 | 23 |
| Tissue Development | 9.22E-09-6.08E-04 | 23 |
| Cardiovascular System Development and Function | 1.87E-08-6.08E-04 | 22 |
| Organismal Development | 5.57E-08-6.08E-04 | 34 |
| Tissue Morphology | 2.46E-07-6.08E-04 | 15 |
| Hematopoiesis | 1.05E-06-6.08E-04 | 11 |
| Humoral Immune Response | 1.05E-06-5.49E-04 | 5 |
| Tumor Morphology | 1.54E-06-5.99E-04 | 16 |
| Cell-mediated Immune Response | 2.88E-06-6.08E-04 | 9 |
| Endocrine System Development and Function | 4.79E-06-6.08E-04 | 15 |
| Hair and Skin Development and Function | 1.64E-05-6.08E-04 | 3 |
| Organismal Functions | 2.21E-05-5.02E-04 | 10 |
| Renal and Urological System Development and Function | 2.21E-05-2.21E-05 | 2 |
| Organismal Survival | 2.29E-05-2.54E-04 | 12 |
| Lymphoid Tissue Structure and Development | 2.77E-05-6.08E-04 | 8 |
| Organ Development | 3.91E-05-4.58E-04 | 16 |
| Hepatic System Development and Function | 4.52E-05-6.08E-04 | 6 |
| Embryonic Development | 5.36E-05-5.39E-04 | 21 |
| Organ Morphology | 5.36E-05-6.08E-04 | 18 |
| Nervous System Development and Function | 6.62E-05-6.42E-04 | 14 |
| Behavior | 2.18E-04-2.48E-04 | 7 |
| Digestive System Development and Function | 2.19E-04-2.19E-04 | 2 |
| Reproductive System Development and Function | 3.28E-04-3.28E-04 | 2 |

### Supplementary Table 10. Networks significantly associated with the genes that were downregulated in lymphocytes in response to rapamycin treatment.

| **Network** | **Score** | **Focus Molecules** | **Top Functions** |
| --- | --- | --- | --- |
| 1 | 33 | 16 | Embryonic Development, Organ Development, Organismal Development |
| 2 | 23 | 12 | Gene Expression, Neurological Disease, Tissue Morphology |
| 3 | 18 | 10 | Cardiovascular System Development and Function, Organismal Development, Tissue Morphology |
| 4 | 18 | 10 | Cell-To-Cell Signaling and Interaction, Tissue Development, Drug Metabolism |
| 5 | 16 | 9 | Neurological Disease, Psychological Disorders, Nutritional Disease |
| 6 | 14 | 9 | Developmental Disorder, Skeletal and Muscular System Development and Function, Tissue Morphology |
| 7 | 10 | 6 | Lipid Metabolism, Small Molecule Biochemistry, Cardiovascular System Development and Function |
| 8 | 9 | 6 | Nervous System Development and Function, Endocrine System Development and Function, Lipid Metabolism |
| 9 | 8 | 5 | Cell-mediated Immune Response, Cellular Development, Cellular Function and Maintenance |

### Supplementary Table 11. Molecular and cellular functions associated with the altered rapamycin response elements (112 genes) in the frontal lobe of mild AD patients (limbic stage) compared to control.

| **Category** | **p-value** | **No. of genes** | **Molecules** |
| --- | --- | --- | --- |
| Cell Cycle | 1.35E-06-5.38E-03 | 11 | GLI2, RUNX1T1, MYF6, PIM1, SMAD3, PBX1, CDKN2C, PRKCH, BMP7, IL6, NR3C1 |
| Cell Death and Survival | 5.85E-06-5.38E-03 | 34 | GLI2, CEACAM6, SMAD3, PBX1, CDKN2C, FN3K, IL6, TRPV1, NR3C1, TRIB1, RHO, CCL2, RHOT1, PIM1, LUM, RPS6KB2, TNFRSF10C, SERPINE1, KLF2, NCKAP1, CCR1, POU4F2, MFAP5, PHLDA3, CD69, DCD, CCL11, HDAC5, RUNX1T1, GLP1R, CRP, PRKCH, BMP7, NTSR1 |
| Cellular Movement | 1.12E-05-5.38E-03 | 28 | GLI2, SMAD3, CDKN2C, IL6, TRPV1, HOXD10, SEMA4C, BARX2, TRIB1, CCL2, RHO, PIM1, STARD13, LUM, EBF2, SERPINE1, KLF2, CCL1, NCKAP1, CCR1, POU4F2, TM4SF4, SEMA5A, CD69, RELN, CCL11, CRP, BMP7 |
| Cellular Growth and Proliferation | 1.44E-05-5.38E-03 | 40 | LOXL4, GLI2, CEACAM6, SMAD3, PBX1, CDKN2C, IL6, TRPV1, NR3C1, SEMA4C, ADAMTS2, SERPIND1, COL1A2, TRIB1, CCL2, MYF6, PIM1, LECT1, STARD13, LUM, RPS6KB2, SERPINE1, KLF2, CCR1, CACNA1D, POU4F2, TM4SF4, CD69, DCD, CCL11, HDAC5, B4GALT6, RUNX1T1, ABTB1, GLP1R, CRP, SLC22A18, PRKCH, BMP7, FGF11 |
| Cellular Function and Maintenance | 2.49E-05-5.38E-03 | 24 | CCR1, POU4F2, CEACAM6, CACNA1D, MFAP5, SMAD3, CD69, TRPV1, IL6, RELN, CCL11, NR3C1, ADAMTS2, COL1A2, RUNX1T1, CCL2, PIM1, RHOT1, CRP, GLRA1, PRKCH, SERPINE1, KLF2, CCL1 |
| Molecular Transport | 2.49E-05-5.38E-03 | 24 | CCR1, CACNA1D, SMAD3, SLC18A3, CDKN2C, SLC22A7, TRPV1, IL6, RELN, CCL11, NR3C1, SLC38A3, B4GALT6, CCL2, GLP1R, CRP, GLRA1, SLC22A18, ZBTB20, EBF2, BMP7, SERPINE1, KLF2, CCL1 |
| Cellular Development | 3.21E-05-5.38E-03 | 41 | CEACAM6, GLI2, SMAD3, PBX1, CDKN2C, TRPV1, IL6, NR3C1, SEMA4C, ADAMTS2, BARX2, CTDSP1, RHO, CCL2, MYF6, PIM1, LECT1, LUM, STARD13, RPS6KB2, EBF2, SERPINE1, KLF2, CCL1, NCKAP1, CCR1, CACNA1D, POU4F2, SEMA5A, CD69, DCD, RELN, CCL11, HDAC5, B4GALT6, RUNX1T1, ABTB1, GLP1R, CRP, PRKCH, BMP7 |
| Small Molecule Biochemistry | 4.13E-05-5.38E-03 | 19 | CCR1, CACNA1D, SMAD3, CDKN2C, SLC22A7, TRPV1, IL6, CCL11, NR3C1, SLC38A3, B4GALT6, CCL2, GLP1R, CRP, ZBTB20, BMP7, EBF2, SERPINE1, CCL1 |
| Post-Translational Modification | 4.92E-05-4.92E-05 | 15 | CCR1, MAPK4, CDKN2C, FN3K, CARD14, TRPV1, CCL11, RELN, CCL2, RHO, PIM1, CRP, RPS6KB2, PRKCH, BMP7 |
| Cell-To-Cell Signaling and Interaction | 7.7E-05-5.38E-03 | 22 | CCR1, GLI2, CEACAM6, SEMA5A, CD69, SMAD3, PVRL3, SLC22A7, IL6, TRPV1, RELN, CCL11, NR3C1, CCL2, PIM1, CRP, BMP7, FGF11, EBF2, SERPINE1, KLF2, CCL1 |
| Cell Morphology | 8.55E-05-5.38E-03 | 24 | CCR1, POU4F2, CACNA1D, GLI2, SEMA5A, SMAD3, PVRL3, PBX1, CDKN2C, TRPV1, IL6, CREB3L4, RELN, NR3C1, ADAMTS2, CCL2, RHO, PIM1, LUM, STARD13, PRKCH, BMP7, SERPINE1, KLF2 |
| Cell Signaling | 9.49E-05-3.32E-03 | 13 | CCR1, CACNA1D, TRPV1, IL6, RELN, CCL11, NR3C1, CCL2, GLP1R, GLRA1, KLF2, CCL1, NTSR1 |
| Vitamin and Mineral Metabolism | 9.49E-05-3.32E-03 | 11 | CCR1, CACNA1D, CCL2, GLP1R, IL6, TRPV1, CCL11, RELN, NR3C1, KLF2, CCL1 |
| Gene Expression | 1.65E-04-5.38E-03 | 25 | GLI2, SMAD3, PBX1, CDKN2C, IL6, HMGCS2, NR3C1, HOXD10, BARX2, CTDSP1, CCL2, MYF6, LUM, RPS6KB2, ZBTB20, EBF2, KLF2, NCKAP1, POU4F2, CREB3L4, CCL11, HDAC5, RUNX1T1, GLP1R, BMP7 |
| Nucleic Acid Metabolism | 2.83E-04-2.83E-04 | 2 | GLP1R, CRP |
| Cellular Assembly and Organization | 6.05E-04-5.38E-03 | 10 | COL1A2, GLI2, RHO, MFAP5, SEMA5A, LUM, BMP7, RELN, NR3C1, ADAMTS2 |
| Lipid Metabolism | 1.52E-03-5.38E-03 | 11 | B4GALT6, CCL2, GLP1R, SMAD3, CRP, CDKN2C, BMP7, IL6, CCL11, SERPINE1, NR3C1 |
| Free Radical Scavenging | 2.05E-03-2.05E-03 | 3 | CCL2, CCL11, CCL1 |
| Protein Synthesis | 4.06E-03-4.06E-03 | 9 | CCR1, CACNA1D, LECT1, GLP1R, CRP, ZBTB20, IL6, SERPINE1, NR3C1 |
| Amino Acid Metabolism | 4.94E-03-5.38E-03 | 4 | IL6, TRPV1, SLC22A7, SLC38A3 |
| Cellular Compromise | 5.38E-03-5.38E-03 | 3 | PIM1, TRPV1, NR3C1 |

### Supplementary Table 12. Diseases and disorders significantly associated with the altered rapamycin response elements in the frontal lobe of mild AD patients (limbic stage) compared to control (112 genes)

| **Category** | **p-value** | **No. of genes** | **Molecules** |
| --- | --- | --- | --- |
| Renal and Urological Disease | 1.59E-07-5.38E-03 | 11 | CCR1, CACNA1D, CCL2, SMAD3, CRP, CDKN2C, BMP7, IL6, TRPV1, SERPINE1, NR3C1 |
| Cardiovascular Disease | 8.24E-07-5.38E-03 | 17 | CCR1, CACNA1D, SMAD3, PBX1, IL6, NR3C1, SERPIND1, TRIB1, CCL2, PIM1, GLP1R, CRP, RPS6KB2, BMP7, PRKCH, SERPINE1, KLF2 |
| Inflammatory Response | 1.12E-05-5.38E-03 | 15 | CCR1, CEACAM6, CD69, SMAD3, IL6, TRPV1, CCL11, NR3C1, HDAC5, CCL2, CRP, LUM, SERPINE1, KLF2, CCL1 |
| Infectious Disease | 1.24E-05-5.38E-03 | 12 | CCR1, CEACAM6, CCL2, SMAD3, CD69, CRP, LUM, TRPV1, IL6, CCL11, SERPINE1, NR3C1 |
| Organismal Injury and Abnormalities | 1.34E-05-5.38E-03 | 21 | CCR1, CACNA1D, GLI2, SMAD3, PBX1, IL6, TRPV1, NR3C1, ADAMTS2, BARX2, SERPIND1, COL1A2, CCL2, PIM1, GLP1R, CRP, LUM, PRKCH, BMP7, SERPINE1, KLF2 |
| Gastrointestinal Disease | 1.9E-05-5.38E-03 | 16 | CCR1, GLI2, TM4SF4, SMAD3, CD69, PVRL3, CDKN2C, IL6, TRPV1, CCL11, NR3C1, COL1A2, CCL2, CRP, BMP7, SERPINE1 |
| Hepatic System Disease | 1.9E-05-5.38E-03 | 7 | COL1A2, CCR1, CCL2, SMAD3, BMP7, IL6, SERPINE1 |
| Cancer | 1.91E-05-5.38E-03 | 46 | HCAR3, CEACAM6, GLI2, SMAD3, PVRL3, PBX1, CDKN2C, IL6, TRPV1, SHE, NR3C1, ADAMTS2, HOXD10, SERPIND1, COL1A2, TRIB1, CCL2, PIM1, LUM, RPS6KB2, ZBTB20, SERPINE1, HYDIN, KLF2, CCR1, CACNA1D, MGC24103, TM4SF4, MFAP5, MOXD1, CD69, SEMA5A, DCD, SLC18A3, CREB3L4, CCL11, RELN, HDAC5, RUNX1T1, GLP1R, CRP, SLC22A18, BMP7, PRKCH, FCGBP, NTSR1 |
| Connective Tissue Disorders | 2.86E-05-5.38E-03 | 16 | CCR1, GLI2, SMAD3, CD69, IL6, NR3C1, ADAMTS2, HOXD10, COL1A2, CCL2, LUM, TNFRSF10C, BMP7, SERPINE1, KLF2, CCL1 |
| Inflammatory Disease | 2.86E-05-5.38E-03 | 15 | CCR1, CD69, SMAD3, IL6, TRPV1, CCL11, NR3C1, ADAMTS2, CCL2, LUM, CRP, TNFRSF10C, SERPINE1, KLF2, CCL1 |
| Respiratory Disease | 2.86E-05-5.38E-03 | 8 | CCL2, SMAD3, CRP, IL6, CCL11, SERPINE1, NR3C1, CCL1 |
| Skeletal and Muscular Disorders | 2.86E-05-5.38E-03 | 18 | CCR1, GLI2, CD69, SMAD3, PBX1, IL6, NR3C1, HOXD10, ADAMTS2, COL1A2, CCL2, MYF6, LUM, TNFRSF10C, BMP7, SERPINE1, KLF2, CCL1 |
| Ophthalmic Disease | 9.67E-05-5.38E-03 | 5 | RHO, CRP, BMP7, IL6, NR3C1 |
| Developmental Disorder | 1.25E-04-5.38E-03 | 11 | COL1A2, GLI2, MYF6, CCL2, SMAD3, PBX1, BMP7, NR3C1, KLF2, HOXD10, ADAMTS2 |
| Metabolic Disease | 3.42E-04-5.38E-03 | 9 | COL1A2, CCL2, GLP1R, CRP, BMP7, IL6, SERPINE1, HMGCS2, ADAMTS2 |
| Hypersensitivity Response | 4.23E-04-5.38E-03 | 5 | CCR1, CCL2, SMAD3, IL6, CCL11 |
| Endocrine System Disorders | 4.5E-04-5.38E-03 | 7 | CCL2, GLP1R, CDKN2C, BMP7, IL6, SERPINE1, NR3C1 |
| Immunological Disease | 4.5E-04-5.38E-03 | 9 | CCR1, CD69, CRP, CDKN2C, TNFRSF10C, IL6, CCL11, NR3C1, CCL1 |
| Dermatological Diseases and Conditions | 7.84E-04-5.38E-03 | 7 | COL1A2, SMAD3, PRKCH, TRPV1, NR3C1, ADAMTS2, SERPIND1 |
| Reproductive System Disease | 7.96E-04-3.26E-03 | 16 | CEACAM6, MGC24103, MFAP5, MOXD1, CDKN2C, IL6, TRPV1, NR3C1, HOXD10, RUNX1T1, CCL2, LUM, CRP, BMP7, SERPINE1, KLF2 |
| Hematological Disease | 9.72E-04-5.38E-03 | 18 | HCAR3, CD69, PBX1, CDKN2C, IL6, TRPV1, CCL11, NR3C1, SERPIND1, HDAC5, TRIB1, RUNX1T1, CCL2, PIM1, CRP, LUM, SERPINE1, CCL1 |
| Neurological Disease | 1.68E-03-5.38E-03 | 15 | GLI2, CACNA1D, SEMA5A, CDKN2C, TRPV1, IL6, RELN, NR3C1, HOXD10, HDAC5, CCL2, LUM, BMP7, EBF2, SERPINE1 |
| Hereditary Disorder | 2.5E-03-5.38E-03 | 6 | COL1A2, MYF6, CCL2, HMGCS2, NR3C1, ADAMTS2 |
| Auditory Disease | 5.38E-03-5.38E-03 | 1 | NR3C1 |

### Supplementary Table 13. Physiological system development and function significantly associated with the altered rapamycin response elements in the frontal lobe of mild AD patients (limbic stage) compared to control (112 genes)

| **Category** | **p-value** | **No. of genes** | **Molecules** |
| --- | --- | --- | --- |
| Hematological System Development and Function | 1.12E-05-5.38E-03 | 18 | CCR1, CEACAM6, CD69, SMAD3, PBX1, TRPV1, IL6, CCL11, NR3C1, SERPIND1, RUNX1T1, CCL2, PIM1, CRP, LUM, SERPINE1, KLF2, CCL1 |
| Immune Cell Trafficking | 1.12E-05-5.38E-03 | 14 | CCR1, CEACAM6, CD69, SMAD3, TRPV1, IL6, CCL11, NR3C1, CCL2, LUM, CRP, SERPINE1, KLF2, CCL1 |
| Respiratory System Development and Function | 1.4E-05-2.5E-03 | 11 | GLI2, CCL2, PBX1, BMP7, EBF2, IL6, SERPINE1, HYDIN, NR3C1, KLF2, ADAMTS2 |
| Embryonic Development | 2.62E-05-5.38E-03 | 27 | GLI2, SMAD3, PVRL3, PBX1, IL6, NR3C1, SEMA4C, ADAMTS2, HOXD10, MYF6, CCL2, RHO, LECT1, LUM, ZBTB20, EBF2, SERPINE1, KLF2, HYDIN, CCL1, NCKAP1, CCR1, POU4F2, SEMA5A, RELN, HDAC5, BMP7 |
| Organismal Development | 2.62E-05-5.38E-03 | 35 | GLI2, SMAD3, PVRL3, PBX1, CDKN2C, TRPV1, IL6, NR3C1, ADAMTS2, HOXD10, COL1A2, TRIB1, CCL2, RHO, MYF6, PIM1, LECT1, STARD13, LUM, ZBTB20, EBF2, SERPINE1, HYDIN, KLF2, NCKAP1, CCL1, CCR1, CACNA1D, POU4F2, SEMA5A, CCL11, HDAC5, GLP1R, PRKCH, BMP7 |
| Lymphoid Tissue Structure and Development | 3.21E-05-4.35E-03 | 9 | CCR1, RUNX1T1, CCL2, PIM1, CD69, SMAD3, PBX1, BMP7, IL6 |
| Tissue Development | 6.13E-05-5.38E-03 | 32 | GLI2, SMAD3, PVRL3, PBX1, TRPV1, IL6, NR3C1, HOXD10, ADAMTS2, SERPIND1, CCL2, RHO, MYF6, LECT1, PIM1, LUM, ZBTB20, EBF2, SERPINE1, HYDIN, KLF2, NCKAP1, CCL1, CCR1, POU4F2, CD69, SEMA5A, RELN, CCL11, HDAC5, CRP, BMP7 |
| Tissue Morphology | 7.59E-05-5.38E-03 | 30 | GLI2, SMAD3, PVRL3, PBX1, CDKN2C, TRPV1, IL6, NR3C1, SERPIND1, RHO, MYF6, CCL2, PIM1, LUM, RPS6KB2, EBF2, SERPINE1, KLF2, CCR1, POU4F2, CACNA1D, SEMA5A, CD69, SLC18A3, CREB3L4, CCL11, GLP1R, CRP, PRKCH, BMP7 |
| Hair and Skin Development and Function | 8.55E-05-5.38E-03 | 9 | GLI2, SMAD3, LUM, PRKCH, IL6, TRPV1, NR3C1, ADAMTS2, BARX2 |
| Skeletal and Muscular System Development and Function | 8.55E-05-5.38E-03 | 20 | CCR1, GLI2, SMAD3, PBX1, IL6, NR3C1, SEMA4C, HOXD10, BARX2, HDAC5, TRIB1, MYF6, CCL2, PIM1, LECT1, LUM, BMP7, SERPINE1, KLF2, CCL1 |
| Cardiovascular System Development and Function | 1.05E-04-5.38E-03 | 22 | CCR1, GLI2, SEMA5A, SMAD3, PBX1, TRPV1, IL6, CCL11, NR3C1, HOXD10, ADAMTS2, HDAC5, COL1A2, CCL2, PIM1, LECT1, GLP1R, CRP, BMP7, SERPINE1, KLF2, CCL1 |
| Organ Morphology | 1.09E-04-5.38E-03 | 16 | CCR1, GLI2, CACNA1D, SMAD3, PBX1, CDKN2C, IL6, NR3C1, HOXD10, CCL2, MYF6, LECT1, LUM, BMP7, SERPINE1, KLF2 |
| Organ Development | 1.64E-04-5.38E-03 | 24 | CCR1, POU4F2, GLI2, SEMA5A, SMAD3, PVRL3, PBX1, IL6, NR3C1, ADAMTS2, HOXD10, HDAC5, RHO, MYF6, CCL2, LECT1, LUM, ZBTB20, EBF2, BMP7, SERPINE1, HYDIN, KLF2, CCL1 |
| Visual System Development and Function | 1.64E-04-5.38E-03 | 10 | POU4F2, RHO, SEMA5A, LUM, PVRL3, BMP7, EBF2, SERPINE1, NR3C1, CCL1 |
| Nervous System Development and Function | 1.7E-04-5.38E-03 | 26 | GLI2, DRP2, PBX1, CDKN2C, IL6, TRPV1, NR3C1, HOXD10, SEMA4C, CTDSP1, CCL2, RHO, RPS6KB2, ZBTB20, EBF2, SERPINE1, NCKAP1, CCR1, POU4F2, CACNA1D, SEMA5A, RELN, B4GALT6, GLRA1, BMP7, PRKCH |
| Behavior | 2.16E-04-2.16E-04 | 8 | GLP1R, GLRA1, SLC18A3, IL6, RELN, NR3C1, HOXD10, NTSR1 |
| Cell-mediated Immune Response | 2.18E-04-5.38E-03 | 8 | CCR1, CCL2, SMAD3, CD69, IL6, CCL11, KLF2, CCL1 |
| Connective Tissue Development and Function | 2.41E-04-5.38E-03 | 15 | GLI2, SMAD3, PBX1, TRPV1, IL6, NR3C1, HOXD10, HDAC5, MYF6, CCL2, LECT1, GLP1R, LUM, BMP7, KLF2 |
| Hematopoiesis | 4.96E-04-5.38E-03 | 10 | CCR1, RUNX1T1, CCL2, PIM1, SMAD3, CD69, PBX1, IL6, CCL11, KLF2 |
| Digestive System Development and Function | 5.16E-04-5.1E-03 | 7 | CACNA1D, GLI2, PVRL3, PBX1, BMP7, IL6, ADAMTS2 |
| Endocrine System Development and Function | 5.9E-04-5.38E-03 | 20 | CACNA1D, GLP1R, SMAD3, PBX1, CDKN2C, ZBTB20, EBF2, BMP7, IL6, NR3C1 |
| Hepatic System Development and Function | 7.84E-04-7.84E-04 | 2 | CCL2, SMAD3 |
| Tumor Morphology | 8.2E-04-4.12E-03 | 11 | CCL2, PIM1, SMAD3, LUM, CDKN2C, BMP7, TRPV1, IL6, CCL11, SERPINE1, NR3C1 |
| Organismal Functions | 1.18E-03-2.11E-03 | 9 | SMAD3, GLP1R, LUM, PRKCH, BMP7, IL6, TRPV1, SERPINE1, NTSR1 |
| Humoral Immune Response | 1.25E-03-1.25E-03 | 2 | SMAD3, IL6 |
| Reproductive System Development and Function | 1.81E-03-5.38E-03 | 5 | CDKN2C, BMP7, CREB3L4, IL6, NR3C1 |
| Renal and Urological System Development and Function | 3.27E-03-3.74E-03 | 5 | CCR1, PIM1, SMAD3, BMP7, SERPINE1 |
| Organismal Survival | 3.47E-03-3.47E-03 | 10 | CCR1, CCL2, PIM1, SMAD3, CDKN2C, ZBTB20, BMP7, IL6, SERPINE1, NR3C1 |

### Supplementary Table 14. Networks significantly associated with the altered rapamycin response elements in the frontal lobe of mild AD patients (limbic stage) compared to control (112 genes)

| **ID** | **Top Functions** | **Score** | **Focus Molecules** |
| --- | --- | --- | --- |
| 1 | Tissue Development, Cell Cycle, Immunological Disease | 35 | 17 |
| 2 | Connective Tissue Development and Function, Embryonic Development, Organ Development | 29 | 15 |
| 3 | Developmental Disorder, Hereditary Disorder, Cell-To-Cell Signaling and Interaction | 27 | 14 |
| 4 | Cardiovascular Disease, Endocrine System Disorders, Gastrointestinal Disease | 27 | 14 |
| 5 | Cell Cycle, Cellular Movement, Cell-mediated Immune Response | 25 | 13 |
| 6 | Hair and Skin Development and Function, Cellular Growth and Proliferation, Gastrointestinal Disease | 22 | 12 |
| 7 | Cellular Growth and Proliferation, Nervous System Development and Function, Behavior | 22 | 12 |

### Supplementary Table 15. Pathways significantly associated with the altered rapamycin response elements in the frontal lobe of mild AD patients (limbic stage) compared to control (112 genes)

| **Pathways** | **p-value** | **Ratio (no. of sig genes / total involved in pathway)** |
| --- | --- | --- |
| Glucocorticoid Receptor Signaling | 0.000 | 0.024 |
| IL-17 Signaling | 0.001 | 0.054 |
| Role of Hypercytokinemia/hyperchemokinemia in the Pathogenesis of Influenza | 0.001 | 0.068 |
| Role of IL-17F in Allergic Inflammatory Airway Diseases | 0.001 | 0.063 |
| Acute Phase Response Signaling | 0.002 | 0.028 |
| Atherosclerosis Signaling | 0.004 | 0.029 |
| Differential Regulation of Cytokine Production in Macrophages and T Helper Cells by IL-17A and IL-17F | 0.004 | 0.111 |
| PXR/RXR Activation | 0.006 | 0.035 |
| Hepatic Fibrosis / Hepatic Stellate Cell Activation | 0.007 | 0.027 |
| G-Protein Coupled Receptor Signaling | 0.010 | 0.013 |
| TGF-β Signaling | 0.010 | 0.034 |
| HMGB1 Signaling | 0.014 | 0.030 |
| Coagulation System | 0.014 | 0.053 |
| IL-17A Signaling in Fibroblasts | 0.015 | 0.050 |
| Spermine and Spermidine Degradation I | 0.021 | 0.071 |
| Corticotropin Releasing Hormone Signaling | 0.022 | 0.022 |
| Phospholipase C Signaling | 0.032 | 0.019 |
| Molecular Mechanisms of Cancer | 0.035 | 0.013 |
| Hepatic Cholestasis | 0.038 | 0.017 |
| TREM1 Signaling | 0.040 | 0.028 |
| Pyridoxal 5'-phosphate Salvage Pathway | 0.042 | 0.028 |
| Cell Cycle: G1/S Checkpoint Regulation | 0.043 | 0.030 |
| ERK5 Signaling | 0.046 | 0.031 |
| IL-17A Signaling in Airway Cells | 0.046 | 0.028 |
| Pathogenesis of Multiple Sclerosis | 0.048 | 0.111 |

### Supplementary Table 16. Molecular and cellular functions significantly associated with the altered rapamycin response elements in the frontal lobe of advanced AD patients (neocortical stage) compared to control (176 genes)

| **Category** | **p-value** | **No. of genes** | **Molecules** |
| --- | --- | --- | --- |
| Cell-To-Cell Signaling and Interaction | 9.89E-07-4.13E-03 | 37 | KCND2, CEACAM6, MAPK1, SMAD3, PVRL3, DLG2, CLU, SLC22A7, TRPV1, IL6, NR3C1, SCN8A, VEGFA, KCNAB1, CCL2, PIM1, CPLX2, TSC2, GFAP, SERPINE1, KLF2, CCL1, CCR1, SLC18A3, GPR176, RELN, CCL11, MECOM, APP, GABBR2, GLRA1, CRP, BMP7, BPI, IL1RAPL1, VSNL1, NTSR1 |
| Cellular Development | 5.77E-06-3.73E-03 | 53 | GLI2, MAPK1, TP73, SMAD3, IL6, ELAVL4, ADAMTS2, VEGFA, CTDSP1, LECT1, PIM1, LUM, TSC2, RNF128, EBF2, IRS2, SERPINE1, KLF2, HIST1H4A, SEMA5A, ACSL6, DCD, HDAC5, MECOM, RUNX1T1, RND3, PRKCH, BPI, IL1RAPL1, CEACAM6, CLU, ACVR2B, NR3C1, EIF4E, SEMA4C, SERPIND1, BARX2, CCL2, MYF6, STARD13, RPS6KB2, CCL1, NCKAP1, CCR1, CACNA1D, POU4F2, RELN, CCL11, APP, CUX1, ABTB1, SLC25A27, BMP7 |
| Cellular Function and Maintenance | 6.51E-06-4.04E-03 | 51 | MAPK1, TP73, SMAD3, CCNB2, IL6, SCN8A, VEGFA, PEX5L, PIM1, CPLX2, TSC2, RNF128 , IRS2, GFAP, SERPINE1, SLITRK1, KLF2, HYDIN, CEP68, MECOM, SCGN, RND3, GLRA1, RAB3C, BPI, PRKCH, IL1RAPL1, CEACAM6, PVRL3, CLU, TRPV1, TAOK1, DCLK1, NR3C1, DYNC2LI1, CCL2, STX1B, NCKAP1, CCL1, CCR1, CACNA1D, POU4F2, SLC18A3, RELN, CCL11, APP, GABBR2, CUX1, CRP, SLC25A27, BMP7 |
| Molecular Transport | 6.51E-06-3.73E-03 | 30 | TP73, SMAD3, CLU, SLC22A7, IL6, TRPV1, NR3C1, VEGFA, CCL2, PEX5L, STX1B, CPLX2, ZBTB20, IRS2, SERPINE1, KLF2, CCL1, CCR1, CACNA1D, SLC18A3, CCL11, RELN, APP, GABBR2, CUX1, SCGN, CRP, RAB3C, BMP7, IL1RAPL1 |
| Cellular Movement | 1.01E-05-3.8E-03 | 38 | GLI2, MAPK1, TP73, MMP16, SMAD3, CLU, CCNB2, IL6, TRPV1, DCLK1, HOXD10, SEMA4C, SCN8A, BARX2, VEGFA, CCL2, PIM1, STARD13, TSC2, LUM, GFAP, IRS2, EBF2, SERPINE1, KLF2, CCL1, NCKAP1, CCR1, POU4F2, SEMA5A, CCL11, RELN, APP, CUX1, RND3, CRP, BMP7, VSNL1 |
| Cell Signaling | 1.73E-05-2.34E-03 | 14 | CCR1, CACNA1D, TP73, TRPV1, IL6, RELN, CCL11, NR3C1, APP, VEGFA, SCGN, CCL2, KLF2, CCL1 |
| Vitamin and Mineral Metabolism | 1.73E-05-2.34E-03 | 14 | CCR1, CACNA1D, TP73, TRPV1, IL6, RELN, CCL11, NR3C1, APP, VEGFA, SCGN, CCL2, KLF2, CCL1 |
| Cell Death and Survival | 2.34E-05-3.94E-03 | 47 | GLI2, CEACAM6, MAPK1, TP73, SMAD3, CLU, SYCP3, FN3K, TRPV1, TAOK1, ELAVL4, ACVR2B, IL6, NR3C1, EIF4E, VEGFA, CCL2, PIM1, LUM, TSC2, RPS6KB2, SORBS2, GFAP, IRS2, TNFRSF10C, SERPINE1, KLF2, NCKAP1, CCR1, CACNA1D, POU4F2, MFAP5, PHLDA3, DCD, CCL11, HDAC5, APP, MECOM, RUNX1T1, RND3, CRP, SLC25A27, PRKCH, BPI, BMP7, VSNL1, NTSR1 |
| Cellular Growth and Proliferation | 4.01E-05-4.04E-03 | 41 | LOXL4, GLI2, CEACAM6, MAPK1, TP73, SMAD3, CLU, IL6, TRPV1, NR3C1, SEMA4C, EIF4E, ADAMTS2, SERPIND1, VEGFA, CCL2, MYF6, PIM1, LECT1, STARD13, LUM, TSC2, RPS6KB2, RNF128, IRS2, SERPINE1, KLF2, CCR1, POU4F2, ACSL6, DCD, CCL11, HDAC5, APP, MECOM, CUX1, RUNX1T1, ABTB1, BMP7, BPI, PRKCH |
| Cell Morphology | 4.81E-05-3.17E-03 | 44 | KCND2, GLI2, MAPK1, TP73, MMP16, SMAD3, PVRL3, CLU, FAT3, SYCP3, TRPV1, ELAVL4, IL6, DCLK1, NR3C1, EIF4E, DYNC2LI1, VEGFA, CCL2, PIM1, STARD13, TSC2, RNF128, EBF2, IRS2, GFAP, SERPINE1, SLITRK1, HYDIN, KLF2, NCKAP1, CCL1, CCR1, POU4F2, CACNA1D, SEMA5A, SLC18A3, CREB3L4, RELN, MECOM, APP, GABBR2, PRKCH, BMP7 |
| Cellular Assembly and Organization | 8.61E-05-4.04E-03 | 33 | GLI2, MAPK1, TP73, SMAD3, PVRL3, CLU, ELAVL4, TAOK1, IL6, DCLK1, NR3C1, DYNC2LI1, BARX2, VEGFA, CCL2, TSC2, STARD13, GFAP, SERPINE1, KLF2, HYDIN, SLITRK1, NCKAP1, POU4F2, SEMA5A, CEP68, SLC18A3, RELN, CCL11, APP, RND3, BMP7, IL1RAPL1 |
| Post-Translational Modification | 1.19E-04-3.07E-03 | 20 | CCR1, MAPK1, MAPK4, FN3K, CARD14, IL6, TAOK1, ACVR2B, TRPV1, CCL11, DCLK1, RELN, APP, VEGFA, CCL2, PIM1, CRP, RPS6KB2, PRKCH, BMP7 |
| Gene Expression | 1.52E-04-4.05E-03 | 37 | GLI2, MAPK1, TP73, SMAD3, IL6, ACVR2B, HMGCS2, DCLK1, NR3C1, EIF4E, HOXD10, BARX2, VEGFA, CTDSP1, MYF6, CCL2, PIM1, LUM, RPS6KB2, ZBTB20, EBF2, ABLIM2, KLF2, NCKAP1, POU4F2, BCOR, CREB3L4, CCL11, HDAC5, MECOM, APP, CUX1, RUNX1T1, ABTB1, IRX4, ATF7IP, BMP7 |
| Small Molecule Biochemistry | 1.6E-04-3.76E-03 | 20 | CCR1, CACNA1D, SMAD3, CLU, SLC18A3, SLC22A7, TRPV1, IL6, CCL11, NR3C1, APP, GABBR2, CUX1, PEX5L, CRP, ZBTB20, IRS2, BMP7, SERPINE1, CCL1 |
| Amino Acid Metabolism | 2.06E-04-2.06E-04 | 5 | GABBR2, IL6, TRPV1, SLC22A7, APP |
| Cell Cycle | 3.07E-04-3.76E-03 | 21 | GLI2, MAPK1, TP73, SMAD3, CLU, SYCP3, IL6, DCLK1, NR3C1, EIF4E, MECOM, APP, VEGFA, RUNX1T1, MYF6, ABTB1, PIM1, TSC2, BMP7, PRKCH, IRS2 |
| Protein Synthesis | 4.06E-04-1.99E-03 | 12 | VEGFA, MAPK1, TP73, PIM1, TSC2, RPS6KB2, IL6, ELAVL4, RELN, KLF2, NR3C1, EIF4E |
| Free Radical Scavenging | 6.62E-04-3.97E-03 | 12 | CCL2, MAPK1, RND3, PIM1, SMAD3, CRP, TSC2, RPS6KB2, IL6, CCL11, CCL1, APP |
| Drug Metabolism | 1.05E-03-1.2E-03 | 4 | BMP7, IRS2, IL6, APP |
| Lipid Metabolism | 1.2E-03-3.73E-03 | 11 | CUX1, PEX5L, SMAD3, CRP, CLU, BMP7, IRS2, IL6, SERPINE1, NR3C1, APP |
| DNA Replication, Recombination, and Repair | 1.25E-03-1.25E-03 | 11 | VEGFA, GLI2, TP73, TSC2, CLU, PRKCH, BMP7, IRS2, IL6, KLF2, APP |
| Cellular Compromise | 1.56E-03-4.04E-03 | 9 | VEGFA, POU4F2, CACNA1D, MAPK1, TP73, SMAD3, CLU, IL6, APP |
| Carbohydrate Metabolism | 3.76E-03-3.76E-03 | 4 | ZBTB20, IRS2, IL6, APP |

### Supplementary Table 17. Diseases and disorders significantly associated with the altered rapamycin response elements in the frontal lobe of advanced AD patients (neocortical stage) compared to control (176 genes)

| **Category** | **p-value** | **No. of genes** | **Molecules** |
| --- | --- | --- | --- |
| Renal and Urological Disease | 8.74E-07-2.63E-03 | 16 | CCR1, CACNA1D, MAPK1, SMAD3, CLU, TRPV1, IL6, NR3C1, VEGFA, CCL2, TSC2, CRP, PRKCH, IRS2, BMP7, SERPINE1 |
| Cardiovascular Disease | 9.97E-07-3.46E-03 | 23 | CCR1, CACNA1D, MAPK1, SMAD3, DLG2, CLU, IL6, NR3C1, MECOM, SERPIND1, APP, VEGFA, CCL2, PIM1, TSC2, CRP, RPS6KB2, IRS2, BMP7, PRKCH, VSNL1, SERPINE1, KLF2 |
| Connective Tissue Disorders | 3.9E-06-2.11E-03 | 13 | TP73, SMAD3, MMP16, CLU, IL6, NR3C1, ADAMTS2, SCN8A, VEGFA, CCL2, LUM, BMP7, SERPINE1 |
| Inflammatory Disease | 3.9E-06-3.46E-03 | 19 | TP73, MMP16, SMAD3, CLU, IL6, TRPV1, CCL11, NR3C1, ADAMTS2, SCN8A, APP, VEGFA, CUX1, CCL2, LUM, CRP, BPI, SERPINE1, KLF2 |
| Skeletal and Muscular Disorders | 3.9E-06-2.11E-03 | 14 | TP73, SMAD3, MMP16, CLU, IL6, NR3C1, ADAMTS2, SCN8A, APP, VEGFA, CCL2, LUM, BMP7, SERPINE1 |
| Inflammatory Response | 1.01E-05-3.8E-03 | 28 | GLI2, ITGBL1, CEACAM6, MAPK1, TP73, SMAD3, CLU, IL6, TRPV1, NR3C1, VEGFA, CCL2, PIM1, LUM, TSC2, RNF128, GFAP, SERPINE1, KLF2, CCL1, CCR1, CCL11, HDAC5, MECOM, APP, CUX1, CRP, BPI |
| Cancer | 1.07E-05-3.73E-03 | 60 | HCAR3, GLI2, MAPK1, TP73, SMAD3, CCNB2, IL6, ADAMTS2, HOXD10, VEGFA, PIM1, TSC2, LUM, ZBTB20, IRS2, GFAP, SERPINE1, HYDIN, KLF2, SLITRK1, MFAP5, HIST1H4A, SEMA5A, DCD, CREB3L4, MECOM, HDAC5, RUNX1T1, RND3, PRKCH, CEACAM6, ITGBL1, ATRNL1, PVRL3, CLU, TAOK1, TRPV1, DCLK1, NR3C1, SHE, EIF4E, SERPIND1, CCL2, RPS6KB2, SORBS2, CCR1, CACNA1D, MGC24103, MOXD1, SLC18A3, CCL11, RELN, APP, CUX1, GABBR2, CRP, SLC22A18, BMP7, NTSR1, KCNG4 |
| Neurological Disease | 1.07E-05-3.94E-03 | 37 | ATAD3A/ATAD3B, GLI2, TP73, DLG2, CLU, CCNB2, TAOK1, IL6, HMGCS2, NR3C1, HOXD10, SCN8A, VEGFA, CCL2, KCNAB1, CPLX2, MCTP1, TSC2, LUM, GFAP, EBF2, SERPINE1, SLITRK1, CACNA1D, SEMA5A, CEP68, SLC18A3, RELN, PRODH2, HDAC5, APP, AP1S1, GABBR2, STAG3L1, GLRA1, CRP, VSNL1 |
| Ophthalmic Disease | 1.08E-05-3.73E-03 | 6 | VEGFA, LUM, CRP, GFAP, IL6, NR3C1 |
| Organismal Injury and Abnormalities | 1.82E-05-3.07E-03 | 21 | CCR1, CACNA1D, GLI2, TP73, SMAD3, CLU, TRPV1, IL6, NR3C1, DYNC2LI1, APP, MECOM, SERPIND1, VEGFA, CCL2, CRP, LUM, PRKCH, BMP7, SERPINE1, KLF2 |
| Infectious Disease | 2.91E-05-4.04E-03 | 36 | ATAD3A/ATAD3B, CEACAM6, MAPK1, TP73, SMAD3, CLU, DLG2, CARD14, TRPV1, IL6, TAOK1, DCLK1, NR3C1, TIMM17B, VEGFA, CCL2, LUM, IRS2, SERPINE1, KLF2, NCKAP1, CCR1, DCD, CEP68, CCL11, MECOM, APP, AP1S1, GABBR2, CUX1, SERPINA10, ABTB1, STAG3L1, CRP, PRKCH, BPI |
| Auditory Disease | 7.13E-05-7.13E-05 | 2 | VEGFA, KLF2 |
| Dermatological Diseases and Conditions | 7.13E-05-2.47E-03 | 7 | VEGFA, SMAD3, CLU, PRKCH, TRPV1, NR3C1, SERPIND1 |
| Gastrointestinal Disease | 7.13E-05-3.73E-03 | 21 | CCR1, GLI2, HIST1H4A, TP73, SMAD3, PVRL3, CLU, CCNB2, TRPV1, IL6, NR3C1, APP, VEGFA, CCL2, TSC2, CRP, BPI, IRS2, BMP7, SERPINE1, SLITRK1 |
| Hepatic System Disease | 7.13E-05-2.04E-03 | 10 | CCR1, VEGFA, CCL2, TP73, SMAD3, BMP7, TRPV1, IL6, SERPINE1, NR3C1 |
| Respiratory Disease | 7.13E-05-3.07E-03 | 14 | TP73, SMAD3, CLU, IL6, TRPV1, CCL11, NR3C1, VEGFA, CUX1, CCL2, CRP, IRS2, BPI, SERPINE1 |
| Developmental Disorder | 1.27E-04-3.73E-03 | 23 | KCND2, POU4F2, GLI2, MAPK1, SMAD3, MMP16, ACVR2B, IL6, RELN, NR3C1, MECOM, HDAC5, APP, VEGFA, CUX1, MYF6, IRX4, PIM1, TSC2, EBF2, BMP7, GFAP, SERPINE1 |
| Hematological Disease | 1.96E-04-1.05E-03 | 7 | VEGFA, CRP, IL6, SERPINE1, NR3C1, SERPIND1, APP |
| Hypersensitivity Response | 2.02E-04-3.07E-03 | 3 | VEGFA, CCL2, CCL11 |
| Endocrine System Disorders | 4.27E-04-3.42E-03 | 16 | CEACAM6, TP73, MFAP5, CLU, CCNB2, IL6, NR3C1, APP, MECOM, VEGFA, CCL2, CRP, TSC2, BMP7, IRS2, SERPINE1 |
| Reproductive System Disease | 4.27E-04-2.94E-03 | 27 | GLI2, CEACAM6, TP73, CLU, CCNB2, IL6, TRPV1, NR3C1, HOXD10, EIF4E, VEGFA, CCL2, PIM1, TSC2, LUM, IRS2, SERPINE1, KLF2, MGC24103, MFAP5, MOXD1, MECOM, CUX1, RUNX1T1, CRP, BMP7, PRKCH |
| Immunological Disease | 1.34E-03-2.47E-03 | 4 | CRP, CLU, IL6, NR3C1 |
| Metabolic Disease | 1.88E-03-3.42E-03 | 9 | CCL2, CRP, TSC2, CLU, BMP7, IRS2, IL6, SERPINE1, APP |
| Psychological Disorders | 3.94E-03-3.94E-03 | 11 | GABBR2, CACNA1D, CCL2, MCTP1, CEP68, GFAP, RELN, HMGCS2, NR3C1, PRODH2, APP |

### Supplementary Table 18. Physiological system development and function significantly associated with the altered rapamycin response elements in the frontal lobe of advanced AD patients (neocortical stage) compared to control (176 genes)

| **Category** | **p-value** | **No. of genes** | **Molecules** |
| --- | --- | --- | --- |
| Nervous System Development and Function | 2.39E-07-4.13E-03 | 47 | KCND2, GLI2, MAPK1, TP73, DRP2, FAT3, DLG2, ELAVL4, TRPV1, IL6, DCLK1, NR3C1, HOXD10, SEMA4C, DYNC2LI1, SCN8A, VEGFA, CTDSP1, KCNAB1, CCL2, CPLX2, TSC2, RPS6KB2, ZBTB20, GFAP, EBF2, IRS2, SERPINE1, NCKAP1, CCR1, CACNA1D, POU4F2, SEMA5A, ACSL6, SLC18A3, GPR176, RELN, MECOM, APP, CUX1, GABBR2, GLRA1, BMP7, PRKCH, IL1RAPL1, VSNL1, NTSR1 |
| Embryonic Development | 9.98E-07-4.04E-03 | 47 | GLI2, MAPK1, TP73, MMP16, SMAD3, PVRL3, CLU, FAT3, TRPV1, ACVR2B, IL6, DCLK1, NR3C1, SEMA4C, ADAMTS2, EIF4E, DYNC2LI1, HOXD10, SERPIND1, VEGFA, MYF6, CCL2, LECT1, TSC2, LUM, ZBTB20, EBF2, IRS2, SERPINE1, HYDIN, KLF2, NCKAP1, CCL1, CCR1, POU4F2, BCOR, SEMA5A, ACSL6, RELN, MECOM, HDAC5, APP, CUX1, IRX4, BMP7, BPI, IL1RAPL1 |
| Organismal Development | 9.98E-07-4.04E-03 | 54 | GLI2, MAPK1, TP73, SMAD3, MMP16, CCNB2, IL6, HOXD10, ADAMTS2, VEGFA, LECT1, PIM1, TSC2, LUM, ZBTB20, GFAP, IRS2, EBF2, SERPINE1, SLITRK1, HYDIN, KLF2, BCOR, SEMA5A, HDAC5, MECOM, IRX4, RAB3C, BPI, PRKCH, IL1RAPL1, PVRL3, FAT3, CLU, TRPV1, ACVR2B, DCLK1, NR3C1, DYNC2LI1, EIF4E, CCL2, MYF6, STARD13, CCL1, NCKAP1, CCR1, POU4F2, CACNA1D, RELN, CCL11, APP, CUX1, GABBR2, BMP7 |
| Tissue Development | 9.98E-07-4.04E-03 | 52 | GLI2, MAPK1, TP73, SMAD3, MMP16, IL6, ADAMTS2, HOXD10, VEGFA, LECT1, PIM1, LUM, TSC2, ZBTB20, GFAP, EBF2, IRS2, SERPINE1, HYDIN, KLF2, BCOR, SEMA5A, MECOM, HDAC5, RND3, IRX4, BPI, IL1RAPL1, VSNL1, PVRL3, FAT3, CLU, TRPV1, ACVR2B, DCLK1, NR3C1, EIF4E, DYNC2LI1, SERPIND1, CCL2, MYF6, STARD13, NCKAP1, CCL1, CCR1, POU4F2, RELN, CCL11, APP, CUX1, CRP, BMP7 |
| Cardiovascular System Development and Function | 2.3E-06-4.08E-03 | 32 | GLI2, MAPK1, TP73, SMAD3, CLU, IL6, ACVR2B, NR3C1, ADAMTS2, HOXD10, VEGFA, CCL2, PIM1, LECT1, LUM, TSC2, RPS6KB2, GFAP, SERPINE1, KLF2, CCL1, CCR1, BCOR, SEMA5A, CCL11, APP, MECOM, HDAC5, IRX4, CRP, BMP7, BPI |
| Respiratory System Development and Function | 6.43E-06-1.5E-03 | 14 | GLI2, MMP16, ACVR2B, IL6, NR3C1, ADAMTS2, VEGFA, CUX1, CCL2, EBF2, BMP7, SERPINE1, HYDIN, KLF2 |
| Hematological System Development and Function | 1.01E-05-4.13E-03 | 28 | MAPK1, SMAD3, CLU, IL6, TRPV1, NR3C1, EIF4E, SERPIND1, VEGFA, CCL2, PIM1, LUM, TSC2, RNF128, GFAP, SERPINE1, KLF2, CCL1, CCR1, HIST1H4A, CCL11, APP, MECOM, HDAC5, CUX1, RUNX1T1, CRP, BPI |
| Immune Cell Trafficking | 1.01E-05-3.8E-03 | 16 | CCR1, SMAD3, CLU, IL6, TRPV1, CCL11, APP, VEGFA, CUX1, CCL2, CRP, LUM, GFAP, SERPINE1, KLF2, CCL1 |
| Skeletal and Muscular System Development and Function | 2.69E-05-4.08E-03 | 28 | GLI2, MAPK1, TP73, MMP16, SMAD3, CLU, IL6, ACVR2B, NR3C1, SEMA4C, HOXD10, DYNC2LI1, BARX2, VEGFA, MYF6, CCL2, PIM1, LECT1, LUM, SERPINE1, KLF2, CCL1, CCR1, HDAC5, MECOM, APP, IRX4, BMP7 |
| Tissue Morphology | 2.69E-05-4.08E-03 | 40 | KCND2, GLI2, MAPK1, TP73, SMAD3, MMP16, FAT3, CLU, CCNB2, IL6, DCLK1, NR3C1, HOXD10, DYNC2LI1, SERPIND1, VEGFA, MYF6, CCL2, PIM1, TSC2, LUM, RPS6KB2, IRS2, GFAP, EBF2, SERPINE1, KLF2, CCR1, POU4F2, CACNA1D, SEMA5A, SLC18A3, CREB3L4, CCL11, MECOM, APP, CUX1, CRP, PRKCH, BMP7 |
| Connective Tissue Development and Function | 3.23E-05-3.58E-03 | 22 | GLI2, MAPK1, TP73, MMP16, SMAD3, ACVR2B, IL6, NR3C1, HOXD10, MECOM, HDAC5, VEGFA, RUNX1T1, RND3, CCL2, MYF6, LECT1, LUM, SLC25A27, IRS2, BMP7, KLF2 |
| Hepatic System Development and Function | 3.23E-05-3.23E-05 | 3 | MAPK1, CCL2, SMAD3 |
| Hematopoiesis | 3.85E-05-1.84E-03 | 18 | CCR1, HIST1H4A, SMAD3, IL6, CCL11, EIF4E, HDAC5, MECOM, APP, VEGFA, CUX1, RUNX1T1, CCL2, TSC2, RNF128, BMP7, KLF2, CCL1 |
| Lymphoid Tissue Structure and Development | 6.93E-05-3.73E-03 | 11 | VEGFA, CCR1, RUNX1T1, CCL2, PIM1, SMAD3, RNF128, BMP7, IL6, APP, CCL1 |
| Hair and Skin Development and Function | 7.13E-05-1.47E-03 | 11 | VEGFA, GLI2, SMAD3, LUM, CLU, PRKCH, IL6, TRPV1, NR3C1, ADAMTS2, BARX2 |
| Organ Development | 9.51E-05-4.04E-03 | 39 | GLI2, TP73, SMAD3, MMP16, PVRL3, FAT3, CLU, IL6, ACVR2B, TRPV1, DCLK1, NR3C1, DYNC2LI1, ADAMTS2, HOXD10, VEGFA, MYF6, CCL2, LECT1, TSC2, LUM, ZBTB20, IRS2, EBF2, SERPINE1, HYDIN, KLF2, CCL1, CCR1, POU4F2, BCOR, SEMA5A, RELN, HDAC5, MECOM, CUX1, IRX4, BMP7, IL1RAPL1 |
| Digestive System Development and Function | 9.99E-05-3.33E-03 | 16 | CCR1, GLI2, CACNA1D, BCOR, TP73, SMAD3, PVRL3, CLU, ACVR2B, IL6, ADAMTS2, MECOM, APP, VEGFA, BMP7, IRS2 |
| Organ Morphology | 1.14E-04-4.08E-03 | 25 | GLI2, MAPK1, TP73, SMAD3, MMP16, FAT3, CLU, IL6, ACVR2B, NR3C1, HOXD10, ADAMTS2, VEGFA, MYF6, CCL2, LECT1, LUM, IRS2, EBF2, KLF2, CACNA1D, SEMA5A, CUX1, IRX4, BMP7 |
| Behavior | 1.31E-04-3.74E-03 | 18 | NAV2, MAPK1, SLC18A3, IL6, TRPV1, RELN, NR3C1, HOXD10, SCN8A, APP, VEGFA, GABBR2, KCNAB1, CPLX2, GLRA1, RAB3C, SLITRK1, NTSR1 |
| Visual System Development and Function | 3.22E-04-4.04E-03 | 11 | VEGFA, POU4F2, SEMA5A, LUM, PVRL3, FAT3, BMP7, EBF2, SERPINE1, NR3C1, CCL1 |
| Reproductive System Development and Function | 6.1E-04-2.5E-03 | 7 | TP73, SMAD3, CLU, EBF2, BMP7, IRS2, IL6 |
| Auditory and Vestibular System Development and Function | 1.05E-03-1.05E-03 | 2 | VEGFA, KLF2 |
| Cell-mediated Immune Response | 1.05E-03-3.73E-03 | 9 | CCR1, CCL2, SMAD3, RNF128, IL6, CCL11, KLF2, APP, CCL1 |
| Organismal Functions | 1.05E-03-3.07E-03 | 8 | VEGFA, MAPK1, SMAD3, LUM, PRKCH, IL6, SERPINE1, NR3C1 |
| Endocrine System Development and Function | 1.2E-03-2.69E-03 | 8 | TSC2, CLU, BMP7, IRS2, ACVR2B, IL6, NR3C1, APP |
| Tumor Morphology | 1.99E-03-2.9E-03 | 14 | TP73, SMAD3, CLU, IL6, CCL11, NR3C1, APP, VEGFA, PIM1, TSC2, LUM, TNFRSF10C, BMP7, SERPINE1 |
| Organismal Survival | 3.01E-03-3.01E-03 | 27 | MAPK1, TP73, SMAD3, SYCP3, ACVR2B, IL6, NR3C1, SERPIND1, VEGFA, MYF6, KCNAB1, TSC2, RPS6KB2, ZBTB20, IRS2, GFAP, SERPINE1, KLF2, CCR1, SLC18A3, CCL11, APP, GABBR2, CUX1, SERPINA10, CRP, RAB3C |
| Humoral Immune Response | 3.07E-03-3.07E-03 | 2 | SMAD3, IL6 |

### Supplementary Table 19. Networks significantly associated with the altered rapamycin response elements in the frontal lobe of advanced AD patients (neocortical stage) compared to control (176 genes)

| **ID** | **Top Functions** | **Score** | **Focus Molecules** |
| --- | --- | --- | --- |
| 1 | Endocrine System Disorders, Gastrointestinal Disease, Hepatic System Disease | 36 | 19 |
| 2 | Cancer, Hematological Disease, Connective Tissue Disorders | 35 | 19 |
| 3 | Connective Tissue Development and Function, Tissue Morphology, Cell Cycle | 31 | 17 |
| 4 | Developmental Disorder, Hereditary Disorder, Neurological Disease | 29 | 16 |
| 5 | Cell-To-Cell Signaling and Interaction, Hematological System Development and Function, Inflammatory Response | 24 | 14 |
| 6 | Drug Metabolism, Skeletal and Muscular System Development and Function, Carbohydrate Metabolism | 24 | 14 |
| 7 | Energy Production, Lipid Metabolism, Small Molecule Biochemistry | 24 | 14 |
| 8 | Cell-To-Cell Signaling and Interaction, Nervous System Development and Function, Cardiovascular System Development and Function | 17 | 11 |
| 9 | Embryonic Development, Organismal Development, Tissue Development | 16 | 10 |
| 10 | Protein Synthesis, Cancer, Reproductive System Disease | 14 | 10 |
| 11 | Hair and Skin Development and Function, Connective Tissue Development and Function, Skeletal and Muscular System Development and Function | 13 | 9 |
| 12 | Cell-To-Cell Signaling and Interaction, Skeletal and Muscular System Development and Function, Cellular Development | 10 | 7 |

### Supplementary Table 20. Pathways significantly associated with the altered rapamycin response elements in the frontal lobe of advanced AD patients (neocortical stage) compared to control (176 genes)

| **Ingenuity Canonical Pathways** | **p-value** | **Ratio (No. of significant genes / total number in pathway)** |
| --- | --- | --- |
| IL-17 Signaling | 0.000 | 0.068 |
| Role of IL-17F in Allergic Inflammatory Airway Diseases | 0.000 | 0.083 |
| TGF-β Signaling | 0.001 | 0.056 |
| mTOR Signaling | 0.001 | 0.038 |
| 14-3-3-mediated Signaling | 0.003 | 0.043 |
| Corticotropin Releasing Hormone Signaling | 0.003 | 0.037 |
| IL-10 Signaling | 0.003 | 0.051 |
| Acute Phase Response Signaling | 0.003 | 0.034 |
| IL-6 Signaling | 0.003 | 0.040 |
| IL-17A Signaling in Fibroblasts | 0.003 | 0.075 |
| Insulin Receptor Signaling | 0.005 | 0.035 |
| Role of Hypercytokinemia/hyperchemokinemia in the Pathogenesis of Influenza | 0.005 | 0.068 |
| Axonal Guidance Signaling | 0.005 | 0.021 |
| Glucocorticoid Receptor Signaling | 0.006 | 0.024 |
| Bladder Cancer Signaling | 0.006 | 0.044 |
| Hepatic Fibrosis / Hepatic Stellate Cell Activation | 0.007 | 0.034 |
| HMGB1 Signaling | 0.008 | 0.040 |
| Chronic Myeloid Leukemia Signaling | 0.008 | 0.038 |
| Differential Regulation of Cytokine Production in Macrophages and T Helper Cells by IL-17A and IL-17F | 0.010 | 0.111 |
| HIF1α Signaling | 0.011 | 0.037 |
| Thrombopoietin Signaling | 0.011 | 0.048 |
| TREM1 Signaling | 0.014 | 0.042 |
| ATM Signaling | 0.014 | 0.049 |
| Role of Macrophages, Fibroblasts and Endothelial Cells in Rheumatoid Arthritis | 0.014 | 0.021 |
| PPARα/RXRα Activation | 0.014 | 0.027 |
| Pyridoxal 5'-phosphate Salvage Pathway | 0.014 | 0.042 |
| Colorectal Cancer Metastasis Signaling | 0.015 | 0.023 |
| IL-17A Signaling in Airway Cells | 0.016 | 0.042 |
| Type II Diabetes Mellitus Signaling | 0.017 | 0.025 |
| Atherosclerosis Signaling | 0.017 | 0.029 |
| Role of JAK family kinases in IL-6-type Cytokine Signaling | 0.019 | 0.074 |
| PXR/RXR Activation | 0.019 | 0.035 |
| ILK Signaling | 0.019 | 0.026 |
| LXR/RXR Activation | 0.019 | 0.029 |
| PI3K/AKT Signaling | 0.019 | 0.028 |
| Antiproliferative Role of TOB in T Cell Signaling | 0.020 | 0.077 |
| Growth Hormone Signaling | 0.021 | 0.040 |
| Chemokine Signaling | 0.021 | 0.041 |
| FLT3 Signaling in Hematopoietic Progenitor Cells | 0.022 | 0.040 |
| Prolactin Signaling | 0.024 | 0.038 |
| VEGF Family Ligand-Receptor Interactions | 0.024 | 0.036 |
| MIF-mediated Glucocorticoid Regulation | 0.026 | 0.048 |
| Acute Myeloid Leukemia Signaling | 0.027 | 0.037 |
| Salvage Pathways of Pyrimidine Ribonucleotides | 0.030 | 0.029 |
| Hepatic Cholestasis | 0.030 | 0.023 |
| Huntington's Disease Signaling | 0.032 | 0.021 |
| Proline Biosynthesis I | 0.033 | 0.071 |
| Coagulation System | 0.034 | 0.053 |
| Inhibition of Angiogenesis by TSP1 | 0.034 | 0.051 |
| Melanocyte Development and Pigmentation Signaling | 0.035 | 0.033 |
| Factors Promoting Cardiogenesis in Vertebrates | 0.036 | 0.032 |
| FGF Signaling | 0.036 | 0.033 |
| Neuregulin Signaling | 0.038 | 0.029 |
| Cardiac Hypertrophy Signaling | 0.039 | 0.021 |
| Fcγ Receptor-mediated Phagocytosis in Macrophages and Monocytes | 0.040 | 0.039 |
| Proline Biosynthesis II (from Arginine) | 0.042 | 0.050 |
| Phospholipase C Signaling | 0.042 | 0.023 |
| Role of Pattern Recognition Receptors in Recognition of Bacteria and Viruses | 0.046 | 0.028 |
| IGF-1 Signaling | 0.048 | 0.029 |

### Supplementary Table 21. Molecular and cellular function significantly associated with the rapamycin-regulated genes identified as differentially expressed in the frontal lobe as a result of AD, irrespective of disease severity, as determined by multiclass analysis (55 genes)

| **Category** | **p-value** | **No. of genes** | **Molecules** |
| --- | --- | --- | --- |
| Cell Cycle | 6.33E-04-4.61E-02 | 7 | RUNX1T1, MYF6, MAPK1, IRS2, PRKCH, BMP7, HDAC5 |
| Cellular Development | 6.33E-04-4.9E-02 | 13 | MAPK1, DCD, ELAVL4, ACVR2B, SEMA4C, HDAC5, RUNX1T1, MYF6, RPS6KB2, EGLN3, BMP7, PRKCH, IRS2 |
| Cell Death and Survival | 1.2E-03-4.61E-02 | 12 | RUNX1T1, MAPK1, DCD, RPS6KB2, EGLN3, SLC18A3, IRS2, FN3K, BMP7, PRKCH, ACVR2B, HDAC5 |
| Cell-To-Cell Signaling and Interaction | 2.06E-03-2.56E-02 | 8 | GABBR2, MAPK1, DLG2, SLC18A3, EGLN3, BMP7, SLITRK1, SCN8A |
| Cellular Growth and Proliferation | 2.3E-03-4.45E-02 | 11 | LOXL4, RUNX1T1, MYF6, MAPK1, RPS6KB2, DCD, BMP7, PRKCH, IRS2, SEMA4C, HDAC5 |
| Cell Morphology | 2.35E-03-3.48E-02 | 7 | MAPK1, SLC18A3, EGLN3, PRKCH, BMP7, IRS2, ADAMTS2 |
| Cellular Function and Maintenance | 2.35E-03-4.16E-02 | 7 | RUNX1T1, STX1B, SLC18A3, RAB3C, BMP7, PRKCH, HDAC5 |
| Molecular Transport | 2.35E-03-4.83E-02 | 12 | MAPK1, STX1B, RPS6KB2, SLC22A18, SLC18A3, RAB3C, EGLN3, PRKCH, BMP7, IRS2, SLITRK1, SCN8A |
| Post-Translational Modification | 3.51E-03-1.64E-02 | 8 | MAPK1, MAPK4, MMP16, RPS6KB2, PRKCH, BMP7, FN3K, ACVR2B |
| Carbohydrate Metabolism | 4.7E-03-4.83E-02 | 5 | MAPK1, RPS6KB2, BMP7, FN3K, IRS2 |
| Cellular Assembly and Organization | 4.7E-03-4.16E-02 | 5 | GABBR2, SLC18A3, RAB3C, ADAMTS2, HDAC5 |
| Gene Expression | 4.7E-03-3.25E-02 | 2 | BMP7, HDAC5 |
| Nucleic Acid Metabolism | 4.7E-03-4.16E-02 | 4 | ENTPD8, MAPK1, RPS6KB2, BMP7 |
| Small Molecule Biochemistry | 4.7E-03-4.83E-02 | 9 | ENTPD8, MAPK1, RPS6KB2, SLC18A3, EGLN3, FN3K, BMP7, IRS2, SLITRK1 |
| Protein Trafficking | 7.05E-03-7.05E-03 | 1 | MAPK1 |
| Drug Metabolism | 7.4E-03-4.83E-02 | 7 | MAPK1, RPS6KB2, SLC22A18, EGLN3, BMP7, IRS2, SLITRK1 |
| Lipid Metabolism | 7.4E-03-4.83E-02 | 3 | MAPK1, BMP7, IRS2 |
| Cellular Movement | 8.12E-03-2.33E-02 | 6 | MAPK1, MMP16, BMP7, IRS2, SEMA4C, SCN8A |
| Cell Signaling | 9.39E-03-3.93E-02 | 6 | MAPK1, MMP16, DLG2, SLC18A3, BMP7, ACVR2B |
| Vitamin and Mineral Metabolism | 9.39E-03-1.87E-02 | 4 | MAPK1, DLG2, SLC18A3, BMP7 |
| Cellular Compromise | 1.17E-02-1.17E-02 | 1 | MAPK1 |
| Protein Degradation | 1.64E-02-1.87E-02 | 1 | MMP16 |
| Protein Synthesis | 1.64E-02-3.25E-02 | 4 | MMP16, RPS6KB2, EGLN3, IRS2 |
| Energy Production | 3.02E-02-3.02E-02 | 1 | MAPK1 |
| DNA Replication, Recombination, and Repair | 3.25E-02-4.38E-02 | 2 | PRKCH, HDAC5 |

### Supplementary Table 22. Diseases and disorders significantly associated with the rapamycin-regulated genes identified as differentially expressed in the frontal lobe as a result of AD, irrespective of disease severity, as determined by multiclass analysis (55 genes)

| **Category** | **p-value** | **No. of genes** | **Molecules** |
| --- | --- | --- | --- |
| Developmental Disorder | 8.03E-05-4.61E-02 | 8 | MYF6, MAPK1, MMP16, CC2D2A, BMP7, ACVR2B, ADAMTS2, HDAC5 |
| Renal and Urological Disease | 2.25E-03-3.64E-02 | 4 | LOXL4, MAPK1, BMP7, ACVR2B |
| Connective Tissue Disorders | 2.35E-03-4.61E-02 | 6 | MMP16, BMP7, ACVR2B, ADAMTS2, SCN8A, HDAC5 |
| Hereditary Disorder | 2.35E-03-3.7E-02 | 8 | MYF6, BMP7, CC2D2A, ACVR2B, SLITRK1, ADAMTS2, SCN8A, HDAC5 |
| Neurological Disease | 2.35E-03-3.74E-02 | 7 | GABBR2, DLG2, BMP7, CC2D2A, PRKCH, SLITRK1, SCN8A |
| Ophthalmic Disease | 2.35E-03-3.25E-02 | 1 | BMP7 |
| Skeletal and Muscular Disorders | 2.35E-03-3.54E-02 | 9 | MAPK1, MYF6, MMP16, CC2D2A, BMP7, ACVR2B, ADAMTS2, SCN8A, HDAC5 |
| Dermatological Diseases and Conditions | 3.64E-03-4.7E-03 | 4 | RPS6KB2, RAB3C, ACVR2B, ADAMTS2 |
| Hematological Disease | 3.64E-03-4.83E-02 | 5 | RUNX1T1, RPS6KB2, RAB3C, BMP7, ACVR2B |
| Cancer | 4.7E-03-4.83E-02 | 9 | LOXL4, RUNX1T1, MAPK1, ATRNL1, SLC18A3, EGLN3, IRS2, BMP7, ACVR2B |
| Cardiovascular Disease | 4.7E-03-3.74E-02 | 5 | MAPK1, DLG2, BMP7, PRKCH, ACVR2B |
| Metabolic Disease | 4.7E-03-4.38E-02 | 3 | IRS2, ADAMTS2, HDAC5 |
| Nutritional Disease | 4.7E-03-1.44E-02 | 3 | BMP7, IRS2, HDAC5 |
| Organismal Injury and Abnormalities | 4.7E-03-2.33E-02 | 5 | MAPK1, BMP7, ACVR2B, ADAMTS2, HDAC5 |
| Inflammatory Disease | 4.73E-03-4.73E-03 | 3 | MMP16, ADAMTS2, SCN8A |
| Gastrointestinal Disease | 7.05E-03-3.02E-02 | 4 | MMP16, EGLN3, IRS2, ACVR2B |
| Psychological Disorders | 1.17E-02-1.17E-02 | 1 | GABBR2 |
| Reproductive System Disease | 1.17E-02-3.02E-02 | 2 | BMP7, IRS2 |
| Hepatic System Disease | 1.5E-02-1.5E-02 | 3 | EGLN3, IRS2, ACVR2B |
| Respiratory Disease | 1.87E-02-3.85E-02 | 3 | MYF6, RAB3C, ACVR2B |
| Endocrine System Disorders | 2.33E-02-3.02E-02 | 1 | IRS2 |

### Supplementary Table 23. Physiological system development and function significantly associated with the rapamycin-regulated genes identified as differentially expressed in the frontal lobe as a result of AD, irrespective of disease severity, as determined by multiclass analysis (55 genes)

| **Category** | **p-value** | **No. of genes** | **Molecules** |
| --- | --- | --- | --- |
| Respiratory System Development and Function | 6.48E-05-4.56E-02 | 5 | MMP16, EGLN3, BMP7, ACVR2B, ADAMTS2 |
| Digestive System Development and Function | 2.4E-04-2.56E-02 | 5 | EGLN3, BMP7, IRS2, ACVR2B, ADAMTS2 |
| Organismal Development | 2.4E-04-4.83E-02 | 13 | MAPK1, MMP16, ACVR2B, ADAMTS2, HDAC5, GABBR2, MYF6, EGLN3, RAB3C, BMP7, IRS2, PRKCH, SLITRK1 |
| Skeletal and Muscular System Development and Function | 2.58E-04-4.83E-02 | 9 | MAPK1, MYF6, MMP16, EGLN3, SLC18A3, BMP7, ACVR2B, SEMA4C, HDAC5 |
| Organ Morphology | 6.33E-04-4.83E-02 | 8 | MYF6, MAPK1, MMP16, EGLN3, BMP7, IRS2, ACVR2B, ADAMTS2 |
| Tissue Development | 6.33E-04-4.83E-02 | 9 | MAPK1, MYF6, MMP16, EGLN3, SLC18A3, IRS2, BMP7, ACVR2B, HDAC5 |
| Connective Tissue Development and Function | 6.91E-04-4.9E-02 | 9 | RUNX1T1, MAPK1, MYF6, MMP16, EGLN3, BMP7, IRS2, ACVR2B, HDAC5 |
| Embryonic Development | 6.91E-04-4.83E-02 | 9 | MAPK1, MYF6, MMP16, EGLN3, IRS2, BMP7, ACVR2B, SEMA4C, HDAC5 |
| Tissue Morphology | 1.21E-03-4.83E-02 | 9 | MAPK1, MYF6, MMP16, EGLN3, BMP7, IRS2, ACVR2B, ADAMTS2, HDAC5 |
| Organ Development | 1.69E-03-4.83E-02 | 9 | MAPK1, MYF6, MMP16, SLC18A3, EGLN3, IRS2, BMP7, ACVR2B, HDAC5 |
| Behavior | 1.88E-03-2.79E-02 | 4 | GABBR2, MAPK1, RAB3C, SLITRK1 |
| Nervous System Development and Function | 2.06E-03-4.73E-02 | 12 | GABBR2, MAPK1, DLG2, RPS6KB2, EGLN3, SLC18A3, IRS2, BMP7, PRKCH, SEMA4C, SCN8A, HDAC5 |
| Hair and Skin Development and Function | 2.35E-03-3.93E-02 | 4 | MAPK1, BMP7, PRKCH, ADAMTS2 |
| Reproductive System Development and Function | 2.35E-03-4.38E-02 | 5 | MAPK1, STX1B, RAB3C, BMP7, IRS2 |
| Hematological System Development and Function | 4.7E-03-2.56E-02 | 2 | RUNX1T1, EGLN3 |
| Hematopoiesis | 4.7E-03-2.56E-02 | 1 | RUNX1T1 |
| Renal and Urological System Development and Function | 4.7E-03-4.56E-02 | 4 | MAPK1, IRS2, BMP7, ACVR2B |
| Visual System Development and Function | 7.05E-03-1.87E-02 | 2 | EGLN3, BMP7 |
| Endocrine System Development and Function | 7.4E-03-4.61E-02 | 5 | MAPK1, EGLN3, BMP7, IRS2, ACVR2B |
| Tumor Morphology | 9.39E-03-2.1E-02 | 3 | RUNX1T1, EGLN3, BMP7 |
| Organismal Functions | 1.4E-02-1.4E-02 | 1 | MAPK1 |
| Hepatic System Development and Function | 1.87E-02-1.87E-02 | 1 | MAPK1 |
| Cardiovascular System Development and Function | 2.33E-02-4.97E-02 | 8 | MAPK1, SLC18A3, EGLN3, IRS2, BMP7, ACVR2B, ADAMTS2, HDAC5 |
| Organismal Survival | 2.41E-02-2.41E-02 | 9 | GABBR2, MAPK1, MYF6, RPS6KB2, EGLN3, RAB3C, SLC18A3, IRS2, ACVR2B |
| Lymphoid Tissue Structure and Development | 2.9E-02-3.93E-02 | 2 | RUNX1T1, BMP7 |

### Supplementary Table 24. Networks significantly associated with the rapamycin-regulated genes identified as differentially expressed in the frontal lobe as a result of AD, irrespective of disease severity, as determined by multiclass analysis (55 genes)

| **ID** | **Top Functions** | **Score** | **Focus Molecules** |
| --- | --- | --- | --- |
| 1 | Cancer, Endocrine System Disorders, Reproductive System Disease | 42 | 17 |
| 2 | Cellular Development, Connective Tissue Development and Function, Embryonic Development | 35 | 15 |
| 3 | Developmental Disorder, Hereditary Disorder, Cellular Growth and Proliferation | 27 | 12 |

### Supplementary Table 25. Pathways significantly associated with the rapamycin-regulated genes identified as differentially expressed in the frontal lobe as a result of AD, irrespective of disease severity, as determined by multiclass analysis (55 genes)

| **Ingenuity Canonical Pathways** | **p-value** | **Ratio** |
| --- | --- | --- |
| HIF1α Signaling | 0.000 | 0.037 |
| Thrombopoietin Signaling | 0.000 | 0.048 |
| Growth Hormone Signaling | 0.001 | 0.040 |
| TGF-β Signaling | 0.001 | 0.034 |
| Factors Promoting Cardiogenesis in Vertebrates | 0.001 | 0.032 |
| Neuregulin Signaling | 0.001 | 0.029 |
| Bladder Cancer Signaling | 0.001 | 0.033 |
| Fcγ Receptor-mediated Phagocytosis in Macrophages and Monocytes | 0.001 | 0.039 |
| IGF-1 Signaling | 0.001 | 0.029 |
| Type II Diabetes Mellitus Signaling | 0.002 | 0.019 |
| Insulin Receptor Signaling | 0.003 | 0.021 |
| nNOS Signaling in Neurons | 0.005 | 0.039 |
| CNTF Signaling | 0.006 | 0.036 |
| Endothelin-1 Signaling | 0.007 | 0.021 |
| Leukocyte Extravasation Signaling | 0.008 | 0.015 |
| mTOR Signaling | 0.008 | 0.019 |
| Role of NFAT in Cardiac Hypertrophy | 0.008 | 0.015 |
| ErbB4 Signaling | 0.009 | 0.030 |
| Pyridoxal 5'-phosphate Salvage Pathway | 0.009 | 0.028 |
| Role of JAK1 and JAK3 in γc Cytokine Signaling | 0.009 | 0.030 |
| Erythropoietin Signaling | 0.011 | 0.026 |
| CCR5 Signaling in Macrophages | 0.011 | 0.021 |
| GDNF Family Ligand-Receptor Interactions | 0.011 | 0.027 |
| Renal Cell Carcinoma Signaling | 0.011 | 0.027 |
| Melatonin Signaling | 0.012 | 0.026 |
| IL-3 Signaling | 0.012 | 0.027 |
| BMP signaling pathway | 0.012 | 0.025 |
| Axonal Guidance Signaling | 0.012 | 0.009 |
| Huntington's Disease Signaling | 0.013 | 0.013 |
| LPS-stimulated MAPK Signaling | 0.013 | 0.024 |
| NF-κB Activation by Viruses | 0.013 | 0.024 |
| Prolactin Signaling | 0.013 | 0.025 |
| VEGF Family Ligand-Receptor Interactions | 0.013 | 0.024 |
| Acute Myeloid Leukemia Signaling | 0.014 | 0.024 |
| Salvage Pathways of Pyrimidine Ribonucleotides | 0.015 | 0.020 |
| Phospholipase C Signaling | 0.015 | 0.015 |
| Melanocyte Development and Pigmentation Signaling | 0.017 | 0.022 |
| α-Adrenergic Signaling | 0.017 | 0.019 |
| ErbB Signaling | 0.017 | 0.023 |
| FGF Signaling | 0.017 | 0.022 |
| G Beta Gamma Signaling | 0.018 | 0.017 |
| Role of Pattern Recognition Receptors in Recognition of Bacteria and Viruses | 0.020 | 0.019 |
| Chronic Myeloid Leukemia Signaling | 0.020 | 0.019 |
| Glioma Signaling | 0.021 | 0.018 |
| HGF Signaling | 0.023 | 0.019 |
| Neuropathic Pain Signaling In Dorsal Horn Neurons | 0.023 | 0.019 |
| Telomerase Signaling | 0.023 | 0.019 |
| Cholecystokinin/Gastrin-mediated Signaling | 0.023 | 0.019 |
| fMLP Signaling in Neutrophils | 0.025 | 0.016 |
| Natural Killer Cell Signaling | 0.026 | 0.017 |
| Fc Epsilon RI Signaling | 0.026 | 0.017 |
| NGF Signaling | 0.026 | 0.017 |
| Renin-Angiotensin Signaling | 0.027 | 0.016 |
| Role of NANOG in Mammalian Embryonic Stem Cell Pluripotency | 0.027 | 0.018 |
| Androgen Signaling | 0.027 | 0.014 |
| CCR3 Signaling in Eosinophils | 0.028 | 0.016 |
| 14-3-3-mediated Signaling | 0.028 | 0.017 |
| Corticotropin Releasing Hormone Signaling | 0.028 | 0.015 |
| PTEN Signaling | 0.030 | 0.015 |
| Synaptic Long Term Potentiation | 0.031 | 0.016 |
| P2Y Purigenic Receptor Signaling Pathway | 0.032 | 0.015 |
| p70S6K Signaling | 0.032 | 0.016 |
| PI3K/AKT Signaling | 0.033 | 0.014 |
| GNRH Signaling | 0.035 | 0.014 |
| PI3K Signaling in B Lymphocytes | 0.035 | 0.014 |
| AMPK Signaling | 0.037 | 0.012 |
| Parkinson's Signaling | 0.037 | 0.063 |
| Ovarian Cancer Signaling | 0.038 | 0.014 |
| IL-12 Signaling and Production in Macrophages | 0.039 | 0.013 |
| Synaptic Long Term Depression | 0.040 | 0.013 |
| Cardiomyocyte Differentiation via BMP Receptors | 0.042 | 0.050 |
| Aldosterone Signaling in Epithelial Cells | 0.043 | 0.012 |
| Molecular Mechanisms of Cancer | 0.044 | 0.008 |
| CXCR4 Signaling | 0.047 | 0.012 |
| B Cell Receptor Signaling | 0.049 | 0.012 |

### Supplementary Table 26. Genes known to be associated with Alzheimer’s disease, now identified as downstream effectors of mTOR.

| **Gene Symbol** | **Gene Name** | **Location** | **Type** |
| --- | --- | --- | --- |
| A2M | alpha-2-macroglobulin | Extracellular Space | transporter |
| AGT | angiotensinogen (serpin peptidase inhibitor, clade A, member 8)" | Extracellular Space | growth factor |
| AR | androgen receptor | Nucleus | ligand-dependent nuclear receptor |
| ARMC2 | armadillo repeat containing 2 | unknown | other |
| BCL2 | B-cell CLL/lymphoma 2 | Cytoplasm | transporter |
| CCL2 | chemokine (C-C motif) ligand 2 | Extracellular Space | cytokine |
| CLU | clusterin | Extracellular Space | other |
| CRP | C-reactive protein, pentraxin-related" | Extracellular Space | other |
| CTNNA3 | catenin (cadherin-associated protein), alpha 3" | Plasma Membrane | other |
| DCD | dermcidin | Extracellular Space | other |
| DCN | decorin | Extracellular Space | other |
| ESR1 | estrogen receptor 1 | Nucleus | ligand-dependent nuclear receptor |
| FGF2 | fibroblast growth factor 2 (basic) | Extracellular Space | growth factor |
| GABBR2 | gamma-aminobutyric acid (GABA) B receptor, 2" | Plasma Membrane | G-protein coupled receptor |
| GABRA1 | gamma-aminobutyric acid (GABA) A receptor, alpha 1" | Plasma Membrane | ion channel |
| GABRA5 | gamma-aminobutyric acid (GABA) A receptor, alpha 5" | Plasma Membrane | ion channel |
| GABRG1 | gamma-aminobutyric acid (GABA) A receptor, gamma 1 | Plasma Membrane | ion channel |
| GFAP | glial fibrillary acidic protein | Cytoplasm | other |
| GRIA2 | glutamate receptor, ionotropic, AMPA 2" | Plasma Membrane | ion channel |
| GRIA3 | glutamate receptor, ionotrophic, AMPA 3" | Plasma Membrane | ion channel |
| GRM1 | glutamate receptor, metabotropic 1" | Plasma Membrane | G-protein coupled receptor |
| HIF1A |  | Nucleus | transcription regulator |
| HTR2C | 5-hydroxytryptamine (serotonin) receptor 2C | Plasma Membrane | G-protein coupled receptor |
| IGF2 | insulin-like growth factor 2 (somatomedin A) | Extracellular Space | growth factor |
| IL1B | interleukin 1, beta" | Extracellular Space | cytokine |
| IL6 | interleukin 6 (interferon, beta 2)" | Extracellular Space | cytokine |
| INS |  | Extracellular Space | other |
| JUN | jun oncogene | Nucleus | transcription regulator |
| LRP1B | low density lipoprotein-related protein 1B (deleted in tumors) | Plasma Membrane | transmembrane receptor |
| MAPT | microtubule-associated protein tau | Cytoplasm | other |
| MME | membrane metallo-endopeptidase | Plasma Membrane | peptidase |
| MTHFR | 5, 10-methylenetetrahydrofolate reductase (NADPH)" | Cytoplasm | enzyme |
| NAV1 | neuron navigator 1 | Cytoplasm | enzyme |
| NPTX1 | neuronal pentraxin I | Extracellular Space | other |
| OGN | osteoglycin | Extracellular Space | growth factor |
| PLAT | plasminogen activator, tissue" | Extracellular Space | peptidase |
| PRKCA | protein kinase C, alpha" | Cytoplasm | kinase |
| PRKCD |  | Cytoplasm | kinase |
| PRKCE | protein kinase C, epsilon" | Cytoplasm | kinase |
| PRKCZ |  | Cytoplasm | kinase |
| PTGS2 | prostaglandin-endoperoxide synthase 2 (prostaglandin G/H synthase and cyclooxygenase) | Cytoplasm | enzyme |
| RPS6KB1 | ribosomal protein S6 kinase, 70kDa, polypeptide 1" | Cytoplasm | kinase |
| TLR4 | toll-like receptor 4 | Plasma Membrane | transmembrane receptor |
| TP73 | tumor protein p73 | Nucleus | transcription regulator |
