# Supplementary material for: Dysfunction of the mTOR pathway is a risk factor for Alzheimer’s disease
Source: Acta Neuropathol Commun. 2013 May 8;1:3. doi: 10.1186/2051-5960-1-3 (PMC3776211; doi:10.1186/2051-5960-1-3)
Supplement: Additional file 3 — Quality standards for the LDH assay. [file 2051-5960-1-3-S3.doc]

**Dysfunction of the mTOR pathway is a risk factor for Alzheimer’s disease**

**Sharon C Yates**^1^**, Amen Zafar**^1^**, Paul Hubbard**^1^**, Sheila Nagy**^1^**, Sarah Durant**^2^**, Roy Bicknell**^2^**, Gordon Wilcock**^3^**, Sharon Christie**^3^**, Margaret M Esiri^4^, A David Smith**^5^**, Zsuzsanna Nagy**^1*^

^1^ Neuropharmacology and Neurobiology, College of Medical and Dental Sciences, School of Clinical and Experimental Medicine, University of Birmingham, Birmingham, B15 2TT, UK.

^2^ Institute of Biomedical Research, College of Medical and Dental Sciences, University of Birmingham, Birmingham, B15 2TT, UK.

^3^ OPTIMA, University of Oxford, Level 4, John Radcliffe Hospital, Oxford, OX3 9DU, UK.

^4^ Department of Neuropathology, University of Oxford, Level 1, John Radcliffe Hospital, Oxford, OX3 9DU, UK.

^5^ Department of Pharmacology, University of Oxford, Mansfield Road, Oxford OX1 3QT, UK.

*** Corresponding Author:** Dr. Zsuzsanna Nagy; email: [z.nagy@bham.ac.uk](mailto:z.nagy@bham.ac.uk)

# Online resource 2 Supplementary methods

## Quality standards for the LDH assay

The LDH positive standard provided with the LDH assay kit (Promega) was used as the reference standard. This was calibrated to equivalent cell numbers using a purified human lymphocyte sample of known cell density. The calibration curve was set up using this positive reference standard in triplicates. For determination of cell numbers we relied strictly on the linear part of the calibration curve (R^2^>95% linear regression model). The level of detection (LOD) of the assay (significantly different from matrix) was found to be consistently around 3,000 cells (1:160,000 dilution of LDH positive control). In the linear ranger the lowest level of quantification (LLOQ) was 5,500 cells (1:80,000 dilution of LDH positive control) while the highest level of quantification (HLOQ) was 88,000 cells (1:5,000 dilution of LDH positive control) cells. The back-calculated cell numbers from the calibration curve show that the use of the calibration allows a very accurate assessment of cell numbers within the linear range (linear regression between actual and back-calculated cell numbers: R^2^=99.97%) and the difference between the actual cell number and back-calculated cell number was < 10% for the upper range and <20% at the lowest range.

## Quality standards for flow cytometry

In the optimization period we have established the cytometer settings that allowed the best discrimination of DNA content of cells. The gates for the single cell population and the non-dividing cell population were set up based on the FL2-H and LF2-W dot blot. The parameters for non-dividing cell population was confirmed from a lymphocyte sample that was not activated previously. The interpretation of cell cycle phases is based on the recommendations of MG Ormerod [31]. The shift of the position (mean and median) of the G1 and G2 peaks from the mode was less that 10% across the wells within one 96 well plate. The ratio between the Mean and Median of the G1 and G2 populations was between 1.8 and 2.2 (10% deviation either way from 2, measure of linearity). The CV of measured parameters was less than 10% in the technical replicates carried out for each patient sample.

## RNA quality and choice of arrays.

The quality of the RNA from the lymphocytes, as assessed on the Agilent 2100 Bioanalyser, was high (8-10 RIN) indicating suitability for microarrays.

The quality of the RNA from the post mortem brain tissue was poor (2-3 RIN), which made these samples unsuitable for some array platforms. The Agilent microarrays use 60-mer probes, with most genes represented by a single probe. Gene expression is derived from one probe for each gene, the same probe from each array [30]. The housekeeping genes used for normalization are located on the 3’-end of the RNA, which prevents degradation-related skewing of the data secondary to the normalization procedure. The Agilent microarrays have been desined to allow accurate measures even with highly degraded RNA (down to RIN values of 2) [22]. As long as the RNA quality is consistent across all samples [41], as was the case in this series of post mortem samples, the Agilent microarray is unlikely to give spurious false positive results., We are however aware of the facts that the degraded RNA will lead to a loss of sensitivity and consequently false negative results in some instances (especially short transcripts) [30]. With this caveat, the gene expression data from poor quality RNA can be accepted [21, 22, 30, 41]. For this study this means that we may not have detected the differential expression of all rapamycin-regulated genes in AD brain compared to control. However, we can be reasonable sure that the genes that were identified as differentially expressed are true positive findings.

## Validation of microarray results by Q-PCR

Six rapamycin-regulated genes that were shown to be differentially expressed by microarray in advanced AD brain compared to control were selected for validation by Q-PCR (Supplementary Table 1). Q-PCR protocols were designed with the Universal probe library design centre (Roche Diagnostic) for the selected genes and the housekeeping gene: beta-actin. The RNA samples used for validation were identical to those used for microarray and were converted to cDNA with the high capacity cDNA reverse transcription kit (Applied Biosystems). The cDNA was purified by precipitation with isopropanol (Sigma); and washed with ethanol (Sigma), prior to rehydration in nuclease free water (Qiagen). Each Q-PCR run included the samples-of-interest, two negative controls (water) and a cDNA standard curve (five serial dilutions starting with neat cDNA). Each 20µl Q-PCR reaction was composed of 2µl cDNA, 0.5µl Universal probe (Roche), 0.5µl each of 20µM forward and reverse primer (Sigma Genosys) and 2x Absolute Q-PCR mix (Thermoscientific). The samples were amplified by 15 minutes at 96ºC followed by 40 cycles of 96ºC for 15 sec, optimal annealing temperature (Supplementary Table 1) for 30 sec, and 72ºC for 30 sec. FAM output was read in the annealing phase.

Each of the cDNA standard curve serial dilutions was assigned an arbitrary copy number to allow construction of a standard curve. Q-PCR was considered fully optimized when the calculated standard curve copy number varied less than 10% from the assigned copy number. All gene results were normalized to the corresponding beta-actin result to allow quantitative comparison of samples.

Microarray analysis showed that eukaryotic translation initiation factor 4E (EIF4E), mitogen activated protein kinase 1 (MAPK1), gamma-aminobutyric acid B receptor 2 (GABBR2) and DAZ interacting protein 3 (DZIP3) were significantly down-regulated in advanced AD compared to control; whereas semaphorin 4C (SEMA4C) and serpin peptidase inhibitor, clade E, member 1 (SERPINE 1) were significantly up-regulated in advanced AD compared to control (Supplementary Figure 4). For all 6 genes, the direction of differential expression as determined by microarray was the same as that determined by Q-PCR, although the Q-PCR results failed to reach statistical significance (Supplementary Figure 4).
